# Late‐Stage Phenylation From [^13^C_6_] and [^2^H_5_]Benzene: A Versatile Tool for Stable Isotope Labeled MS Standards

**DOI:** 10.1002/chem.202503170

**Published:** 2026-01-20

**Authors:** Alexandre Labiche, Bouchaib Mouhsine, Dorian Dupommier, Louise Fogel, Frédéric Robert, David‐Alexandre Buisson, Frédéric Taran, Davide Audisio

**Affiliations:** ^1^ Service de Chimie Bio‐Organique et Marquage DMTS Université Paris‐Saclay, CEA Gif‐sur‐Yvette France; ^2^ Eurisotop Gif‐sur‐Yvette France

## Abstract

We explored aryl thianthrenation as a tool for directly incorporating multiple isotopes into molecular scaffolds starting from full isotopically labeled benzene. Stable isotope‐labeled (SIL) molecules are indispensable tools for investigating chemical and biological mechanisms and quantifying metabolites. However, the late‐stage incorporation of isotopically labeled benzene remains challenging and under‐investigated. This challenge stems from the direct functionalization of non‐substituted benzene, which remains a difficult task. The approach described in this work is based on sulfonium salts used as a source of [^13^C_6_] and [^2^H_5_]benzene under palladium catalysis conditions. This approach demonstrates its efficiency in C─C and C─S bond formation with different substrates. Additionally, this methodology was employed for the synthesis of poly‐substituted [^13^C_6_]benzene derivatives.

## Graphical abstract

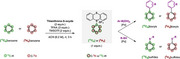

## 1 Introduction

Aromatic moieties are extensively found in commercialized drugs [1, 2, 3], representing a systematic structural feature in many medications included in the World Health Organization (WHO) model list of essential medicines [4]. It is well‐recognized that aromatic rings possess a significant impact on molecular reactivity, rigidity, metabolic stability, electronic distribution, hydrophobicity, and polarity [5]. In 2020, Stuart and coworkers assessed the benzenoid substitution patterns on 904 Food and Drug Administration (FDA) approved active pharmaceutical ingredient (API) and revealed that mono‐substituted phenyl rings represent the most frequently encountered pattern, while 1,4‐*para* substituted benzenoid rings are in second position [6].

**SCHEME 1:**
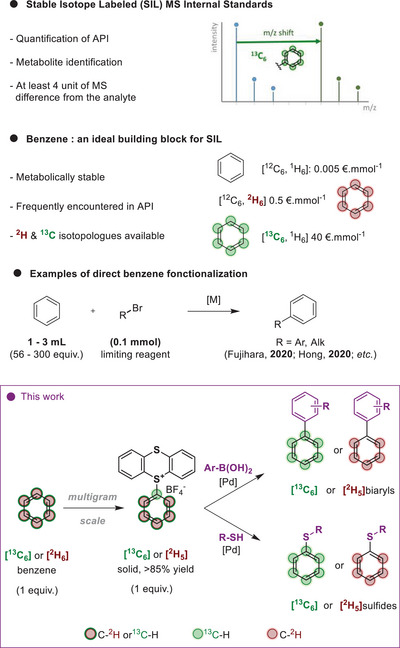
Isotopically labeled benzene for the preparation of SIL standards.

Isotope‐labeled aromatic compounds are powerful tools for advancing our understanding of organic and biological processes, particularly in the areas of mechanistic studies. Furthermore, stable isotope‐labeled (SIL) compounds are of particular utility in the field of metabolite quantification [7, 8]. They are essential for quantitative LC‐MS/MS analysis of analytes in complex matrices such as blood or urine. Due to the intrinsic similarity in physical and chemical properties between the analyte and its standard, they exhibit identical behavior in complex biological samples and ionization characteristics in LC‐MS. To fulfil SIL requirements, the target isotopologue shall possess a comfortable mass difference between the analyte and the SIL internal standard. Three to four units are commonly suggested to avoid isotopic massif overlapping, thus preventing quantification errors and facilitating data analysis [9]. In light of its metabolic stability and the potential for multiple isotope incorporation, ^13^C‐labeled aromatics are pillars for SIL preparation. However, the incorporation of a ^13^C‐labeled aromatic unit might reveal an insidious challenge, often laborious and time‐consuming [10, 11, 12, 13, 14]. Uniformly labeled substituted aromatic derivatives are synthesized from [^13^C_6_]benzene, the standard precursor for ^13^C‐labeled aromatics. Subsequent functionalization of [^13^C_6_]benzene, including functional group interconversion and additional substitutions [15, 16, 17, 18], allows to access the desired SILs. It should be noted that [^13^C_6_]benzene is a costly building block (Scheme 1), prepared from the trimerization of [^13^C]acetylene [19], which is itself derived from [^13^C]CO, the primary isotope source. [^2^H_6_]benzene, on the other hand, is usually accessed by hydrogen isotope exchange from unlabeled benzene in presence of a catalyst [20]. Ideally, a late‐stage direct C─H activation of labeled benzene would substantially reduce the number of steps and increase the overall efficiency of the synthesis. The direct C─H activation on benzene still stands as a challenge [21, 22, 23], as the absence of directing groups limits the breadth of available methodologies [24, 25, 26, 27]. To overcome such a limitation, benzene is often used in large excess, even as (co)solvent [28, 29, 30, 31, 32, 33]. While some methods exist for reducing benzene equivalence, their scope is narrowed to specific substitution patterns and functionalization, often restricting their potential applications [34, 35, 36, 37].

In 2019, Ritter and coworkers reported the site‐selective C─H functionalization of arenes by thianthrenation and their subsequent functionalization [38]. This reaction, which showcases a high *para*‐selectivity on electro‐donating substituted aromatic rings, has been extensively studied over the last years and allows to access to a large number of C─X substitutions (X = C, N, O, S, etc.) [39, 40, 41, 42, 43, 44, 45, 46]. Recognizing the potential of thianthrene chemistry for the preparation of SILs and valorization of unsubstituted benzene, herein, we disclose an optimized protocol, which allows to utilize [^13^C_6_] and [^2^H_6_]benzene as a limiting reagent for application in late‐stage phenylation via C─C and C─S palladium‐catalyzed coupling reactions. This procedure allowed the rapid synthesis of ^13^C‐labeled synthons, including pharmaceutical SIL and preliminary examples of 1,4‐, 1,3‐, and 1,2,3‐[^13^C_6_]benzene functionalization. We anticipate that this method will considerably streamline the synthesis of MS internal standards.

## 2 Results and Discussion

Aiming to develop a versatile, practical, and cost‐effective methodology for late‐stage phenylation, we anticipated that access to a phenyl thianthrenium salt (**1**) would be beneficial with respect to the corresponding phenyl halide derivatives, both in terms of selectivity (mono‐ vs. di‐ substitution), product purification, and isolation. Thus far, the thianthrenation of unsubstituted benzene has attracted limited attention. In 2019, Zhang and coworkers reported the synthesis of **1** in 34% isolated yield using 3 equiv. of benzene [47]. A subsequent report by the Ritter group allowed to increase the yield to 87%, but required a larger excess of 11 equiv. of benzene, and was carried out on a moderate reaction scale [48]. Given the cost considerations associated with accessing labeled starting materials, our initial study focused on optimizing the synthesis of **1** using benzene as the limiting reagent.

When thianthrene *S*‐oxide (**2**) was activated with Tf_2_O (1.2 equiv.) in DCM at room temperature after 3 h, 1 was obtained in 32% NMR yield (Table 1, entry 1). The decrease of thianthrene from 1 to 0.1 equiv. only slightly improved the yield (Table 1, entry 2). When the reaction concentration was increased to 1 M a 29% yield was observed (Table 1, entry 3), whereas a concentration of 0.05 M resulted in a yield of only 15% (Table 1, entry 4).

**TABLE 1:** Optimization of the reaction conditions for the synthesis of thianthrenium salt 1^a^.

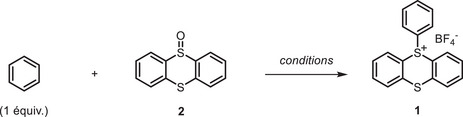					
Entry	2 (X equiv.)	Solvent (conc.)	T (°C)	Anhydride/acid	NMR Yield(%)
1	1.0	DCM(0.2 M)	25	Tf_2_O/‐	32
2	0.1	DCM(0.2 M)	25	Tf_2_O/‐	53
3	1.0	DCM(1 M)	25	Tf_2_O/‐	29
4	1.0	DCM(0.05 M)	25	Tf_2_O/‐	15
5^*^	1.0	DCM(0.05 M)	40	Tf_2_O/‐	12
6	1.0	ACN(0.2 M)	25	Tf_2_O/HBF_4_ OEt_2_	26
7	1.0	ACN(0.2 M)	25	TFAA/HBF_4_ OEt_2_	14
8	1.0	ACN(0.2 M)	25	TFAA/TMSOTf	59
9	2.0	ACN(0.2 M)	25	TFAA/TMSOTf	88
10	5.0	ACN(0.2 M)	25	TFAA/TMSOTf	>98

^*^ The reaction was performed under reflux conditions.

Abbreviations: ACN, acetonitrile; DCM, dichloromethane; Tf_2_O, trifluoromethanesulfonic anhydride; TFAA, trifluoroacetic anhydride.

^a^ Unless otherwise noted, the reaction was performed with benzene (0.3 mmol), **2** (0.1–5 equiv.) and additives [Tf_2_O (1.2 equiv.), TFAA (3 equiv.), TMSOTf (2.0 equiv.), HBF_4_.OEt_2_ (2.0 equiv.)], in the indicated solvent (0.3–6 mL) for 3 h.

The temperature had no significant impact on the reaction, as similar results were obtained at reflux (Table 1, entry 5). The addition of HBF_4_.OEt_2_ (2 equiv.) with Tf_2_O (1.2 equiv.) in acetonitrile was inconsequential (Table 1, entry 6). On the other hand, only 14% yield was obtained using TFAA (3 equiv.) in place of Tf_2_O (Table 1, entry 7). Pleasingly, a substantial improvement was observed when HBF_4_.OEt_2_ was replaced with TMSOTf (2 equiv., Table 1, entry 8). Gratifyingly, we could increase the yield to 88% and up to 98% using 2 and 5 equiv. of **2**, respectively (Table 1, entries 9 and 10). While nearly quantitative yields were achieved with 5 equiv. of **2**, we deemed this amount excessive. Therefore, we adopted the conditions outlined in Table 1, entry 9, as optimal.

**SCHEME 2:**
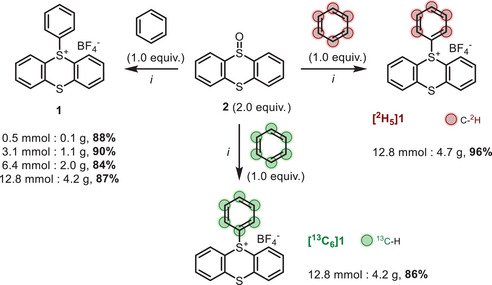
Gram‐scale synthesis of **1**, **[^13^C_6_]1** and **[^2^H_5_]1**. Reaction conditions: (*i*) benzene (1 equiv.), **2** (2 equiv.), TFAA (3 equiv.), TMSOTf (2 equiv.), ACN (0.2 M), from 0 °C to 25 °C, 3 h. Crude products were precipitated in DCM/Et_2_O. For detailed procedure, see Supporting Information (General Procedure 1, page S7).

The reaction proved reproducible on a 0.5 mmol scale, yielding **1** in 88% yield. To evaluate the robustness of the process, we systematically increased the reaction scale to 3.1, 6.4, and 12.8 mmols. Encouragingly, yields remained consistent, ranging from 84% to 90%, regardless of scale. Subsequently, we successfully extended this protocol to the [^13^C_6_]benzene and [^2^H_6_]benzene isotopologues, which afforded similar yields to unlabeled benzene (Scheme 2). This procedure offers significant advantages over state‐of‐the‐art benzene halogenation methods [49, 50], including selective mono‐functionalization, scalability, and facile product isolation via simple filtration of a solid salt.

**SCHEME 3:**
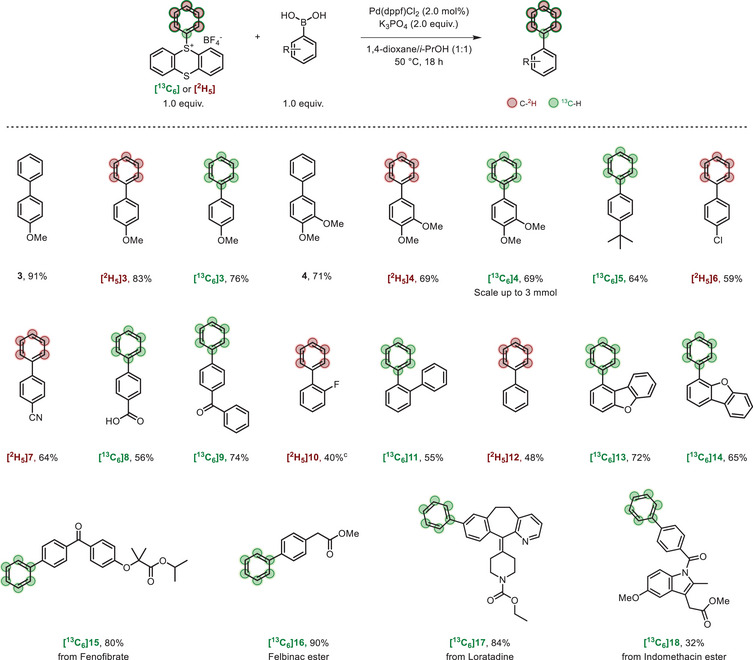
Synthesis of labeled biaryls via Suzuki–Miyaura coupling reactions from **[^13^C_6_]1** and **[^2^H_5_]1**.^a,b a^ Reaction conditions: **1** (0.3 mmol), boronic acid (0.3 mmol) and Pd(dppf)Cl_2_ (2 mol%), K_3_PO_4_ (0.6 mmol) in dioxane/*i*‐PrOH (1/1, 0.054 M, 5.6 mL) at 50°C for 18 h. ^b^ Isolated yield. ^c^ 2 equiv. of **[^2^H_5_]1** were used. ^d^ BPin version was used as the starting material.

With an effective synthetic access to uniformly labeled **[^2^H_5_]1** and **[^13^C_6_]1**, we next focused on the late‐stage phenylation using the classic conditions of the Suzuki–Miyaura cross‐coupling reaction [51]. The scope was explored with different substrates, employing Pd(dppf)Cl_2_ (2.0 mol%), K_3_PO_4_ (2 equiv.) in 1,4‐dioxane/*i*‐PrOH (1/1) at 50 °C. Pleasingly, excellent yields were achieved with *para*‐methoxy phenyl boronic acid using ^12^C, ^2^H_6_‐ and ^13^C_6_‐labeled benzene, yielding 91% (Scheme 3, compound **3**), 83% ([**^2^H_5_]3**), and 76% (**[^13^C_6_]3**), respectively. A slight decrease in yield was observed when 3,4‐phenyl boronic acid was used, resulting in isolated yields of 71% (**4**), 69% (**[^2^H_5_]4**), and 69% (**[^13^C_6_]4**). The use of 4‐*tert*‐butylphenylboronic acid yielded **[^13^C_6_]5** in 64% yield. Substrates bearing electron‐withdrawing groups were also examined. The presence of a chlorine, nitrile, carboxylic acid, or ketone at the *para* position of the phenylboronic acid was tolerated, yielding **[^2^H_5_]6**, **[^2^H_5_]7**, **[^13^C_6_]8**, and **[^13^C_6_]9** with good, isolated yields of 59%, 64%, 56%, and 74%, respectively. However, low yield (40%) was obtained for the synthesis of **[^2^H_5_]10,** even when using 2 equiv. of **[^2^H_5_]1**. Good results were obtained with substrates bearing electron‐neutral groups, yielding **[^13^C_6_]11** and **[^2^H_5_]12** with yields of 55% and 48%, respectively. Dibenzofuran, a hetero‐aromatic often found in bioactive products, proved to be an interesting substrate, providing **[^13^C_6_]13** and **[^13^C_6_]14** with yields of 72% and 65%. Finally, biologically active derivatives such as Fenofibrate analogue **[^13^C_6_]15**, Felbinac methyl ester **[^13^C_6_]16**, Loratadine analogue **[^13^C_6_]17**, and Indomethacine methyl ester **[^13^C_6_]18** were obtained with yields between 32% and 90%. Unfortunately, neither (biphenyl)‐4‐ylboronic acid, (bromoanthracen‐9‐yl)boronic acid, 1,4‐phenylenediboronic acid, (2‐(9H‐carbazol‐9‐yl)phenyl)boronic acid, and Haloperidol analogue allowed the preparation of their respective derivatives (respectively **[^13^C_6_]I**, **[^13^C_6_]II**, **[^13^C_6_]III**, **[^13^C_6_]IV**, and **[^13^C_6_]V**, see Supporting Information, p. S35).

**SCHEME 4:**
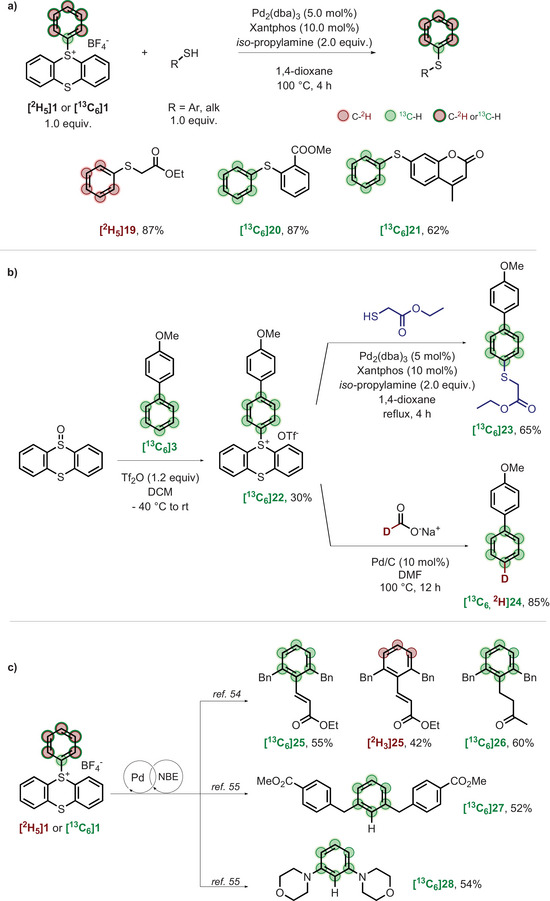
(a) Synthesis of aryl thioethers from labeled **1**. (b) Synthesis of labeled 1,4‐substituted benzene. (c) Access to 1,2,3‐trisubstituted benzene. For detailed procedure, see Supporting Information. NBE, Norbornene.

As aryl sulfides are key substitutions found in biologically active compounds, we further investigated C─S bond formation. While the thioetherification of aryl thianthrenium salts was explored by Ritter [52] and Molander [42] by photoredox chemistry strategies, we aimed to investigate a palladium‐catalyzed process. Labeled **1** was thus reacted with aromatic and aliphatic thiols using Pd_2_(dba)_3_ and Xantphos in 1,4‐dioxane (Scheme 4a).

Using ethyl 2‐mercaptoacetate as starting material, **[^2^H_5_]19** was obtained in 87% yield. Methyl 2‐mercaptobenzoate was transformed to **[^13^C_6_]20** with a very good yield of 87%. Finally, coumarin derivative **[^13^C_6_]21** was synthesized with 62% yield.

Next, we directed our attention to the synthesis of 1,4‐disubstituted benzenes, a prevalent substitution pattern in drug molecules (Scheme 4b) [6]. Thianthrenation of labeled **[^13^C_6_]3** allowed to obtain the *para*‐methoxy biphenyl sulfonium **[^13^C_6_]22** (Scheme 4b). The salt was then successfully used for the preparation of the 1,4‐substituted aryl thioether **[^13^C_6_]23**. Using the procedure developed by Zhao and coworkers [53], **[^13^C_6_]22** was also successfully used in a deuteration reaction using D‐sodium format to synthesize **[^13^C_6,_^2^H]24** with a yield of 85%. As a corollary, we applied the Catellani‐based *ortho*/*ipso* C‐H difunctionalization of aryl thianthrenium salts reported by Besset and coauthors [54] and Liu, Liang, and coauthors [55].

Starting from **[^13^C_6_]1**, we were able to prepare the *ipso*‐olefinated *ortho*‐alkylated **[^13^C_6_]25** and **[^2^H_3_]25** with good 55% and 42% yields, respectively, as well as the *ipso*‐alkylated **[^13^C_6_]26** in a good 60% yield through an *ipso* walking‐chain alkylation. Care was also taken to vary the *ipso* functionalization with the *ortho*‐alkylated *ipso*‐hydrogenated **[^13^C_6_]27**, isolated with a 52% yield. Finally, we changed the nature of the *ortho*‐functionalization by introducing morpholine moieties, pleasingly yielding to the diaminated **[^13^C_6_]28** with a 54% yield.

## 3 Conclusion

In conclusion, this study presents a novel approach for late‐stage phenylation suitable for the preparation of ^2^H and ^13^C SILs having M+5 and M+6 mass differences. We optimized the synthesis of a labeled sulfonium salt from non‐substituted benzene. This procedure allows to obtain phenyl thianthrenium salts on multiple gram scale, utilizing labeled benzene as a limiting reagent. Subsequently, we employed these labeled salts as building blocks to prepare a library of labeled biaryl compounds through Suzuki coupling reactions, including two bioactive molecules. Our investigations were extended to the synthesis of labeled thioethers via palladium‐catalyzed reactions with thiols. Furthermore, labeled 1,3‐, 1,4‐, and 1,2,3‐ poly‐substituted benzene rings were synthesized, emphasizing the versatility of labeled arylthianthrenium salts to access to different substitution patterns.

This protocol represents a significant advancement in cost‐efficiency over the state‐of‐the‐art methods by using benzene as the limiting reagent and drastically reduces the consumption of expensive [^13^C_6_]benzene. In terms of sustainability, the method improves atom economy and streamlines downstream processing. The formation of stable, solid phenyl thianthrenium salts enables facile isolation via filtration, avoiding the wasteful and energy‐intensive purification steps typical of volatile labeled halides. Consequently, this methodology offers a practical and sustainable alternative for the streamlined production of MS internal standards.

## Conflicts of Interest

The authors declare no competing interests.

## Supporting information




**Supporting File 1**: chem70698‐sup‐0001‐SuppMat.pdf
