# Supplementary material for: Late‐Stage Phenylation From [13C6] and [2H5]Benzene: A Versatile Tool for Stable Isotope Labeled MS Standards
Source: Chemistry. 2026 Jan 20;32(12):e03170. doi: 10.1002/chem.202503170 (PMC13037350; doi:10.1002/chem.202503170)
Supplement: Supplementary file 1 — Supporting File 1: chem70698‐sup‐0001‐SuppMat.pdf [file CHEM-32-e03170-s001.pdf]

Late-Stage Phenylation from [$^{13}C_6$] and [$^{2}H_5$]Benzene: a Versatile Tool for Stable Isotope Labeled MS Standards

Alexandre Labiche,[1‡] Bouchaib Mouhsine,[1‡] Dorian Dupommier,[1] Louise Fogel,[1] Frédéric Robert,[2] David-Alexandre Buisson,[1] Frédéric Taran,[1] Davide Audisio[1*]

[1] Université Paris-Saclay, CEA, Service de Chimie Bio-organique et Marquage, DMTS, F-91191, Gif-sur-Yvette, France.

[2] Eurisotop, F-91191, Gif-sur-Yvette, France

Supporting Information

Table of contents

1. Materials and Methods 3

1.1. Reactants and solvents 3

1.2. Analysis 3

1.3. Purifications 4

2. Optimisation and general procedures 5

2.1. Optimisation for the synthesis of phenylthianthrenium salts 5

2.2. General procedures 7

2.2.1. General procedure GP1 7

2.2.2. General procedure GP2: synthesis of biaryls 7

2.2.3. General procedure GP3: synthesis of thioethers 8

2.2.4. General procedure GP4: Polyfunctionalization of arenes 8

3. Characterization of [$^2H_5$] and [$^{13}C_6$] labeled arenes in NMR analysis 9

4. Experimental section 14

4.1. Experimental procedure for the synthesis of thianthrene-S-oxide 14

4.2. Experimental procedure for the synthesis of thianthrenium salts 15

5. NMR Spectra 49

6. Reference 95

1. Materials and Methods

1.1. Reactants and solvents

Commercially available chemicals were purchased from ABCR, Acros Organics, Merck (Sigma-Aldrich), Alfa Aesar, Combi-Blocks, Carbolution, Fluorochem, BLDpharm and TCI Europe and used as received unless otherwise stated. Stable isotopically labeled reagents (*i.e.* ^{13}C, ^{2}H) were purchased from Eurisotop (France). The following solvents were dried by distillation over the drying agents indicated in parentheses: THF (Sodium), Dichloromethane (CaH_2). Additional anhydrous solvents were purchased from Acros Organics, Merck (SigmaAldrich), Alfa Aesar and stored over molecular sieves under an argon atmosphere. All air- and moisture-sensitive experiments were carried out in an mBraun LabMaster DP inert argon atmosphere glovebox and mBraun unilab plus eco.

1.2. Analysis

Reactions were monitored by *Thin Layer Chromatography (TLC)* carried out on silica 0.25 mm (60 F254, Merck) using UV light as visualizing agent. For staining, the TLC plates were dipped into a solution of Ceric Ammonium Molybdate (100 mL of H_2SO_4 80%, 4 g ceric sulfate, 17.28 g molybdenum trioxide, 14 mL NH_4OH in 14 mL of water, 548 mL of water) or into a solution of Ninhydrine (1.5 g of ninhydrine, 5 mL of AcOH, 500 mL EtOH 95%) and developed with a heat gun.

Nuclear Magnetic Resonance (NMR) Spectroscopy: ^{1}H NMR (400 MHz), ^{2}H NMR (61 MHz), ^{13}C NMR (100 MHz), and ^{19}F NMR (376 MHz) were measured on a Brucker Avance 400 MHz spectrometer. Chemical shifts are reported in parts per million (ppm) downfield from residual solvents peaks or from $CFCl_3$ (δ = 0 ppm) for ^{19}F NMR spectra and coupling constants are reported as Hertz (Hz). Splitting patterns are designated as singlet (s), broad singlet (br. s), doublet (d), triplet (t), quartet (q), quintet (quint), sextuplet (sx), multiplet (m). Splitting patterns that could not be interpreted or easily visualized are designated as multiplet (m). For NMR yield: dibromomethane (1 equiv.) is used as an internal standard.

Electrospray mass spectra were obtained using an ESI-Quadripole autopurify, Waters (pump: 2545, mass: ZQ2000) mass Spectrometer.

LC-MS spectra were recorded on a Waters Acquity UPLC® equipped PDA eλ Detector and SQ Detector 2, mobile phase A: H_2O + 0.1% formic acid, mobile phase B: acetonitrile + 0.1% formic acid.

High-Resolution Mass Spectra (HRMS) were performed on a Waters Xevo® G2-XS QTof mass spectrometer.

Infrared spectra (IR) were obtained on a Perkin Elmer UATR TWO FTIR spectrophotometer and are reported as wavelength numbers (cm^{-1}).

Melting points (Mp) were obtained on a BÜCHI Melting Point B-545 and are reported in °C.

1.3. Purifications

Flash chromatography were performed on silica gel (Merck Kieselgel 60, grading 40-63 µm) or using automate Puriflash XS 520 Plus with pre-packed column RediSep® Rf (grading 35-70 µm).

2. Optimisation and general procedures

2.1. Optimisation for the synthesis of phenylthianthrenium salts

The optimisation was performed following the general procedure **GP1**.

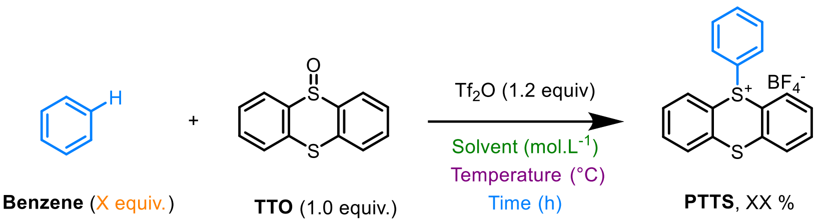

ENTREE	BENZENE (X EQUIV.)	SOLVENT (mol.L⁻¹)	TEMPERATURE (°C)	TIME (h)	NMR YIELD (%)[a]
1	1	DCM (0.2)	rt	3	32
2	2	DCM (0.2)	rt	3	37
3	4	DCM (0.2)	rt	3	41
4	7	DCM (0.2)	rt	3	40
5	10	DCM (0.2)	rt	3	53
6	1	DCM (1)	rt	3	29
7	1	DCM (0.4)	rt	3	31
8	1	DCM (0.05)	rt	3	15
9	1	DCM (0.02)	rt	3	11
10	1	DCM (0.05)	80	3	12
11	1	DCM (0.2)	rt	15 min	35
12	1	DCM (0.2)	rt	1	35
13	1	DCM (0.2)	rt	6	27
14	1	DCM (0.2)	rt	12	28
15	1	THF (0.2)	rt	15 min	0
16	1	CHCl3 (0.2)	rt	15 min	24
17	1	ACN (0.2)	rt	15 min	20
18	1	$C_2H_4Cl_2$ (0.2)	rt	15 min	30
19	1	C_6F_6 (0.2)	rt	15 min	15

Table S1: Conditions: **a)** ^{1}H NMR Yield using dibromomethane (1 equiv., 7 µL) as internal standard.

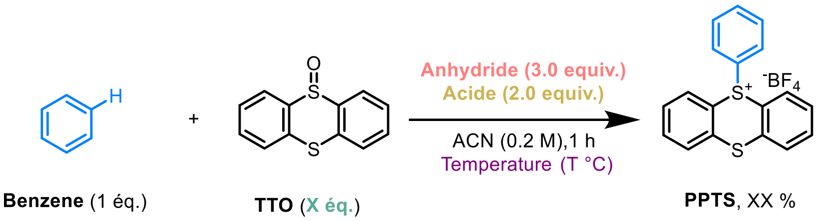

ENTREE	ANHYDRIDE	ACIDE	TEMPERATURE (°C)	TTO (X EQUIV.)	NMR YIELD (%)[a]
**1**	**Tf_2O**	$HBF_4.OEt_2$	25	1	**26**
**2**[b]	**Tf_2O**	$HBF_4.OEt_2$	**50**	1	**26**
**3**	TFAA	**HBF4.OEt_2**	25	1	**14**
**4**[b]	TFAA	HBF4.OEt_2	**50**	1	**15**
**5**[c]	-	**H_2SO_4**	**100**	1	**0**
**6**	TFAA	**TMSOTf**	25	1	**59**
**7**[d]	TFAA	TMSOTf	25	**1.5**	**78**
**8**[d]	TFAA	TMSOTf	25	**2**	88
**9**[d]	TFAA	TMSOTf	25	**3**	**89**
**10**[d]	TFAA	TMSOTf	25	**5**	**>98%**
**11**[e]	TFAA	TMSOTf	25	**2**	88%
**12**[f]	TFAA	TMSOTf	25	**2, without ACN**	**47**

Conditions : **a)** NMR Yield using dibromomethane (1 equiv., 7 µL) as internal standard, **b)** 7 h, **c)** 24 h, **d)** The amount of TFAA was used relative to TTO (3 equiv. relative to TTO), The amount of TMSOTF was used relative to TTO (2 equiv. relative to TTO), **e)** The amount of TFAA was used relative to benzene (3 equiv. relative to benzene), The amount of TMSOTF was used relative to benzene (2 equiv. relative to benzene), **f)** Without solvent.

2.2. General procedures

2.2.1. General procedure GP1

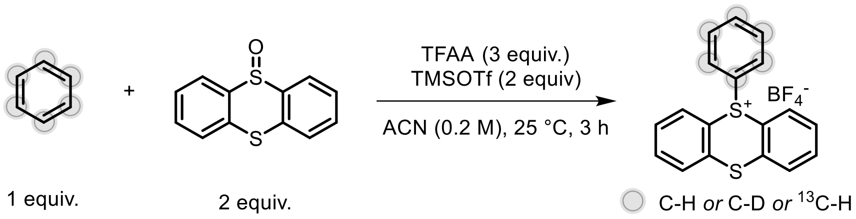

In a round bottom flask (RBF), benzene (1 equiv.) was added to a solution of thianthrene *S*-oxide (2 equiv.) dissolved in acetonitrile (0.2 M). After cooling down the mixture at 0 °C (ice/water bath), the adequate activating system [Tf_2O (1.2 equiv.) for the optimisation or TFAA (3 equiv.) and TMSOTf (2 equiv.) for the optimized conditions] were added and the mixture was stirred during 15 minutes. Then, the reaction was warmed up to room temperature and stirred during 3 h. Next, the crude reaction mixture was diluted adding DCM and extracted sequentially with $NaHCO_3$ (sat.) and an aqueous $NaBF_4$ solution (10% w/v). The organic layer is then dried by $MgSO_4$ and evaporated. Finally, the product was obtained by precipitation in DCM/Et_2O.

2.2.2. General procedure GP2: synthesis of biaryls

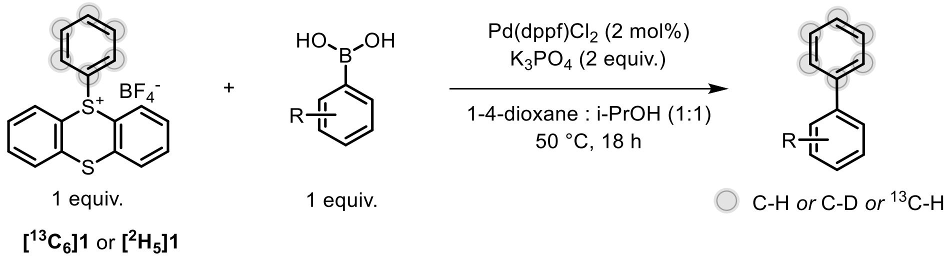

Under an ambient atmosphere, a 10 mL sealed tube equipped with a magnetic stir bar was charged with **[$^{13}C_6$]1** or **[$^{2}H_5$]1** (1.0 equiv.), boronic acid (1.0 equiv.), Pd(dppf)Cl_2 (2 mol%), K_3PO_4 (2.0 equiv.) and *i*-PrOH:1,4-dioxane (1:1, C = 0.054 M, 5,6 mL). The mixture was degassed by purging with argon for 5 minutes. Subsequently, mixture was stirred at 50 °C for 18 h. Subsequently, the solvents of the crude reaction mixture were removed under reduced pressure and the residue was purified by column chromatography on silica gel.

2.2.3. General procedure GP3: synthesis of thioethers

To a pressure tube (4 mL) equipped with a magnetic stir bar were added (**[$^{13}C_6$]24**) (1.0 equiv.), $Pd_2(dba)_3$ (5 mol%) and Xantphos (10 mol%). The tube was evacuated and backfilled with nitrogen. di-*iso*-propylamine (2.0 equiv.), dioxane (0.2 M) and thiol (2.0 equiv.) were added and the mixture was degassed by purging with argon for 5 minutes. Finally, the tube was sealed and stirred at 100 °C for 12 h. Subsequently, the solvent of the crude reaction mixture was removed under reduced pressure and the residue was purified by column chromatography on silica gel.

2.2.4. General procedure GP4: Polyfunctionalization of arenes

According to the slightly modified described procedure, an oven-dried 10 mL microwave tube was sequentially charged with the arylthianthrenium salt (0.20 mmol, 1.0 equiv.), $Pd(OAc)_2$ (4.5 mg, 0.02 mmol, 10 mol%), tris(2- furyl)phosphine (TFP, 11.6 mg, 0.05 mmol, 25 mol%), 2-hydroxy-5- trifluoromethylpyridine (6.5 mg, 0.04 mmol, 20 mol%), 5-norbornene-2-carbonitrile (119.0 mg, 1.00 mmol, 5.0 equiv.) and Cu_2O (42.9 mg, 0.30 mmol, 1.5 equiv.). The tube was then evacuated and refilled with argon three times, charged with the terminating reagent (0.3 mmol, 1.5 equiv.), the electrophile (0.60 mmol, 3.0 equiv.) and MeCN (4 mL, c = 0.05 M) under an argon atmosphere. The tube was then tightly sealed and heated under vigorous stirring at 120 °C for 14 h in a pre-heated pie-block. The reaction mixture was then allowed to cool down to room temperature, filtered over a short silica pad (eluent: EtOAc) and the solvents were removed under reduced pressure. The residue was then directly purified by column chromatography on silica gel, flash chromatography on reverse phase or preparative thin layer chromatography (PTLC) to afford the desired product.

3. Characterization of [$^2H_5$] and [$^{13}C_6$] labeled arenes in NMR analysis

The characterization of fully deuterated and ^{13}C-labeled aromatics is less common in the literature. During this study, we encountered challenges in describing the ^{1}H and ^{13}C NMR spectra of such labeled compounds. To aid the reader in understanding potential issues encountered when characterizing these compounds using ^{1}H and ^{13}C NMR spectroscopy, this section provides a brief overview of our approach.

Carbons bonded to deuterium do not exhibit a Nuclear Overhauser Effect (NOE) and are less amenable to polarization transfer techniques for increasing signal-to-noise ratios. Due to its spin of 1, deuterium couples with carbon, resulting in each ^{13}C-D signal appearing as a triplet. Furthermore, deuterium substitution increases the relaxation time of the directly bonded carbon, further reducing signal intensity. As a result, ^{13}C-D signals in deuterated arenes are particularly challenging to observe and can easily be mistaken for a complete absence of signal (Figure S1, S2).[1,2,3]

Concerning [$^{13}C_6$]arenes, the natural abundance of ^{13}C is approximately 1%. Routine analyses benefit from the Nuclear Overhauser Effect (NOE); consequently, the ^{13}C-^{13}C signals of [$^{13}C_6$]arenes are unusually intense, which can diminish the signal-to-noise ratio for unlabeled carbon signals. Furthermore, when six ^{13}C atoms are present in an aromatic ring, each ^{13}C atom can couple with neighboring ^{13}C atoms, complicating the multiplicity of the signals (Figure S1, S2).

For [$^{13}C_6$]arenes, in order to simplify the interpretation of the proton NMRs (^{1}H NMR), ^{13}C decoupling was perfomed (^{1}HNMR {^{13}C}).

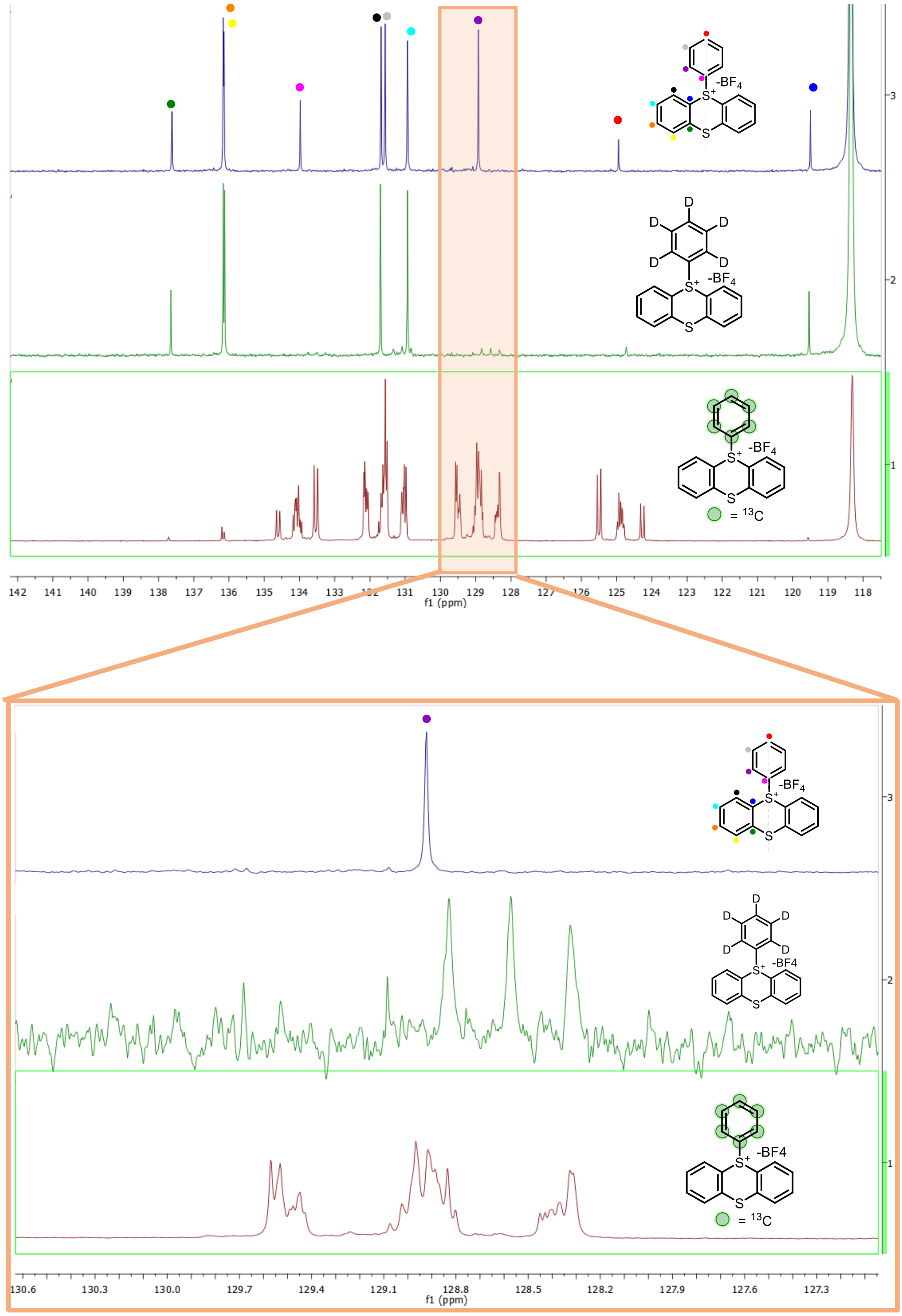

Figure S1. Superposition of ^{13}C-NMR (100 MHz, CD_3CN) of **1**, **[$^2H_5$]1** and **[$^{13}C_6$]1** (top) and a zoom on C_{sp2} signal at 128.9 ppm with different labeling (bottom)

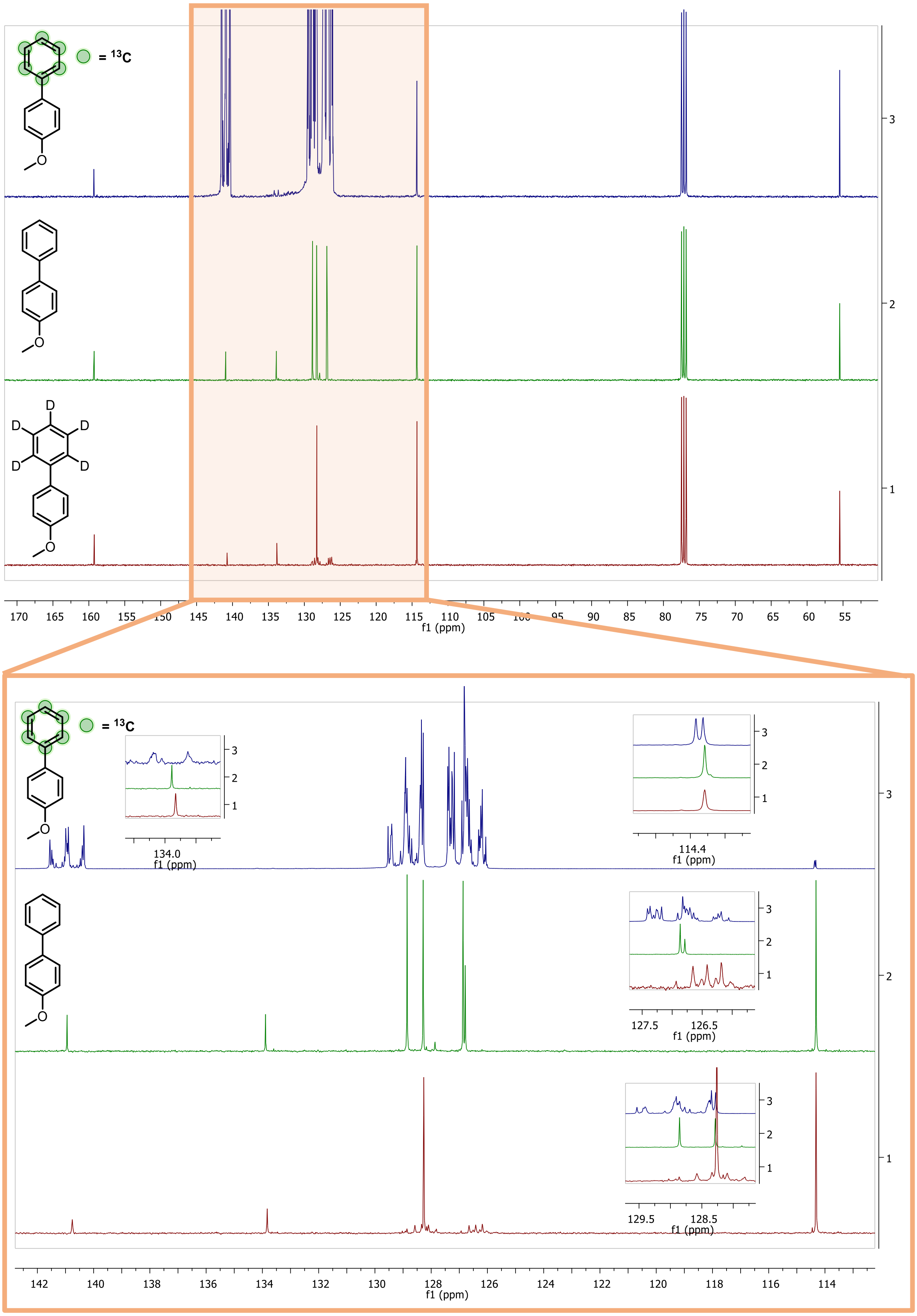

Figure S2. Superposition of ^{13}C-NMR (100 MHz, CD_3CN) of **[$^{13}C_6$]3**, **3** and **[$^2H_5$]3** (top) and a zoom on aromatic part of the molecules with several over zoom to showcase ^{13}C-H, C-H and C-D influence on the same position (bottom)

Preparation of starting materials:

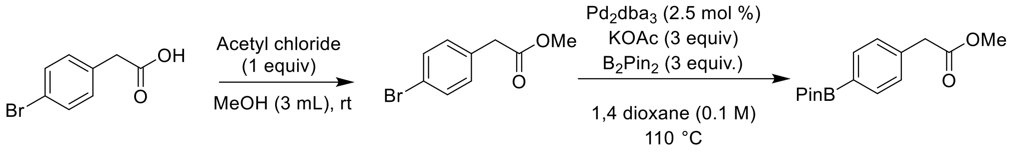

In a round bottom flask (50 mL), 2-(4-bromophenyl)acetic acid (1.1 g, 5 mmol, 1 equiv.), acetyl chloride (0.35 mL, 5 mmol, 1 equiv.) were placed and methanol (3 mL, 1.7 M) were added. The mixture was stirred at room temperature until complete consumption of the acid was observed by TLC. Methanol was removed *in vacuo* to give pure methyl 2-(4-bromophenyl)acetate (1.1 g, 94%). The product was engaged in the second step without further purification.

To a pressure tube (8 mL) equipped with a magnetic stir bar were added, methyl 2-(4-bromophenyl)acetate(45.8 mg, 0.2 mmol, 1 equiv.), B_2Pin_2 (151.1 mg, 0.6 mmol, 3 equiv.) $Pd_2(dba)_3$ (4.6 mg, 2.5 mol%), KOAc (176.4mg, 3 equiv.) were placed and 1,4-dioxane (2 mL, 0.1 M) was added. The mixture was stirred at 110 °C until complete consumption of the starting material was observed by TLC. The mixture was filtered and the solvent evaporated, 5 mL of water was added to the product and evaporated in order to eliminate the bis-pinacol. The product was then purified on a column chromatography on gel silica (DCM : ethyl acetate (5:95)) to give methyl 2-(4-(bis(4,4,5,5-tetramethyl-1,3-dioxolan-2-yl)boraneyl)phenyl)acetate as a white solid (50%, 42 mg). The NMR analysis was compatible with literature.

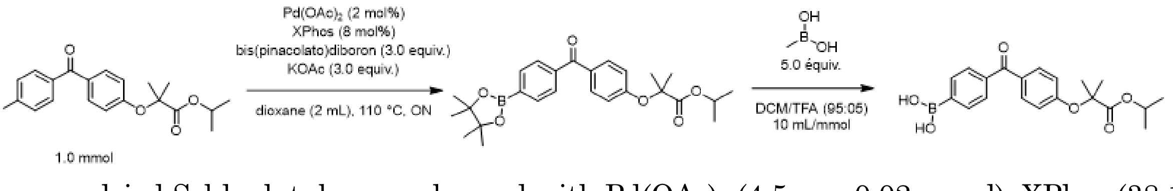

An oven-dried Schlenk tube was charged with $Pd(OAc)_2$ (4.5 mg, 0.02 mmol), XPhos (38.1 mg, 0.08 mmol), bis(pinacolato)diboron (761.8 mg, 3.00 mmol), isopropyl 2-(4-(4-chlorobenzoyl)phenoxy)-2-methylpropanoate (374.9 mg, 1.00 mmol) and KOAc (294.4 mg, 3.00 mmol). The Schlenk tube was capped with a rubber septum and then evacuated and backfilled with argon (this sequence was carried out two times). 1,4-Dioxane (0.50 mL) was added via syringe, through the septum. The septum was then replaced with a Teflon screwcap and the Schlenk tube was sealed. The reaction mixture was heated to 110 °C for overnight. At this point the reaction mixture was allowed to cool to room

temperature. The reaction solution was then filtered through a thin pad of celite (eluting with ethyl acetate) and the eluent was concentrated under reduced pressure. The crude material so obtained was purified via flash chromatography on silica gel (gradient Hexane 100 % Hexane/EA 90/10) to afford Isopropyl-2-methyl-2-(4-(4-(4,4,5,5-tetramethyl-1,3,2-dioxaborolan-2-yl)benzoyl)phenoxy)propanoate in 62% yield (288.6 mg, 0.6 mmol).[4] The product was engaged in the second step without further purification.

To a small round-bottom flask Isopropyl-2-methyl-2-(4-(4-(4,4,5,5-tetramethyl-1,3,2-dioxaborolan-2-yl)benzoyl)phenoxy)propanoate (1.0 equiv.) and methylboronic acid (5.0 equiv.) were added and dissolved in a solution of trifluoroacetic acid (5% in methylene chloride, 10 mL/mmol). After full conversion, all volatile compounds were evaporated at 40°C water bath temperature. To avoid mixed anhydride formation, the residue was redissolved in 0.1 N HCl (~10 mL/mmol), the mixture was evaporated and dried in vacuo to afford (4-(4-((1-isopropoxy-2-methyl-1-oxopropan-2-yl)oxy)benzoyl)phenyl)boronic acid as a white solid in 82% yield (169.2 mg, 0.3 mmol).[5] The NMR analysis was in agreement with the literature.

4. Experimental section

4.1. Experimental procedure for the synthesis of thianthrene-S-oxide

Thianthrene S-oxide (TTO)

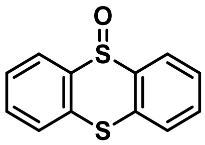

$C_{12}H_8OS_2$
MW: 232 g.mol^{-1}
Yield: 91%
White solid

In a 1 L RBF, to a solution of thianthrene (21.6 g, 1.0 equiv., 100 mmol) dissolved in DCM (286 mL, 0.35 M) were successively added $Fe(NO_3)_3.9H_2O$ (60.6 g, 1.5 equiv., 150 mmol), NaBr (0.72 g, 0.07 equiv., 7.0 mmol) and TFA (9.2 mL, 1.2 equiv., 120 mmol). During the addition of TFA, the mixture became, first purple then red. After 1h, the full conversion was checking by TLC and the mixture was diluted by addition of DCM before being washed with a saturated aqueous $NaHCO_3$ solution (4 times), brine and water. The organic layer wasthen dried by $MgSO_4$ and evaporated to give the expected product as a white solid (21.2 g, 91 mmol, 91%). Results were in agreement with the literature.[6]

^{1}H NMR (400 MHz, $CDCl_3$) δ = 7.93 (dd, J = 7.8, 1.1 Hz, 2H), 7.62 (dd, J = 7.7, 0.8 Hz, 2H), 7.56 (td, J = 7.6, 1.2, 2H), 7.44 (td, J = 7.6, 1.4 Hz, 2H).

This procedure was applied on different scales with consistent yields.

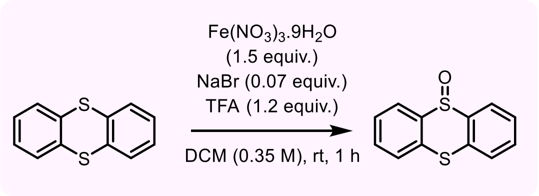

**TT** (1 equiv., 0.5 mmol)	**TTO**, 110 mg, **96%**
(4.6 mmol)	985 mg, **98%**
(12 mmol)	2.6 g, **95%**
(20 mmol)	4.4 g, **94%**
(40 mmol)	8.6 g, **88%**
(80 mmol)	17.6 g, **95%**
(100 mmol)	21.2 g, **91%**

4.2. Experimental procedure for the synthesis of thianthrenium salts

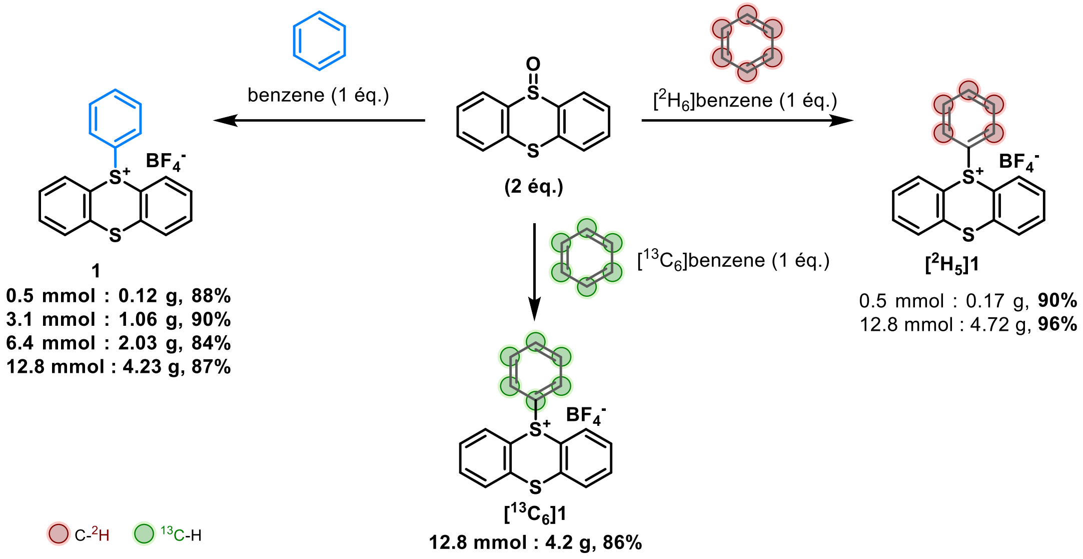

5-Phenyl-5*H*-thianthren-5-ium tetrafluoroborate (1)

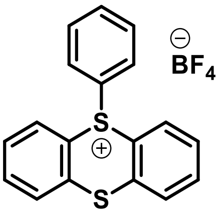

1

$C_{18}H_{13}BF_4S_2$
MW: 380.05 g.mol^{-1}
Yield: 90%
Light-grey solid

The 5-phenyl-5*H*-thianthren-5-ium tetrafluoroborate (**1**) was prepared according to the general procedure GP1, using benzene (276 µL, 12.8 mmol), thianthrene-*S*-oxide (TTO) (1.51 g, 6.2 mmol), TFAA (1.29 mL, 9.3 mmol) and TMSOTf (1.12 mL, 6.2 mmol) in acetonitrile (15.5 mL, 0.2 M) for 3 hours at rt. After precipitation, the compound was obtained as a light-grey solid (1.06 g, 2.79 mmol, 90%).

^{1}H NMR (400 MHz, CD_3CN) δ 8.38 (dd, *J* = 7.8, 1.5 Hz, 2H), 7.97 (m, 2H), 7.90 (td, *J* = 7.7, 1.4 Hz, 2H), 7.83 (m, 2H), 7.61 (m, 1H), 7.49 (m, 2H), 7.12 (m, 2H).

^{13}C NMR (100 MHz, CD_3CN) δ 137.6 (2C), 136.2 (2C), 136.4 (2C), 134.0 (2C), 131.7(2C), 131.6, 130.9 (2C), 128.9 (2C), 124.9, 119.5 (2C).

HRMS (ESI-TOF) ***m/z*** Calcd for $C_{18}H_{13}S_2$ [M-BF_4^-]$^+$. 293.0459; Found 293.0460.

IR (cm^{-1}) 3085, 2939, 2835, 1596, 1477, 1445, 1256, 1171, 1110, 1055, 1031, 768, 757, 638.

Mp = 211 °C.

5-(Phenyl-d$_5$)-5H-thianthren-5-ium tetrafluoroborate ([$^2H_5$]1)

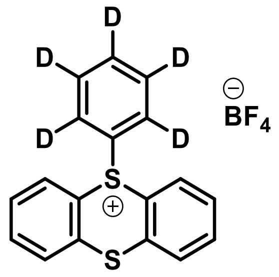

$C_{18}H_8D_5BF_4S_2$
MW: 385.08 g.mol^{-1}
Yield: 96%
Light-grey solid

The 5-(phenyl-*d5*)-5*H*-thianthren-5-ium tetrafluoroborate (**[$^2H_5$]1**) was prepared according to the general procedure GP1, using benzene-*d$_6$* (1.13 mL, 12.8 mmol), thianthrene-*S*-oxide TTO (5.95 g, 25.6 mmol), TFAA (5.30 mL, 38 mmol) and TMSOTf (4.6 mL, 25.6 mmol) in acetonitrile (64 mL, 0.2 M) for 3 hours at rt. After precipitation, the compound was a light-grey solid (4.72 g, 12.3 mmol, 96%).

^{1}H NMR (400 MHz, CD_3CN): δ 8.39-8.37 (m, 2H), 7.98-7.96 (m, 2H), 7.92-7.88 (td, *J* = 7.7, 1.4 Hz, 2H), 7.85-7.81 (m, 2H).

^{2}H NMR (61 MHz, $CHCl_3$) δ 8.11, 8.02, 7.79.

^{13}C NMR (100 MHz, CD_3CN): δ 137.6 (2C), 136.2 (2C), 136.1 (2C), 133.5 (m, 1C), 131.7(2C), 131.1 (m, 2C) 130.9 (2C), 128.6 (m, 2C), 124.7, 119.5 (2C).

^{19}F NMR (376 MHz, CD_3CN): -150.3 (m), -150.4 (m), presence of traces of OTf counter ion.

HRMS (ESI-TOF) ***m/z*** Calcd for $C_{18}H_8D_5S_2$ [M-BF4$^-$]$^+$. 298.0772; Found 298.0773.

IR (cm^{-1}): 3082, 2939, 2836, 1596, 1477, 1446, 1255, 1109, 1005, 1031, 768, 638, 518, 467.

Mp = 211 °C.

5-(Phenyl-$^{13}C_6$)-5H-thianthren-5-ium tetrafluoroborate ([$^{13}C_6$]1)

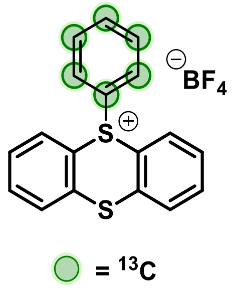

$C_{12}{}^{13}C_6H_{13}BF_4S_2$
MW: 386.07 g.mol^{-1}
Yield: 86%
Light-grey solid

The 5-(phenyl-$^{13}C_6$)-5*H*-thianthren-5-ium tetrafluoroborate (**[^{13}C$_6$]1**) was prepared according to the general procedure GP1, using [$^{13}C_6$]benzene (1.14 mL, 12.8 mmol), thianthrene-*S*-oxide TTO (6.0 mg, 25.6 mmol), TFAA (5.30 mL, 38.0 mmol) and TMSOTf (4.60 mL, 25.6 mmol) in acetonitrile (64 mL, 0.2 M) for 3 hours at rt. After precipitation, the compound was a light-grey solid (4.20 g, 11.0 mmol, 86%).

^{1}H NMR (400 MHz, CD_3CN) δ 8.38 (d, *J* = 7.9 Hz, 2H), 7.97 (d, *J* = 7.9 Hz, 2H), 7.90 (t, *J* = 7.6 Hz, 2H), 7.83 (t, *J* = 7.6 Hz, 2H), 7.71-7.30 (m, 4H), 6.96-6.87 (m, 1H), presence of traces of ethyl acetate.

^{1}H NMR-{^{13}C}NMR (400 MHz, CD_3CN) δ 8.39 (d, *J* = 7.9 Hz, 2H), 7.95 (d, *J* = 7.9 Hz, 2H), 7.89 (t, *J* = 7.6 Hz, 2H), 7.82 (t, *J* = 7.6 Hz, 2H), 7.60 (t, *J* = 7.3 Hz, 1H), 7.47 (t, *J* = 7.6 Hz, 2H), 7.11 (d, *J* = 7.7 Hz, 2H), presence of traces of ethyl acetate.

^{13}C NMR (100 MHz, CD_3CN) δ 137.7 (2C), 136.2 (2C), 136.1 (2C), 134.7-133.5 (m, 1C), 132.2-130.94 (m, 6C), 129.6-128.3 (m, 2C), 125.5-124.2 (m, 1C), 119.5 (2C).

^{19}F NMR (376 MHz, CD_3CN): -150.3 (m), -150.4 (m), presence of traces of OTf counter ion.

HRMS (ESI-TOF) ***m/z*** Calcd for $^{13}C_6C_{12}H_{13}S_2$ [M-BF4$^-$]$^+$. 299.0663; Found 299.0661.

IR (cm^{-1}) 3081, 2940, 2836, 1597, 1477, 1446, 1255, 1109, 1054, 1030, 767, 750, 637, 528, 467.

Mp = 211 °C.

4-Methoxy-1,1'-biphenyl (3)

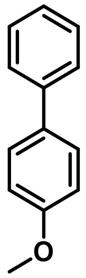

$C_{13}H_{12}O$
MW: 184.09 g.mol^{-1}
Yield: 91%
White solid

The 4-methoxy-1,1'-biphenyl (**3**) was prepared accordingly to the general procedure GP2, starting from **1** (114.0 mg, 0.30 mmol) and (4-methoxyphenyl)boronic acid (68.4 mg, 0.30 mmol). The product was obtained upon purification by column chromatography on silica gel (*n*-heptane/EtOAc (100:0 to 90:10)) as a white solid (50.3 mg, 91%).

^{1}H NMR (400 MHz, $CDCl_3$): δ 7.61-7.55 (m, 4H), 7.45-7.43 (m, 2H), 7.36-7.31 (m, 1H), 7.03-6.99 (m, 2H), 3.88 (s, 3H), presence of traces of dichloromethane.

^{13}C NMR (100 MHz, $CDCl_3$): δ 159.3, 141.0, 134.0, 128.9 (2C), 128.3 (2C), 126.9 (2C), 126.8, 114.3 (2C), 55.5.

HRMS (ASAP-TOF) *m/z* Calcd for $C_{13}H_{13}O$ [M+H]$^+$ 185.0966; Found 185.0968.

IR (cm^{-1}): 3034, 3001, 2959, 2836, 1607, 1583, 1522, 1489, 1464, 1450, 1441, 1407, 1289, 1270, 1251, 1201, 1184, 1119, 1037, 833, 761, 738, 691.

Mp = 87 °C.

4-Methoxy-1,1'-biphenyl-2',3',4',5',6'-d5 ([$^2H_5$]3)

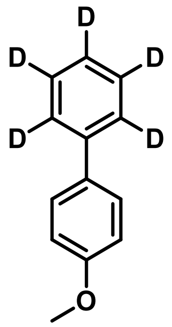

$C_{13}H_7D_5O$
MW: 189.12 g.mol^{-1}
Yield: 83%
White solid

The 4-methoxy-1,1'-biphenyl-2',3',4',5',6'-*d5* (**[$^2H_5$]3**) was prepared according to the general procedure GP2, using **[$^2H_5$]1** (115.5 mg, 0.30 mmol) and (4-methoxyphenyl)boronic acid (68.4 mg, 0.30 mmol),. The product was obtained upon purification by column chromatography on silica gel (*n*-heptane/EtOAc (100:0 to 98:2)) as a white solid (42.4 mg, 83%).

^{1}H NMR (400 MHz, $CDCl_3$) δ 7.59-7.55 (m, 2H), 7.03-7.00 (m, 2H), 3.88 (s, 3H).

^{2}H NMR-{^{1}H}NMR (61 MHz, $CHCl_3$) δ 7.62, 7.48, 7.37.

^{13}C NMR (100 MHz, $CDCl_3$) δ 159.3, 140.8, 134.0, 128.3 (m, 2C), 127.9 (2C), 126.4 (m, 2C), 126.3 (m, 1C), 114.3 (2C), 55.5.

HRMS (ASAP-TOF) *m/z* Calcd for $C_{13}H_8D_5O$ $[M+H]^+$ 190.1280; Found 190.1283.

IR (cm^{-1}) 3034, 2958, 2837, 2273, 1607, 1517, 1464, 1441, 1383, 1288, 1264, 1252, 1189, 1122, 1037, 840, 818, 768, 738, 640.

Mp = 87 °C.

1-Methoxy-4-(phenyl-$^{13}C_6$)benzene ([$^{13}C_6$]3)

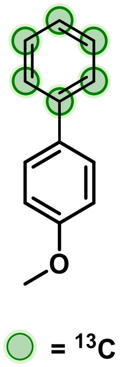

$C_7{}^{13}C_6H_{12}O$
MW: 190.12 g.mol^{-1}
Yield: 61%
White solid

The 1-methoxy-4-(phenyl-^{13}*C₆*)benzene (**[^{13}C$_6$]3**) was prepared according to the general procedure GP2, using **[^{13}C$_6$]1** (115.8 mg, 0.30 mmol) and (4-methoxyphenyl)boronic acid (68.4 mg, 0.30 mmol)The product was obtained upon purification by column chromatography on silica gel (*n*-heptane/EtOAc (100:0)) product as a white solid (38.5 mg, 61%).

^{1}H NMR (400 MHz, CDCl$_3$): δ 7.80-7.59 (m, 2H), 7.57-7.54 (m, 2H), 7.53-7.11 (m, 3H), 7.01-6.97 (m, 2H), 3.87 (s, 3H).

^{1}H NMR-{^{13}C}NMR (400 MHz, CDCl$_3$) δ 7.55 (m, 4H), 7.42 (t, *J* = 7.7 Hz, 2H), 7.31 (t, *J* = 7.5 Hz, 1H), 6.99 (d, *J* = 8.7 Hz, 2H), 3.86 (s, 3H).

^{13}C NMR (100 MHz, CDCl$_3$): δ 159.3, 141.5-133.9 (m, 1^{12}C, 1^{13}C, 2C),129.5-128.3 (m, 3^{12}C, 5^{13}C, 6C), 114.3, 114.1, 55.5.

HRMS (ASAP-TOF) *m/z* Calcd for $^{13}C_6C_7H_{13}O[M+H]^+$ 191.1170; Found 191.1166.

IR (cm^{-1}): 3055, 3001, 2958, 2836, 1607, 1518, 1463, 1440, 1400, 1280, 1247, 1198, 1184, 1116, 1037, 832, 751, 713, 676.

Mp = 87 °C.

3,4-Dimethoxy-1,1'-biphenyl-2',3',4',5',6'-d5 ([$^2H_5$]4)

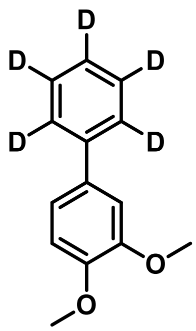

$C_{14}H_9D_5O_2$
MW: 219.13 g.mol^{-1}
Yield: 69 %
White solid

The 3,4-dimethoxy-1,1'-biphenyl-2',3',4',5',6'-d_5 (**[$^2H_5$]4)** was prepared according to the general procedure GP2, using **[$^2H_5$]1** (115.0 mg, 0.30 mmol) and (3,4-dimethoxyphenyl)boronic acid (54.6 mg, 0.30 mmol) The product was obtained upon purification by column chromatography on silica gel (*n*-heptane/EtOAc (80:20)) as a white solid (45.5 mg, 69%).

^{1}H NMR (400 MHz, CDCl$_3$) δ 7.16 (d, *J* = 8.3 Hz, 1H), 7.13 (s, 1H), 6.96 (d, *J* = 8.3 Hz, 1H), 3.96 (s, 3H), 3.93 (s, 3H).

^{2}H NMR-{^{1}H}NMR (61 MHz, CHCl$_3$) δ 7.60, 7.47, 7.38.

^{13}C NMR (100 MHz, CDCl$_3$) δ 149.2, 148.7, 140.9, 134.3, 128.3 (m, 2C), 126.5 (m, 2C), 126.4 (m, 1C), 119.4, 111.5, 110.5, 56.1, 56.0.

HRMS (ASAP-TOF) *m/z* Calcd for $C_{14}H_{10}D_5O_2$ [M+H]$^+$ 220.1386; Found 220.1385.

IR (cm^{-1}): 2969, 2283, 1517, 1464, 1415, 1382, 1260, 1243, 1170, 1143, 1027, 8103, 760.

Mp = 70 °C.

1,2-Dimethoxy-4-(phenyl-$^{13}C_6$)benzene ([$^{13}C_6$]4)

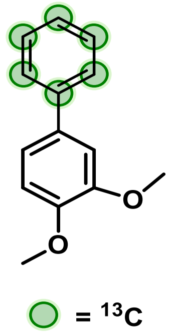

$C_8{}^{13}C_6H_{14}O_2$
MW: 220.12 g.mol^{-1}
Yield: 69 %
Black solid

The 1,2-dimethoxy-4-(phenyl-$^{13}C_6$)benzene (**III-[$^{13}C_6$]XX**) was prepared according to the general procedure GP2, using **[$^{13}C_6$]1** (1 158 mg, 3.0 mmol) and (3,4-dimethoxyphenyl)boronic acid (546.2 mg, 3.0 mmol). The product was obtained upon purification by Then, the crude was purified by column chromatography on silica gel (*n*-heptane/EtOAc (99:1)) affording the product as a black solid (455.3 mg, 69%).

^{1}H NMR-{^{13}C}NMR (400 MHz, CDCl$_3$) δ 7.60 (d, *J* = 7.8 Hz, 1H), 7.46 (t, *J* = 7.4 Hz, 1H), 7.35 (t, *J* = 7.4 Hz, 1H), 7.21 – 7.14 (m, 1H), 6.98 (d, *J* = 8.2 Hz, 1H), 3.99 (s, 1H), 3.96 (s, 1H).

^{13}C NMR (101 MHz, CDCl$_3$) δ 149.23, 149.18 -140.48 (m, ^{12}C and ^{13}C, 2C), 129.49 - 126.15 (m, 2x $^{13}C_{ortho}$ and 2x $^{13}C_{meta}$, 1x $^{13}C_{par,}$ 5C) 119.48, 111.57, 110.51, 56.04, 55.99.

HRMS (ESI-TOF) m/z Calcd for $^{13}C_6C_8H_{15}O_2$ [M+H]$^+$ 221.1276; Found 221.1275.

IR (cm^{-1}): 3050, 3001, 2961, 2936, 2834, 1718, 1601, 1586, 1518, 1461, 1440, 1399 1330, 1294, 1251, 1171, 1141, 1025, 854, 817, 710, 689, 584.

Mp = 69 °C.

1-(*tert*-Butyl)-4-(phenyl-13C6)benzene ([$^{13}C_6$]5)

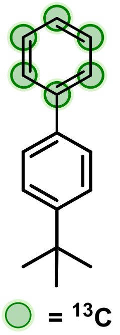

$C_{10}{}^{13}C_6H_{18}$
MW: 216.16 g.mol^{-1}
Yield: 64 %
White solid

The 1-(tert-butyl)-4-(phenyl-13C6)benzene (**[$^{13}C_6$]5**) was prepared according to the general procedure GP2, using **[$^{13}C_6$]1** (115.8 mg, 0.30 mmol) and (4-(*tert*-butyl)phenyl)boronic acid (53.4 mg, 0.30 mmol). To facilitate the separation between thianthrene (by-product (1.0 equiv.)) and the desired product, $Fe(NO_3).9H_2O$ (150 mg) was added to the solvent-free crude reaction mixture and was dissolved in DCM. After a few seconds in a ultrasonic bath, TFA (100 µL) was added to the mixture. After stirring during 5 min at rt, the full oxidation of the thianthrene was controlled by TLC, and the crude reaction mixture was purified by column chromatography on silica gel (*n*-heptane 100%) affording the product as a white solid (41.8 mg, 64%).

^{1}H NMR (400 MHz, $CDCl_3$) δ 7.91-7.75 (m, 1H), 7.74-7.61 (m, 1H), 7.61-7.56 (m, 2H), 7.55-7.05 (m, 5H), 1.41 (s, 9H).

^{1}H NMR-{^{13}C}NMR (400 MHz, $CDCl_3$) δ 7.63 (d, J = 7.8 Hz, 2H), 7.58 (d, J = 8.3 Hz, 2H), 7.51 (d, J = 8.3 Hz, 2H), 7.46 (t, J = 7.6 Hz, 2H), 7.36 (t, J = 7.5 Hz, 1H).

^{13}C NMR (101 MHz, $CDCl_3$) δ 150.3, 141.7-140.5 (m, 1x ^{12}C, 1x ^{13}C, 2C), 129.5-125.8 (m, 5x ^{12}C, 5x ^{13}C, 10C), 34.6, 31.5 (2C).

HRMS (ASAP-TOF) m/z Calcd for $^{13}C_6C_{10}H_{18}$ $[M]^+$ 216.1613; Found 216.1612.

IR (cm^{-1}): 3050, 2961, 2902, 2866, 1912, 1548, 1522, 1475, 1458, 1393, 1362, 1269, 1201, 1112, 1055, 1027, 984, 901, 835, 757, 728, 682, 655, 569, 508, 417, 407.

Mp = 52 °C.

4-Chloro-1,1'-biphenyl-2',3',4',5',6'-d5 ([$^2H_5$]6)

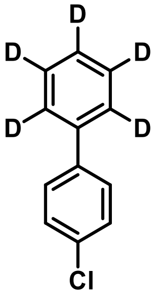

$C_{12}H_4D_5Cl$
MW: 193.07 g.mol^{-1}
Yield: 59 %
White solid

The 4-chloro-1,1'-biphenyl-2',3',4',5',6'-*d*5 (**[$^2H_5$]6**) was prepared according to the general procedure GP2, using **[$^2H_5$]1** (115.0 mg, 0.30 mmol) and (4-chlorophenyl)boronic acid (34.3 mg, 0.30 mmol). To facilitate the separation between thianthrene (by-product (1.0 equiv.)) and the product, $Fe(NO_3).9H_2O$ (150 mg) was added to the solvent-free crude reaction mixture and was dissolved in DCM. After a few seconds in an ultrasonic bath, TFA (100 µL) was added to the mixture. After stirring during 5 min at rt, the full oxidation of the thianthrene was controlled by TLC, then, the crude reaction mixture was purified by column chromatography on silica gel (*n*-heptane 100%) affording the product as a yellowish solid (34.3 mg, 59%).

^{1}H NMR (400 MHz, $CDCl_3$) δ 7.55-7.52 (m, 2H), 7.44-7.41 (m, 2H).

^{2}H NMR-{^{1}H}NMR (61 MHz, $CHCl_3$) δ 7.61, 7.49, 7.42.

^{13}C NMR (100 MHz, $CDCl_3$) δ 139.9, 139.7, 133.5, 129.0 (2C), 128.5 (m, 2C), 128.5 (2C), 127.2 (m, 1C), 126.7 (m, 2C).

HRMS (ASAP-TOF) *m/z* Calcd for $C_{12}H_5D_5Cl$ $[M+H]^+$ 194.0785; Found 194.0782.

IR (cm^{-1}): 2957, 2284, 1592, 1499, 1381, 1096, 840, 818, 764, 639, 550, 442.

Mp = 79 °C.

[1,1'-Biphenyl]-4-carbonitrile-2',3',4',5',6'-d$_5$ ([$^2H_5$]7)

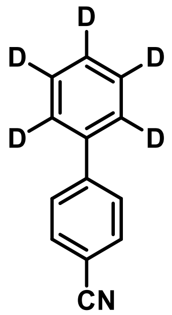

$C_{13}H_4D_5N$
MW: 184.10 g.mol^{-1}
Yield: 64%
Yellow oil

The [1,1'-biphenyl]-4-carbonitrile-2',3',4',5',6'-*d*$_5$ (**[^{2}H$_5$]7**) was prepared according to the general procedure GP9, using **[^{2}H$_5$]1** (115.0 mg, 0.30 mmol) and (4-cyanophenyl)boronic acid (44.1 mg, 0.30 mmol). To facilitate the separation between thianthrene (by-product (1.0 equiv.)) and the product, $Fe(NO_3).9H_2O$ (150 mg) was added to the solvent-free crude reaction mixture which was dissolved in DCM. After a few seconds in a ultrasonic bath, TFA (100 μL) was added to the mixture. After stirring during 5 min at rt, the full oxidation of the thianthrene was controlled by TLC. Then, the crude reaction mixture was purified by column chromatography on silica gel (*n*-heptane/EtOAc (93:7) affording the product as a yellow oil (35.3 mg, 64%).

^{1}H NMR (400 MHz, $CDCl_3$) δ 7.74-7.67 (m, 4H).

^{2}H NMR-{^{1}H}NMR (61 MHz, $CHCl_3$) δ 7.64, 7.53.

^{13}C NMR (100 MHz, $CDCl_3$) δ 145.7, 139.1, 132.7 (2C), 128.7 (m, 2C), 128.3 (m, 1C), 127.8 (2C), 126.9 (m, 2C), 119.1, 111.0.

HRMS (ASAP-TOF) *m/z* Calcd for $C_{13}H_5D_5N$ [M+H]$^+$ 185.1127; Found 185.1128.

IR (cm^{-1}): 2226, 1606, 853.

4-(Phenyl-$^{13}C_6$)benzoic acid ([$^{13}C_6$]8)

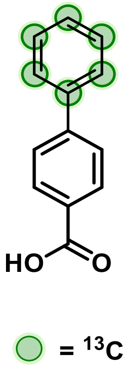

$C_7{}^{13}C_6H_{10}O_2$
MW: 204.09 g.mol^{-1}
Yield: 56%
yellowish solid

The 4-(phenyl-$^{13}C_6$)benzoic acid (**[^{13}C$_6$]8**) was prepared according to the general procedure GP2, using **[^{13}C$_6$]1** (115.8 mg, 0.30 mmol) and (4-methoxyphenyl)boronic acid (49.5 mg, 0.30 mmol. The product was obtained upon purification by column chromatography on silica gel (*n*-heptane/EtOAc (100:0 to 80:20)) affording the product as a yellowish solid (34.43 mg, 56%).

^{1}H NMR (400 MHz, MeOD): δ 8.08 (d, *J* = 8.3 Hz, 2H), 7.89–7.84 (m, 1H), 7.72-7.69 (m, 2H), 7.66 to 7.17 (m, 4H), 1H missing from carboxylic acid functional group.

^{1}H NMR-{^{13}C}NMR (400 MHz, DMF-*d*$_7$) δ 8.14 (d, *J* = 8.2 Hz, 2H), 7.84 (d, *J* = 8.1 Hz, 2H) 7.79 (d, *J* = 7.8 Hz, 2H), 7.53 (t, *J* = 7.5 Hz, 2H), 7.45 (t, *J* = 7.3 Hz, 1H).

^{13}C NMR (101 MHz, MeOD) δ 170.4, 141.9-140.7 (m, 1^{12}C, 1^{13}C, 2C), 132.1-126.8 (m, 5x ^{12}C, 5x ^{13}C, 10 C).

HRMS (ASAP-TOF) *m/z* Calcd for $^{13}C_6C_7H_{11}O_2$ [M+H]$^+$ 205.0963; Found 205.0958.

IR (cm^{-1}): 2987, 2836, 1680, 1608, 1394, 1295, 1066, 862, 742.

Mp = 216 °C.

Phenyl(4-(phenyl-$^{13}C_6$)phenyl)methanone ([$^{13}C_6$]9)

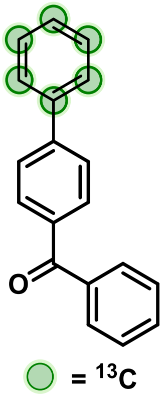

$C_{13}{}^{13}C_6H_{14}O$
MW: 264.12 g.mol^{-1}
Yield: 74 %
White solid

The phenyl(4-(phenyl-$^{13}C_6$)phenyl)methanone (**[$^{13}C_6$]9)** was prepared according to the general procedure GP2, using **[$^{13}C_6$]1** (115.8 mg, 0.30 mmol) and (4-benzoylphenyl)boronic acid (67.8 mg, 0.30 mmol)Subsequently, the solvents of the crude reaction mixture were removed under reduced pressure. To facilitate the separation between thianthrene (by-product (1.0 equiv.)) and the product, $Fe(NO_3).9H_2O$ (150 mg) was added to the solvent free crude reaction mixture which was dissolved in DCM. After a few seconds in a ultrasonic bath, TFA (100 µL) was added to the mixture. After stirring during 5 min at rt, the full oxidation of the thianthrene was controlled by TLC, then, the crude reaction mixture was purified by column chromatography on silica gel (*n*-heptane 100%) affording the product as a white powder (58.6 mg, 74%).

^{1}H NMR (400 MHz, CDCl$_3$): δ 7.91 (m, 2H), 7.88-7.79 (m, 3H), 7.71 (m, 2H), 7.59-7.69 (m, 2H), 7.54-7.43 (m, 3H), 7.38-7.13 (m, 2H).

^{1}H NMR-{^{13}C}NMR (400 MHz, CDCl$_3$) δ 7.91 (d, J = 8.3 Hz, 2H), 7.85 (d, J = 8.2 Hz, 2H), 7.71 (d, J = 8.3 Hz, 2H), 7.6 (d, J = 7.9 Hz, 2H), 7.61 (t, J = 7.4 Hz, 1H), 7.53 -7.47 (m, 4H), 7.41 (t, J = 7.4 Hz, 1H).

^{13}C NMR (101 MHz, CDCl$_3$) δ 196.3, 140.5-126.6 (m, 12× ^{12}C, 6× ^{13}C, 18C).

HRMS (ASAP-TOF) ***m/z*** Calcd for $^{13}C_6C_{13}H_{15}O$ [M+H]$^+$ 265.1327; Found 265.1324.

IR (cm^{-1}): 3052, 1643, 1597, 1444, 1313, 1283, 922, 938, 850, 723, 692, 675, 622, 460.

2-Fluoro-1,1'-biphenyl-2',3',4',5',6'-d$_5$ ([$^2H_5$]10)

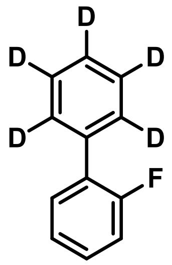

$C_{12}H_4D_5F$
MW: 177.10 g.mol^{-1}
Yield: 40 %
White solid

The 2-fluoro-1,1'-biphenyl-2',3',4',5',6'-*d*$_5$ (**[$^2H_5$]10**) was prepared according to the general procedure GP2, using **[$^2H_5$]1** (229 mg, 0.60 mmol) and phenylboronic acid (42.0 mg, 0.30 mmol). Subsequently, the solvents of the crude reaction mixture were removed under reduced pressure. To facilitate the separation between thianthrene (by-product (1.0 equiv.)) and the product, $Fe(NO_3).9H_2O$ (150 mg) was added to the solvent-free crude which was then dissolved in DCM. After a few seconds in a ultrasonic bath, TFA (100 μL) was added to the mixture. After stirring during 5 min at rt, the full oxidation of the thianthrene was controlled by TLC, then, the crude reaction mixture was purified by column chromatography on silica gel (*n*-heptane 100%) affording the product as a white solid (21.2 mg, 40%).

^{1}H NMR (400 MHz, CDCl$_3$) δ 7.46 (td, J = 7.7, 1.8 Hz, 1H), 7.33 (dddd, J = 8.1, 6.9, 5.0, 1.8 Hz, 1H). 7.22 (td, J = 7.5, 1.3 Hz, 1H), 7.17 (ddd, 10.7, 8.1, 1.1 Hz, 1H), presence of traces of dichloromethane.

^{2}H NMR-{^{1}H}NMR (61 MHz, CHCl$_3$) δ 7.61, 7.50, 7.42.

^{13}C NMR (100 MHz, CDCl$_3$) δ 159.6 (d, J = 247.7 Hz, 1C), 135.8, 130.9 (d, J = 3.5 Hz, 1C), 129.2, 129.1 (d, J = 8.2 Hz, 1C), 128.7 (m, 2C), 128.1 (m, 2C), 127.3 (m, 1C), 124.5 (d, J = 3.7 Hz, 1C), 116.2 (d, J = 22.8 Hz, 1C).

HRMS (ASAP-TOF) *m/z* Calcd for $C_{12}H_5D_5F$ $[M]^{\cdot+}$ 177.1002; Found 177.1004.

IR (cm^{-1}) 2924, 2284, 1615, 1491, 1446, 1385, 1324, 1232, 1210, 1200, 1105, 1037, 810, 757.

Mp = 77 °C.

2-(Phenyl-13C6)-1,1'-biphenyl ([$^{13}C_6$]11)

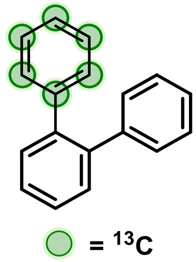

$C_{12}{}^{13}C_6H_{14}$
MW: 236.13 g.mol^{-1}
Yield: 55%
Yellowish oil

The 2-(phenyl-13C6)-1,1'-biphenyl (**[$^{13}C_6$]11**) was prepared according to the general procedure GP2, using **[$^{13}C_6$]1** (115.8 mg, 0.30 mmol), [1,1'-biphenyl]-2-ylboronic acid (59.4 mg, 0.30 mmol) Subsequently, the solvents of the crude reaction mixture were removed under reduced pressure. To facilitate the separation between thianthrene (by-product (1.0 equiv.)) and the product, $Fe(NO_3).9H_2O$ (150 mg) was added to the solvent-free crude reaction mixture and was dissolved in DCM. After a few seconds in a ultrasonic bath, TFA (100 µL) was added to the mixture. After stirring during 5 min at rt, the full oxidation of the thianthrene was controlled by TLC, then, the crude was purified by column chromatography on silica gel (*n*-heptane 100%) affording the product as a yellowish oil (39.2 mg, 55%).

^{1}H NMR (400 MHz, $CDCl_3$): δ 7.48–7.42 (m, 4H), 7.40-7.34 (m, 1H), 7.26-7.14 (m, 5H), 7.06-6.94 (m, 2H), presence of traces of residual grease.

^{1}H NMR-{^{13}C}NMR (400 MHz, $CDCl_3$) δ 7.49-7.42 (m, 4H), 7.25-7.20 (m, 6H), 7.20-7.14 (m, 4H), presence of traces of residual grease.

^{13}C NMR (101 MHz, $CDCl_3$) δ 142.1-141.0 (m, 1^{12}C, 1^{13}C, 2C), 130.6-125.8 (m, 11× ^{12}C, 5× ^{13}C, 16C).

HRMS (ASAP-TOF) m/z Calcd for $^{13}C_6C_{12}H_{14}$ [M]$^+$ 236.1299; Found 236.1296.

IR (cm^{-1}): 3051, 2966, 1596, 1546, 1479, 1455, 1438, 1410, 1273, 1073, 1054, 1007, 982, 903, 767, 744, 685, 699, 616, 551, 519, 493, 417.

1,1'-Biphenyl-2,3,4,5,6-d5 ([$^2H_5$]12)

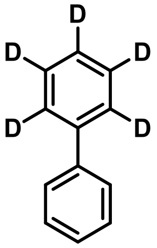

$C_{12}H_5D_5$
MW: 159.24 g.mol^{-1}
Yield: 48%
White solid

The 1,1'-biphenyl-2,3,4,5,6-d_5 (**[$^2H_5$]12**) was prepared according to the general procedure GP2, using **[$^2H_5$]1** (115.0 mg, 0.30 mmol) and phenylboronic acid (36.6 mg, 0.30 mmol). Subsequently, the solvents of the crude reaction mixture were removed under reduced pressure. To facilitate the separation between thianthrene (by-product (1.0 equiv.)) and the product, $Fe(NO_3).9H_2O$ (150 mg) was added to the solvent-free crude reaction mixture and was dissolved in DCM. After a few seconds in a ultrasonic bath, TFA (100 μL) was added to the mixture. After stirring during 5 min at rt, the full oxidation of the thianthrene was controlled by TLC, then, the crude reaction mixture was purified by column chromatography on silica gel (*n*-heptane 100%) affording the product as a white solid (23.0 mg, 48%).

^{1}H NMR (400 MHz, $CDCl_3$) δ 7.64-7.61 (m, 2H), 7.49-7.45 (m, 2H), 7.40-7.35 (m, 1H), presence of traces of dichloromethane.

^{2}H NMR-{^{1}H}NMR (61 MHz, $CHCl_3$) δ 7.65, 7.49, 7.40.

^{13}C NMR (100 MHz, $CDCl_3$) δ 141.3, 141.2, 128.9 (2C), 128.4 (m, 2C), 127.4, 127.3 (2C), 126.9 (m, 3C).

HRMS (ASAP-TOF) *m/z* Calcd for $C_{12}H_6D_5O$ $[M+H]^+$ 160.1175; Found 160.1171.

IR (cm^{-1}): 2924, 2284, 1615, 1491, 1446, 1385, 1324, 1232, 1210, 1200, 1105, 1037, 810, 757.

Mp = 77 °C.

1-(Phenyl-13C6)dibenzo[*b,d*]furan ([$^{13}C_6$]13)

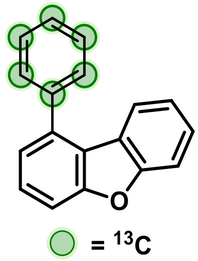

$C_{12}{}^{13}C_6H_{12}O$
MW: 250.11 g.mol^{-1}
Yield: 72%
Transparent oil

The 1-(phenyl-13C6)dibenzo[*b,d*]furan (**[$^{13}C_6$]13**) was prepared according to the general procedure GP2, using **[$^{13}C_6$]1** (115.8 mg, 0.30 mmol) and dibenzo[b,d]furan-1-ylboronic acid (63.6 mg, 0.30 mmol). Subsequently, the solvents of the crude reaction mixture were removed under reduced pressure. To facilitate the separation between thianthrene (by-product (1.0 equiv.)) and the product, $Fe(NO_3).9H_2O$ (150 mg) was added to the solvent-free crude reaction mixture which was then dissolved in DCM. After a few seconds in a ultrasonic bath, TFA (100 µL) was added to the mixture. After stirring during 5 min at rt, the full oxidation of the thianthrene was controlled by TLC, then, the crude was purified by column chromatography on silica gel (*n*-heptane 100%) affording the product as a transparent oil (54.3 mg, 72%).

^{1}H NMR (400 MHz, CDCl$_3$): δ 7.95–7.48 (m, 3H), 7.60 (dd, *J* = 8.2, 0.8 Hz, 2H), 7.54 (t, *J* = 7.7 Hz, 2H), 7.46-7.39 (m, 2H), 7.39-7.31 (m, 1H), 7.31-7.26 m, 1H), 7.18-7.12 (m, 1H), presence of traces of impurities.

^{1}H NMR-{^{13}C}NMR (400 MHz, CDCl$_3$) δ 7.66 (d, *J* = 7.2 Hz, 2H), 7.62-7.48 (m, 7H), 7.43 (t, *J* = 7.4 Hz, 1H), 7.28 (d, *J* = 7.3 Hz, 1H), 7.15 (t, *J* = 7.5 Hz, 1H), presence of traces of impurities.

^{13}C NMR (101 MHz, CDC$_3$) δ 156.5, 156.4, 156.4, 141.2-137.6 (m, 1× ^{12}C, 1× ^{13}C, 2C), 130.1-121.8 (m, 6× ^{12}C, 5× ^{13}C, 11C), 111.5, 110.5.

HRMS (ASAP-TOF) m/z Calcd for $^{13}C_6C_{12}H_{12}O$ [M]$^+$ 250.1092; Found 250.1088.

IR (cm^{-1}): 3049, 2921, 1579, 1545, 1479, 1455, 1447, 1433, 1407, 1233, 1192, 1109, 1052, 1007, 901, 846, 767, 744, 725, 699, 686, 616, 602, 552, 519. 410.

4-(Phenyl-$^{13}C_6$)dibenzo[*b,d*]furan ([$^{13}C_6$]14)

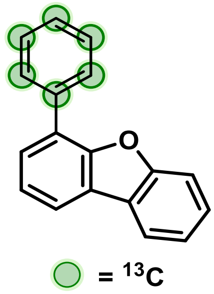

$C_{12}{}^{13}C_6H_{12}O$
MW: 250.11 g.mol^{-1}
Yield: 65 %
White oil

The 4-(phenyl-^{13}*C*$_6$)dibenzo[*b,d*]furan (**[$^{13}C_6$]14**) was prepared accordingly to general procedure GP2, using **[$^{13}C_6$]1** (115.8 mg, 0.30 mmol) and dibenzo[b,d]furan-4-ylboronic acid (63.3 mg, 0.30 mmol). Subsequently, the solvents of the crude reaction mixture were removed under reduced pressure. To facilitate the separation between thianthrene (by-product (1.0 equiv.)) and the product, $Fe(NO_3).9H_2O$ (150 mg) was added to the solvent-free crude which was then dissolved in DCM. After a few seconds in a ultrasonic bath, TFA (100 µL) was added to the mixture. After stirring during 5 min at rt, the full oxidation of the thianthrene was controlled by TLC, then, the crude was purified by column chromatography on silica gel (*n*-heptane 100%) affording the product as a white oil (48.9 mg, 65%).

^{1}H NMR (400 MHz, $CDCl_3$): δ 8.27-8.04 (m, 1H), 8.01 (ddd, *J* = 7.7, 1.3, 0.6 Hz, 1H), 7.96 (dd, *J* = 7.7, 1.0 Hz, 1H), 7.87-7.27 (m, 9H), presence of traces of impurities and dichloromethane.

^{1}H NMR-{^{13}C}NMR (400 MHz, $CDCl_3$) δ 8.01 (d, *J* = 7.7 Hz, 1H), 7.96 (dd, *J* = 6.9, 4.9 Hz, 3H), 7.63 (d, *J* = 7.8 Hz, 2H), 7.57 (t, *J* = 7.7 Hz, 2H), 7.52-7.43 (m, 3H), 7.39 (t, *J* = 7.4 Hz, 1H), presence of traces of impurities and dichloromethane.

^{13}C NMR (101 MHz, $CDCl_3$) δ 156.2, 153.4, 137.5-135.5 (m, 1x ^{12}C, 1x ^{13}C, 2C), 135.2-119.7 (m, 8x ^{12}C, 5x ^{13}C, 13C), 111.9.

HRMS (ASAP-TOF) m/z Calcd for $^{13}C_6C_{12}H_{12}O$ [M]$^+$ 250.1092; Found 250.1088.

IR (cm^{-1}): 3053, 1582, 1547, 1493, 1475, 1448, 1426, 1397, 1310, 1257, 1189, 1123, 1097, 1053, 1009, 989, 868, 799, 748, 730, 681, 606, 574, 483, 438.

Isopropyl 2-methyl-2-(4-(4-(phenyl-$^{13}C_6$)benzoyl)phenoxy)propanoate ([$^{13}C_6$]15)

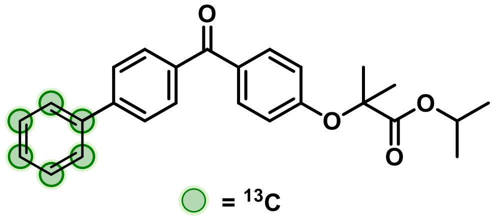

$C_{20}{}^{13}C_6H_{26}O_4$
MW: 408.20 g.mol^{-1}
Yield: 80 %
White solid

The isopropyl 2-methyl-2-(4-(4-(phenyl-$^{13}C_6$)benzoyl)phenoxy)propanoate ([$^{13}C_6$]**15)** was prepared according to the general procedure GP2, using **[$^{13}C_6$]1** (77.2 mg, 0.20 mmol) and isopropyl-2-methyl-2-(4-(4-(4,4,5,5-tetramethyl-1,3,2-dioxaborolan-2-yl)benzoyl)phenoxy)propanoate (90.4 mg, 0.20 mmol). The product was purified by column chromatography on silica gel (*n*-heptane/EtOAc (85:15)) (n-heptane 100%) and obtained as a white solid (65.2 mg, 80%).

1H NMR-{^{13}C}NMR (400 MHz, CDCl$_3$) δ 7.79 (dd, *J* = 15.8, 8.5 Hz, 4H), 7.66 (d, *J* = 8.2 Hz, 2H), 7.61 (d, *J* = 7.7 Hz, 2H), 7.44 (t, *J* = 7.5 Hz, 2H), 7.36 (t, *J* = 7.3 Hz, 1H), 6.85 (d, *J* = 8.7 Hz, 2H), 5.13 – 4.99 (m, 1H), 1.64 (s, 6H), 1.18 (d, *J* = 6.3 Hz, 6H).

^{13}C NMR (101 MHz, CDCl$_3$) δ 195.2, 173.2, 159.5, 140.7-139.5 (m, 1x^{13}C and 2x^{12}C, 3C), 132.0-126.6 (m, 5x ^{13}C and 7x ^{12}C, 12C), 117.2 (2C), 79.4, 69.3, 25.4 (2C), 21.5 (2C).

HRMS (ESI-TOF) m/z Calcd for $^{13}C_6C_{20}H_{27}O_4$ [M+H]$^+$ 409.2113; Found 409.2116.

IR (cm^{-1}): 2986, 1729, 1651, 1601, 1383, 1287, 1250, 1176, 1147,1101, 929, 857, 787, 757, 710, 692, 623, 409.

Mp = 126 °C.

Methyl 2-(4-(phenyl-$^{13}C_6$)phenyl)acetate ([$^{13}C_6$]16)

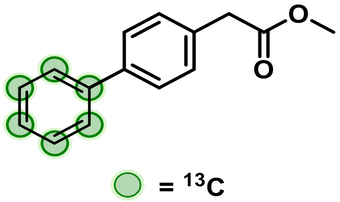

$C_9{}^{13}C_6H_{14}O_2$
MW: 232.12 g.mol^{-1}
Yield: 90 %
White oil

The isopropyl methyl 2-(4-(phenyl-13C_6)phenyl)acetate ([$^{13}C_6$]1**6)** was prepared according to the general procedure GP2, using **[$^{13}C_6$]1** (77.2 mg, 0.20 mmol) and the previously prepared methyl 2-(4-(4,4,5,5-tetramethyl-1,3,2-dioxaborolan-2-yl)phenyl)acetate (55.2 mg, 0.20 mmol). The product was obtained upon purification by column chromatography on silica gel (*n*-heptane/EtOAc (90:10)) as a white oil (41.7 mg, 90%).

^{1}H NMR (400 MHz, CDCl$_3$) δ 7.58 (dd, *J* = 17.4, 9.8 Hz, 4H), 7.43 (t, *J* = 7.6 Hz, 2H), 7.35 (t, *J* = 9.5 Hz, 3H), 3.72 (s, 3H), 3.68 (s, 2H), presence of traces of impurities.

^{13}C NMR (101 MHz, CDCl$_3$) δ 172.2, 141.5-140.2 (m, 1^{13}C and 1^{12}C, 2C), 133.1, 129.8-126.5 (m, 5^{13}C and 4^{12}C, 9C), 52.3, 40.9.

HRMS (ASAP-TOF) m/z Calcd for $^{13}C_6C_9H_{15}O_2$ [M]$^+$ 233.1276; Found 233.1273.

IR (cm^{-1}): 2952, 1737, 1515, 1458, 1435, 1258, 1159, 1018, 820, 745, 683¸ 491.

Ethyl 4-(8-(phenyl-13C6)-5,6-dihydro-11H-benzo[5,6]cyclohepta[1,2-b]pyridin-11-ylidene)piperidine-1-carboxylate ([$^{13}C_6$]17)

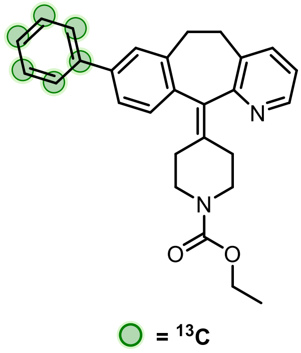

$C_{22}{}^{13}C_6H_{28}N_2O_2$
MW: 430.24 g.mol^{-1}
Yield: xx %
White solid

The ethyl 4-(8-(phenyl-13C6)-5,6-dihydro-11H-benzo[5,6]cyclohepta[1,2-b]pyridin-11-ylidene)piperidine-1-carboxylate **([$^{13}C_6$]17)** was prepared according to the general procedure GP2, using **[$^{13}C_6$]1** (114 mg, 0.30 mmol) and (11-(1-(ethoxycarbonyl)piperidin-4-ylidene)-6,11-dihydro-5H-benzo[5,6]cyclohepta[1,2-b]pyridin-8-yl)boronic acid (118 mg, 0.30 mmol). The product was obtained upon purification by column chromatography on silica gel (*n*-heptane/EtOAc) as a white solid (107 mg, 84%).

^{1}H NMR (400 MHz, Methanol-d$_4$) δ 8.29 (dd, J = 4.9, 1.6 Hz, 1H), 7.76 – 6.99 (m, 10H), 4.11 (q, J = 7.1 Hz, 2H), 3.82 – 3.68 (m, 2H), 3.54 – 3.34 (m, 2H), 3.23 – 3.07 (m, 2H), 3.01 – 2.78 (m, 2H), 2.47 – 2.32 (m, 3H), 2.23 – 2.11 (m, 1H), 1.23 (t, J = 7.1 Hz, 3H).

1H NMR-{^{13}C}NMR (400 MHz, Methanol-d$_4$) δ 8.29 (dd, J = 4.9, 1.6 Hz, 1H), 7.63 – 7.60 (m, 1H), 7.57 – 7.49 (m, 2H), 7.41 – 7.33 (m, 4H), 7.30 – 7.15 (m, 3H), 4.11 (q, J = 7.1 Hz, 2H), 3.81 – 3.67 (m, 2H), 3.49 – 3.33 (m, 2H), 3.24 – 3.11 (m, 2H), 2.93 – 2.78 (m, 2H), 2.43 – 2.28 (m, 3H), 2.23 – 2.12 (m, 1H), 1.23 (t, J = 7.1 Hz, 3H).

^{13}C NMR (101 MHz, CDCl$_3$) δ 158.6, 157.1, 146.7, 142.6–140.9 (m, 1^{13}C), 139.7, 139.3, 139.3, 138.6, 138.0, 136.2, 135.6, 130.8–126.8 (m, 5^{13}C), 125.6, 124.1, 119.4, 116.5, 62.8, 45.7, 45.6, 32.9, 32.2, 31.6, 31.5, 14.9.

HRMS (ASAP-TOF) m/z calcd for $^{13}C_6C_{22}H_{29}N_2O_2$ [M+H]$^+$ 431.2433; found 431.2433.

IR (cm^{-1}): 2919, 2851, 1691, 1638, 1602, 1579, 1473, 1433, 1384, 1355, 1320, 1302, 1274, 1170, 1117, 1093, 1060, 995, 855, 783, 764.

Methyl 2-(5-methoxy-2-methyl-1-(4-(phenyl-13C6)benzoyl)-1H-indol-3-yl)acetate ([$^{13}C_6$]18)

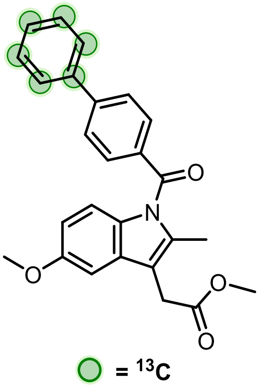

$C_{20}{}^{13}C_6H_{23}NO_4$
MW: 419.43 g.mol^{-1}
Yield: xx %
Brown oil

The methyl 2-(5 -methoxy-2-methyl-1-(4-(phenyl-13C6)benzoyl)-1H-indol-3-yl)acetate **([$^{13}C_6$]18)** was prepared according to the general procedure GP2, using **[$^{13}C_6$]1** (114 mg, 0.30 mmol) and (4-(5-methoxy-3-(2-methoxy-2-oxoethyl)-2-methyl-1H-indole-1-carbonyl)phenyl)boronic acid (114 mg, 0.30 mmol). The product was obtained upon purification by column chromatography on silica gel (*n*-heptane/EtOAc) as a brown oil (40 mg, 32%).

^{1}H NMR (400 MHz, Chloroform-d) δ δ 7.97 – 7.29 (m, 9H), 6.99 – 6.93 (m, 2H), 6.68 (dd, J = 9.0, 2.6 Hz, 1H), 3.85 (s, 3H), 3.72 (s, 3H), 3.70 (s, 2H), 2.43 (s, 3H), presence of impurities.

1H NMR-{^{13}C}NMR (400 MHz, Chloroform-d) δ 7.84 – 7.76 (m, 2H), 7.74 – 7.69 (m, 2H), 7.69 – 7.63 (m, 2H), 7.53 – 7.38 (m, 3H), 6.99 – 6.94 (m, 2H), 6.68 (dd, J = 9.0, 2.5 Hz, 1H), 3.85 (s, 3H), 3.72 (s, 3H), 3.70 (s, 2H), 2.43 (s, 2H), presence of impurities.

^{13}C NMR (101 MHz, $CDCl_3$) δ 171.6, 169.3, 156.0, 139.7 (m, 1^{13}C), 136.2, 134.3, 131.1, 130.7, 130.5 – 125.5 (m, 5^{13}C and 4^{12}C, 4C), 115.0, 112.2, 111.6, 101.3, 55.9, 52.3, 30.3, 13.5.

HRMS (ASAP-TOF) m/z calcd for $^{13}C_6C_{20}H_{24}NO_4$ $[M+H]^+$ 420.1909; found 420.1909.

IR (cm^{-1}): X (cm-1) 2950, 1735, 1679, 1604, 1477, 1456, 1435, 1396, 1355, 1314, 1259, 1223, 1166, 1144, 1068, 1036, 984, 925, 838, 803, 772, 738, 706, 682, 604, 555, 433, 405.

Unsuccessful substrates

Corresponding boronic acids did not provide the desired products [$^{13}C_6$]I, [$^{13}C_6$]II, [$^{13}C_6$]III, [$^{13}C_6$]VI, [$^{13}C_6$]V under the standard reactions conditions.

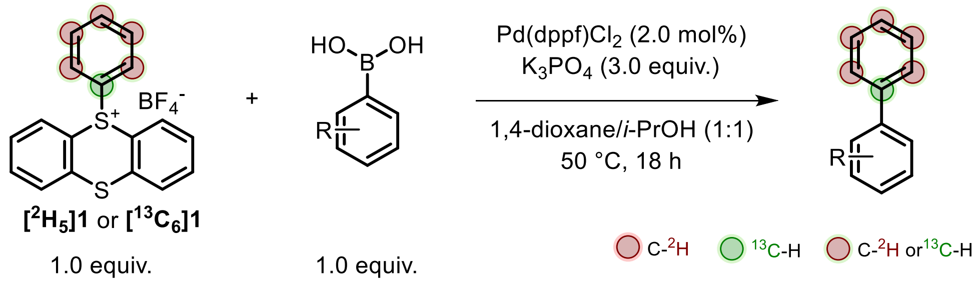

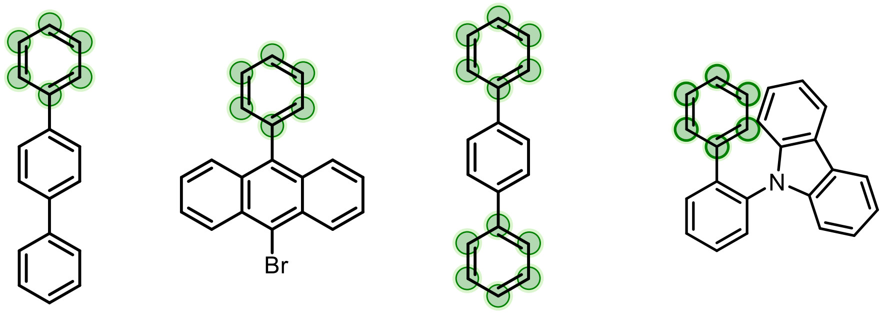

[$^{13}C_6$]I
Traces

[$^{13}C_6$]II
Traces

[$^{13}C_6$]III
Traces

[$^{13}C_6$]IV
No reaction

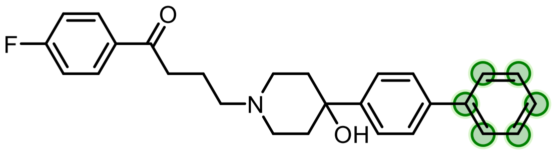

[$^{13}C_6$]V
from Haloperidol
Purification problem

Ethyl 2-((phenyl-D$_5$)thio)acetate ([^{2}H$_5$]19)

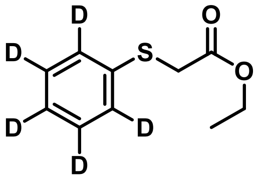

$C_4{}^{13}C_6H_{12}O_2S$
MW: 201.29 g.mol^{-1}
Yield: 85 %
White oil

The ethyl 2-((phenyl-D$_5$)thio)acetate (**[^{2}H$_5$]20)** was prepared according to the general procedure GP3, using **[^{13}C$_6$]1** (77.2 mg, 0.20 mmol) and Ethyl thioglycolate (22.2 μL, 0.2 mmol). The product was obtained upon purification by column chromatography on silica gel (*n*-heptane/EtOAc (90:10)) as a white oil (36.1 mg, 90%).

^{1}H NMR-{^{13}C}NMR (400 MHz, CDCl$_3$) δ 4.16 (q, J = 7.1 Hz, 1H), 3.63 (s, 1H), 1.21 (t, J = 7.1 Hz, 1H).

^{13}C NMR (101 MHz, CDCl$_3$) δ 169.7, 134.8, 129.8-126.5 (m, 5C), 61.6, 36.7, 14.1.

HRMS (ASAP-TOF) m/z Calcd for $C_{10}H_7D_5O_2S$ [M]$^+$ 201.0872; Found 201.0870.

^{2}H NMR-{^{1}H}NMR (61 MHz, CHCl$_3$) δ 7.42, 7.33, 7.26.

IR (cm^{-1}): 2981, 2936, 2276, 1731, 1547, 1464, 1444, 1406, 1366, 1341, 1268, 1130, 1096, 1028, 943, 895, 866, 840, 826, 751, 699, 664, 623, 545, 441.

Methyl 2-((phenyl-$^{13}C_6$)thio)benzoate ([$^{13}C_6$]20)

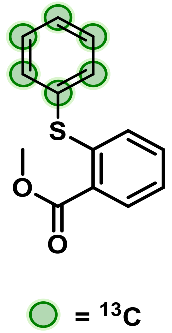

$C_8{}^{13}C_6H_{12}O_2S$
MW: 250.71 g.mol^{-1}
Yield: 87 %
Colorless oil

The methyl 2-((phenyl-$^{13}C_6$)thio)benzoate (**[$^{13}C_6$]22**) was prepared according to the general procedure GP3, using **[$^{13}C_6$]1** (77.2 mg, 0.20 mmol) and methyl 2-mercaptobenzoate (28.0 µL, 0.2 mmol). The product was obtained upon purification by column chromatography on silica gel (*n*-heptane/EtOAc (85:15)) as a colorless oil (43.7 mg, 87%).

^{1}H NMR-{^{13}C}NMR (400 MHz, CDCl$_3$) δ 8.00 (dd, *J* = 7.8, 1.2 Hz, 1H), 7.59 (m, 2H), 7.45 (m, 3H), 7.26 (dd, *J* = 11.8, 4.8 Hz, 1H), 7.15 (t, *J* = 7.5 Hz, 1H), 6.84 (d, *J* = 8.1 Hz, 1H), 3.98 (s, 3H), presence of traces of grease.

^{13}C NMR (101 MHz, CDCl$_3$) δ 167.0, 143.4, 136.7-126.8 (m, 6x ^{13}C and 4x ^{12}C, 10C), 124.4, 52.3.

HRMS (ESI-TOF) m/z Calcd for $^{13}C_6C_8H_{12}O_2S$ [M]$^+$ 250.0762; Found 250.0761.

IR (cm^{-1}): 2949, 1714, 1587, 1562, 1530, 1434, 1410, 1270, 1250, 1189, 1143, 1105, 1056, 1041, 994, 964, 824, 710, 686, 674, 653, 528.

4-Methyl-7-((phenyl-$^{13}C_6$)thio)-2H-chromen-2-one ([$^{13}C_6$]21)

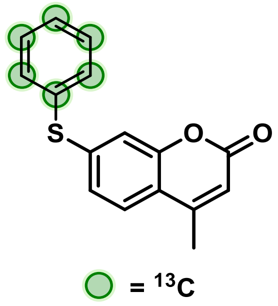

$C_{10}{}^{13}C_6H_{12}O_2S$
MW: 274.08g.mol^{-1}
Yield: 62 %
Yellow solid

The 4-methyl-7-((phenyl-13C_6)thio)-2H-chromen-2-one (**[$^{13}C_6$]23**) was prepared according to the general procedure GP3, using **[$^{13}C_6$]1** (77.2 mg, 0.20 mmol) and 7-mercapto-4-methyl-2H-chromen-2-one (38.4 mg, 0.2 mmol. The product was obtained upon purification column chromatography on silica gel (*n*-heptane/EtOAc (85:15)) as a yellow solid (34.0 mg, 62%).

^{1}H NMR-{^{13}C}NMR (400 MHz, CDCl$_3$) δ 7.61 – 7.44 (m, 2H), 7.46 – 7.37 (m, 4H), 7.10 (dd, J = 8.4, 1.6 Hz, 1H), 6.98 (d, J = 1.2 Hz, 1H), 6.20 (s, 1H), 2.39 (s, 3H), presence of traces of grease.

^{13}C NMR (101 MHz, CDCl$_3$) δ 158.0, 154.0, 152.2, 135.1-129.1 (m, 6x ^{13}C and 3x ^{12}C, 9C) 124.9, 117.3, 114.2, 18.7.

HRMS (ESI-TOF) m/z Calcd for $^{13}C_6C_{10}H_{13}O_2S$ [M+H]$^+$ 275.084; Found 275.0842.

IR (cm^{-1}): 2962, 2921, 1713, 1682, 1622,1600, 1547, 1490, 1443, 1392, 1366, 1321, 1258, 1170, 1154, 1053, 1011, 963, 844, 818, 789, 772, 742, 708, 699, 668, 577, 522, 495, 495, 486, 423.

Mp = 134 °C.

5-(4-(4-methoxyphenyl)-phenyl-$^{13}C_6$)-5*H*-thianthren-5-iumtrifluoromethanesulfonate ([$^{13}C_6$]22)

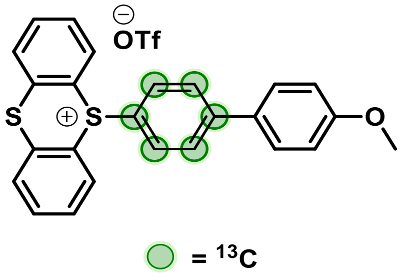

$C_{20}{}^{13}C_6H_{19}F_3O_4S_3$
MW: 549.05 g.mol^{-1}
Yield: 30 %
Yellow solid

The 5-(4-(4-methoxyphenyl)-phenyl-$^{13}C_6$)-5*H*-thianthren-5-iumtrifluoromethanesulfonate (**[$^{13}C_6$]24**) was synthetized according to the literature. [7] A 20 mL Schlenk tube was charged with thianthrene-*S*-oxide (371 mg, 1.6 mmol, 1.1 equiv.), DCM (7 mL) and 4-methoxy-1,1'-biphenyl (278 mg, 1.5 mmol, 1.0 equiv.) under N_2 atmosphere. The reaction mixture was cooled to -40 °C and stirred at this temperature. Tf_2O (1.8 mmol, 1.2 equiv) was then added dropwise. The reaction mixture was stirred at -40 °C for 30 min, and then allowed to stir at room temperature for 12 h followed by its neutralization by a saturated aqueous $NaHCO_3$ solution, and extracted with DCM. The combined organic layers were dried over anhydrous Na_2SO_4 and concentrated to dryness under reduced pressure. The crude product was purified by column chromatography on silica gel (DCM/MeOH (97:3)) affording the product as yellow solid (250 mg, 30%).

^{1}H NMR -{^{13}C}NMR (400 MHz, CD_3CN) δ 8.37 (d, *J* = 7.9 Hz, 2H), 7.98 (d, *J* = 7.9 Hz, 2H), 7.91 (t, *J* = 7.7 Hz, 2H), 7.83 (t, *J* = 7.7 Hz, 2H), 7.70 (d, *J* = 8.4 Hz, 2H), 7.56 (d, *J* = 8.8 Hz, 2H), 7.17 (d, *J* = 8.2 Hz, 2H), 7.01 (d, *J* = 8.8 Hz, 1H), 3.82 (s, 3H).

^{13}C NMR (101 MHz, CD_3CN) δ 159.2, 146.6-121.5 (m, 6× ^{13}C and 15× ^{12}C, 21C), 118.3 (2C), 56.1.

^{19}F NMR (376 MHz, CD_3CN): -78.1 (s).

HRMS (ESI-TOF) m/z Calcd for $^{13}C_6C_{19}H_{19}OS_2$ $[M]_+$ 405.1081; Found 405.108.0

IR (cm^{-1}): 3073, 1605, 1513, 1451, 1259, 1223, 1154, 1029, 811, 763, 637, 600, 517, 465, 410.

Mp = 85 °C.

Ethyl 2-((phenyl-$^{13}C_6$)thio)acetate [$^{13}C_6$]23)

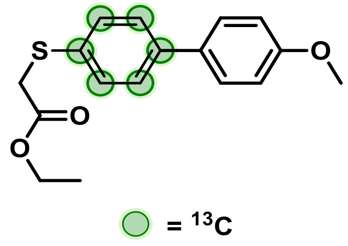

$C_{11}{}^{13}C_6H_{18}O_3S$
MW: 308.12 g.mol^{-1}
Yield: 65 %
Yellow oil

The ethyl 2-((phenyl-$^{13}C_6$)thio)acetate **[$^{13}C_6$]25** was prepared according to the general procedure GP3, using **[$^{13}C_6$]24** (27.7 mg, 0.05 mmol) and ethyl thioglycolate (11.1 µL, 0.1 mmol) The crude reaction mixture was purified by column chromatography on silica gel (*n*-heptane/EtOAc (90:10)) affording the product as a yellow oil (10.1 mg, 65%).

^{1}H NMR-{^{13}C}NMR (400 MHz, CDCl$_3$) δ 7.55 – 7.38 (m, 6H), 6.97 (d, J = 8.6 Hz, 2H), 4.18 (q, J = 7.1 Hz, 2H), 3.85 (s, 3H), 3.65 (s, 2H), 1.24 (t, J = 7.1 Hz, 3H).

^{13}C NMR (101 MHz, CDCl$_3$) δ 169.9, 160.1, 140.4-126.3 (m, 6^{13}C and 3^{12}C, 9C), 114.4, 114.4, 61.7, 55.5, 29.8, 14.3.

HRMS (ASAP-TOF) m/z Calcd for $^{13}C_6C_{11}H_{18}O_3S$ [M]$^+$ 308.1181; Found 308.1179.

IR (cm^{-1}): 2959, 2931, 2837, 1888, 1732, 1606, 1576, 1543, 1514, 1494, 1460, 1442, 1411, 1385, 1367, 1280, 1249, 1177, 1152¸1069¸1032¸ 976, 944¸ 894, 839, 802¸ 752, 719, 686, 638, 604, 585¸ 545, 492, 456, 439, 405.

1-Methoxy-4-(phenyl-$^{13}C_6$)benzene- 4'-D [$^{13}C_6$, $^2H_5$]24)

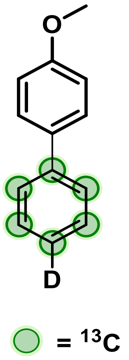

$C_7{}^{13}C_6H_{11}DO$
MW: 191.11 g.mol^{-1}
Yield: 85 %
White solid

1-Methoxy-4-(phenyl-$^{13}C_6$)benzene-4′-D **[$^{13}C_6$, $^2H_5$] 26** was prepared using a 4 mL pressure tube equipped with a magnetic stir bar. To the tube were added **[$^{13}C_6$]24** (27.7 mg, 0.05 mmol), DCOONa (6.9 mg, 0.1 mmol), and Pd/C (10 wt.% loading, dry basis, 2.7 mg, 0.0025 mmol, 5 mol%) in DMF (0.6 mL). The crude reaction mixture was purified by column chromatography on silica gel (n-heptane, 100%), affording the product as a white solid (8.2 mg, 85%).[8]

1H NMR-{^{13}C}NMR (400 MHz, $CDCl_3$) δ 7.55 (t, J = 8.0 Hz, 4H), 7.42 (d, J = 7.7 Hz, 2H), 6.99 (d, J = 8.7 Hz, 2H), 3.86 (s, 3H), presence of impurities.

^{2}H NMR (61 MHz, $CHCl_3$) δ 7.32 (s, 1H).

13C NMR (101 MHz, $CDCl_3$) δ 159.3, 141.6-125.7 (m, 6x ^{13}C and 3x ^{12}C, 9C), 114.3, 114.3, 114.3, 55.5.

HRMS (ASAP-TOF) m/z Calcd for $^{13}C_6C_7H_{11}DO$ $[M]^+$ 191.1155; Found 191.1156.

IR (cm^{-1}): 3051, 3001, 2958, 2930, 2836, 1890, 1730, 1606, 1577, 1515, 1458, 1440, 1372, 1306, 1282, 1251, 1197, 1183, 1125, 1080, 1080, 1036, 981, 824, 810, 754, 739, 702, 684, 598, 564, 543, 476, 416.

Mp = 78 °C.

Ethyl (*E*)-3-(2,6-dibenzyl(phenyl-$^{13}C_6$)acrylate ([$^{13}C_6$]25)

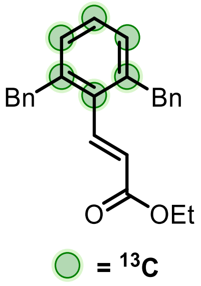

$C_{19}{}^{13}C_6H_{24}O_2$
MW: 362.42 g.mol^{-1}
Yield: 55%
White solid

The compound **[$^{13}C_6$]27** was prepared according to the general procedure GP5, using **[$^{13}C_6$]1** (77.2 mg, 0.20 mmol), ethyl acrylate (33 µL, 0.30 mmol, 1.5 equiv.) as the terminating reagent and BnBr (71 µL, 0.6 mmol, 3.0 equiv.) as the electrophile. It was purified by flash column chromatography on silica gel (*n*-heptane/EtOAc (95:5)) followed by a reverse-phase flash column chromatography (eluent: CH_3CN/H_2O 50:50 to 100:0) affording the product as a white solid (39.9 mg, 55%).

$^{1}H\{^{13}C\}$ NMR (400 MHz, $CDCl_3$) δ 7.77 (d, J = 16.4 Hz, 1H), 7.33-7.15 (m, 7H), 7.15-6.99 (m, 6H), 5.85 (d, J = 16.4 Hz, 1H), 4.23 (q, J = 7.2 Hz, 2H), 4.02 (s, 4H), 1.31 (t, J = 7.2 Hz, 3H).

^{13}C NMR (100 MHz, $CDCl_3$): δ 166.2 (d, J = 6.0 Hz), 143.2 (d, J = 52.9 Hz), 140.5 (m, 1^{12}C), 140.3-137.8 (m, 2^{13}C, 2C), 135.6-134.1 (m, 1^{13}C), 129.5-127.2 (m, 8^{12}C, 3^{13}C), 126.1, 124.9 (t, J = 2.7 Hz, 1^{12}C, 1H), 60.5, 40.0-39.4 (m, 2^{12}C), 14.3.

HRMS (ESI-TOF) *m/z* Calcd for $^{13}C_6C_{19}H_{25}O_2[M+H]^+$ 363.2058; Found 363.2056.

IR (cm^{-1}): 2983, 1707, 1642, 1600, 1494, 1411, 1365, 1308, 1275, 1181, 1031, 986, 766, 739, 702.

Mp = 114 °C.

Ethyl (*E*)-3-(2,6-dibenzyl(phenyl-D$_3$)acrylate ([^{2}H$_3$]25)

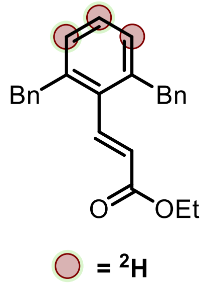

$C_{25}H_{21}D_3O_2$
MW: 359.48 g.mol^{-1}
Yield: 42%
Colorless oil

The compound **[^{2}H$_3$]27** was prepared accordingly to general procedure GP5, using **[^{2}H$_6$]1** (77.0 mg, 0.20 mmol), ethyl acrylate (33 µL, 0.30 mmol, 1.5 equiv.) as the terminating reagent and BnBr (71 µL, 0.6 mmol, 3.0 equiv.) as the electrophile. It was purified by PTLC (*n*-heptane/EtOAc (95:5), double elution) followed by a reverse-phase flash column chromatography (eluent: CH_3CN/H_2O 50:50 to 100:0) affording the product as a colorless oil (30.1 mg, 42%).

^{1}H NMR (400 MHz, CDCl$_3$) δ 7.77 (d, J = 16.4 Hz, 1H), 7.30-7.23 (m, 4H), 7.22-7.16 (m, 2H), 7.12-7.07 (m, 4H), 5.85 (d, J = 16.4 Hz, 1H), 4.22 (q, J = 7.2 Hz, 2H), 4.01 (s, 4H), 1.30 (t, J = 7.2 Hz, 3H), presence of traces of ethyl acetate and acetone.

^{2}H NMR (400 MHz, CDCl$_3$) δ 7.21, 7.10.

^{13}C NMR (100 MHz, CDCl$_3$): δ 166.1, 143.3, 140.5 (2C), 139.0 (2C), 134.9, 128.9 (4C), 128.4 (4C), 126.1 (2C), 124.9, 60.5, 39.7 (2C), 14.3.

HRMS (ESI-TOF) *m/z* Calcd for $C_{25}H_{22}D_4O_2$[M+H]$^+$ 360.2043; Found 360.2044.

IR (cm^{-1}): 2980, 1713, 1641, 1602, 1494, 1365, 1304, 1267, 1174, 1030, 989, 776, 731.

4-(2,6-Dibenzyl(phenyl-$^{13}C_6$))butan-2-one ([$^{13}C_6$]26)

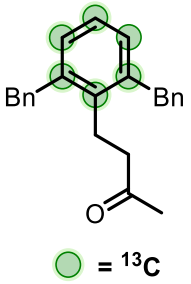

$C_{18}{}^{13}C_6H_{24}O$
MW: 328.45 g.mol^{-1}
Yield: 60%
Colorless oil

The compound **[$^{13}C_6$]28** was prepared accordingly to general procedure GP5, using **[$^{13}C_6$]1** (77.2 mg, 0.20 mmol), but-3-en-2-ol (26 μL, 0.30 mmol, 1.5 equiv.) as the terminating reagent and BnBr (71 μL, 0.6 mmol, 3.0 equiv.) as the electrophile. It was purified by flash column chromatography on silica gel (*n*-heptane/EtOAc (90:10)) followed by a reverse-phase flash column chromatography (eluent: CH_3CN/H_2O 50:50 to 100:0) affording the product as a colorless oil (39.4 mg, 60%).

$^1H\{^{13}C\}$ NMR (400 MHz, $CDCl_3$) δ 7.32-7.22 (m, 4H), 7.21-7.02 (m, 9H), 4.00 (s, 4H), 2.82-2.81 (m, 2H), 2.05-1.96 (m, 2H), 1.85 (s, 3H).

^{13}C NMR (100 MHz, $CDCl_3$): δ 207.8 (d, $J_{C\text{-}C}$ = 3.7 Hz), 140.0 (2C), 139.9-137.9 (2^{13}C), 130.3-128.6 (m, 4^{12}C, 3^{13}C), 128.4 (4^{12}C), 126.8-125.5 (1^{13}C, 2^{12}C), 43.1, 39.8-39.2 (m), 29.5, 23.5-22.9 (m).

HRMS (ESI-TOF) *m/z* Calcd for $^{13}C_6C_{18}H_{25}O$ $[M+H]^+$ 335.2109; Found 335.2106.

IR (cm^{-1}): 3061, 3025, 1712, 1641, 1602, 1494, 1442, 1360, 1160, 1075, 1029, 767, 742, 727.

Dimethyl 4,4'-(1,3-(phenyl-$^{13}C_6$)enebis(methylene))dibenzoate ([$^{13}C_6$]27)

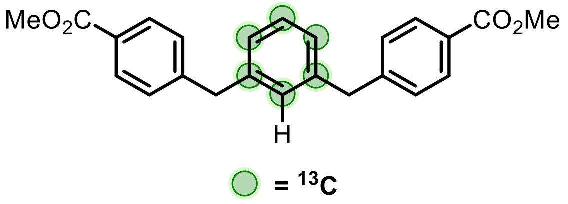

$C_{18}{}^{13}C_6H_{22}O_4$
MW: 374.44 g.mol^{-1}
Yield: 52%
Yellow oil

The compound **[$^{13}C_6$]29** was prepared accordingly to general procedure GP5, using **[$^{13}C_6$]1** (77.2 mg, 0.20 mmol), *iso*propanol (26 µL, 0.30 mmol, 1.5 equiv.) as the terminating reagent and methyl 4-(bromomethyl)benzoate (137.4 mg, 0.6 mmol, 3.0 equiv.) as the electrophile. It was purified by flash column chromatography on silica gel (*n*-heptane/EtOAc (90:10)) followed by a reverse-phase flash column chromatography (eluent: CH_3CN/H_2O 50:50 to 100:0) affording the product as a yellow oil (38.7 mg, 52%).

$^1H\{^{13}C\}$ NMR (400 MHz, $CDCl_3$) δ 7.95 (d, *J* = 8.0 Hz, 4H), 7.25-7.17 (m, 5H), 7.05-6.97 (m, 3H), 3.99 (s, 4H), 3.90 (s, 6H).

^{13}C NMR (100 MHz, $CDCl_3$): δ.167.0 (2C), 146.4 (d, *J* = 2.0 Hz, $2^{12}C$), 141.2-126.0 (m, $10^{12}C$, $6^{13}C$, 16C), 52.0 (2C), 42.1-41.5 (m, 2C).

HRMS (ESI-TOF) ***m/z*** Calcd for $^{13}C_6C_{18}H_{22}O_4Na$ $[M+Na]^+$ 403.1620; Found 403.1616.

IR (cm^{-1}): 2950, 1715, 1610, 1551, 1433, 1415, 1273, 1177, 1101, 1020, 761, 731, 709.

1,3-Dimorpholino(benzene-$^{13}C_6$) ([$^{13}C_6$]28)

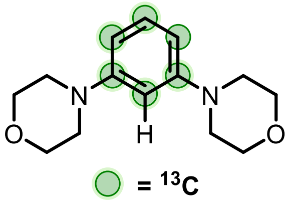

$C_8{}^{13}C_6H_{20}N_2O_2$
MW: 254.28 g.mol^{-1}
Yield: 54%
Dark brown

According to the modified literature procedure, a 10 mL microwave tube was sequentially charged with **[^{13}C$_6$]1** (77.2 mg, 0.2 mmol, 1.0 equiv.), Cs_2CO_3 (260.7 mg, 0.8 mmol, 4.0 equiv.), *N*-morpholinobenzoate (124.3 mg, 0.6 mmol, 3.0 equiv.), $Pd(OAc)_2$ (4.5 mg, 0.02 mmol, 10 mol%), (4-Cl-$C_6H_4)_3P$ (12.6 mg, 0.04 mmol, 20 mol%), norbornene (56.5 mg, 0.6 mmol, 3.0 equiv.). The tube was then evacuated and refilled with argon 3 times. It was then charged with *iso*propanol (23 µL, 0.3 mmol, 1.5 equiv.) and dry toluene (2.0 mL). The tube was then sealed and heated in a pre-heated pie-block at 100 °C for 16 h and allowed to cool down to RT, followed by a filtration over a silica pad (eluent : EtOAc). The crude reaction mixture was then purified by flash chromatography (eluent: EtOAc/heptane 7:3), affording the product **[^{13}C$_6$]30** as a dark brown oil (27.5 mg, 54%).

$^{1}H\{^{13}C\}$ NMR (400 MHz, $CDCl_3$) δ 7.18 (t, *J* = 8.0 Hz, 1H), 6.53-6.41 (m, 3H), 3.91-3.79 (m, 8H), 3.20-3.09 (m, 8H).

^{13}C NMR (100 MHz, $CDCl_3$): δ.153.2-151.6 (m, 2^{13}C), 130.7-128.7 (m, 1^{13}C), 109.2-107.0 (m, 2^{13}C), 104.9-102.7 (m, 1^{13}C), 67.0-66.9 (m, 4C), 49.7-49.6 (m, 4C).

HRMS (ESI-TOF) *m/z* Calcd for $^{13}C_6C_8H_{21}N_2O_2$ $[M+H]^+$ 255.1807; Found 255.1808.

IR (cm^{-1}): 2957, 2851, 2817, 1542, 1524, 1447, 1377, 1264, 1192, 1069, 979, 915, 868, 754.

5. NMR Spectra

^{1}H NMR (400 MHz, CD_3CN), Thianthrene S-oxide **(2):**

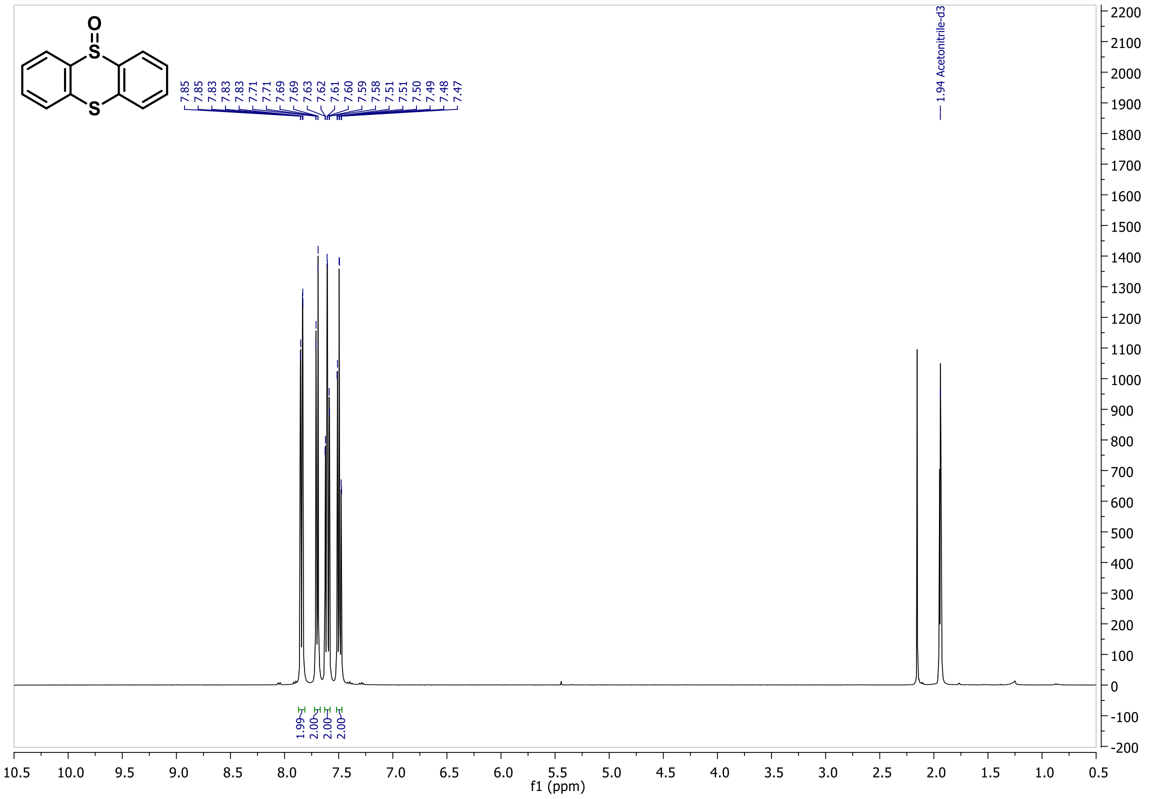

^{1}H NMR (400 MHz, $CDCl_3$), Thianthrene S-oxide **(2):**

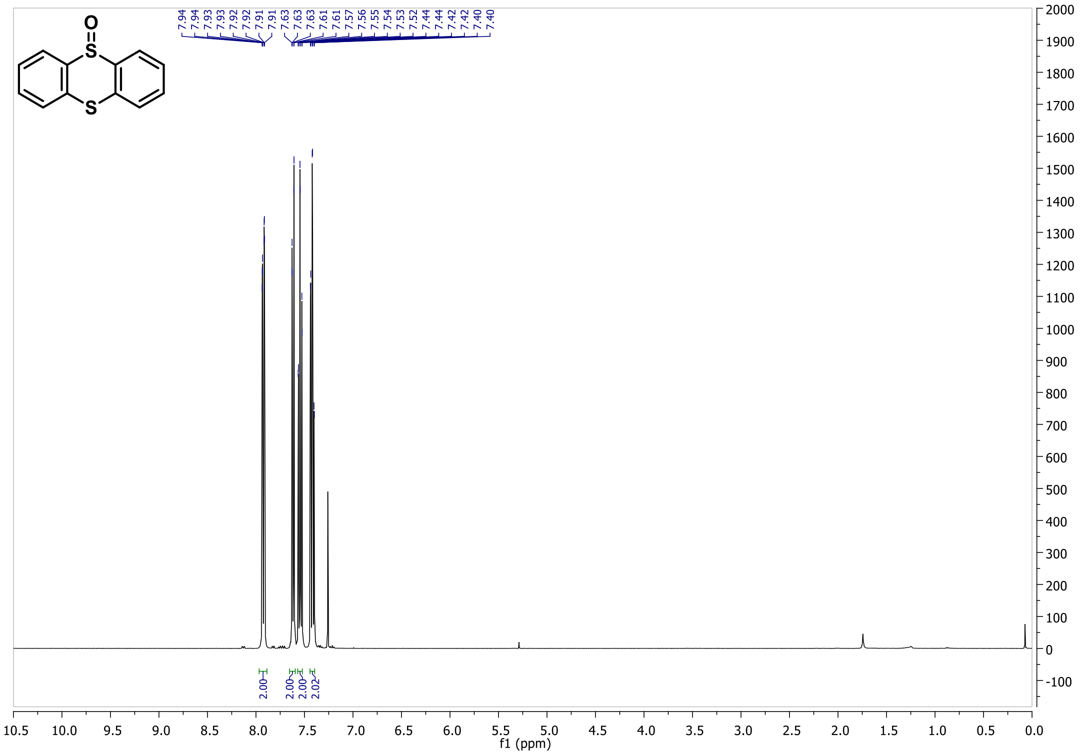

^{1}H NMR (400 MHz, CD_3CN), 5-phenyl-5*H*-thianthren-5-ium tetrafluoroborate (**1**)

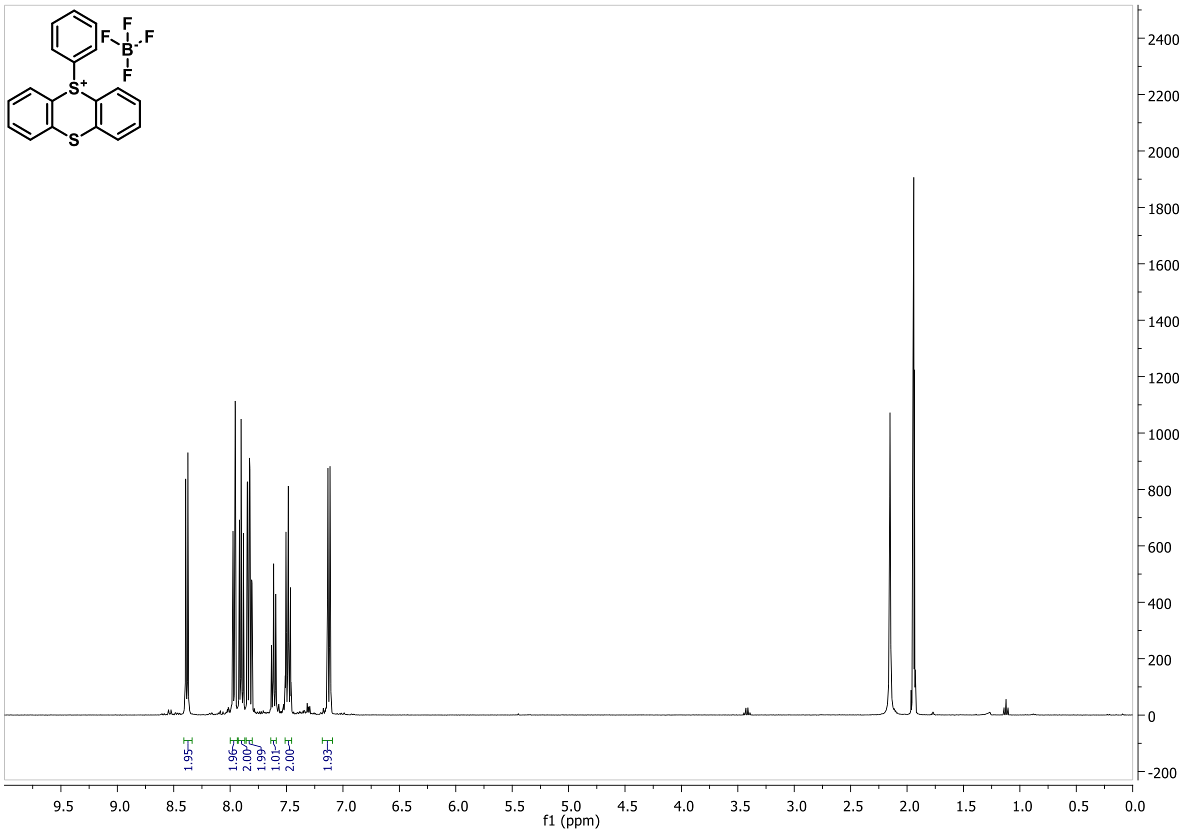

^{13}C NMR (100 MHz, CD_3CN), 5-phenyl-5*H*-thianthren-5-ium tetrafluoroborate (**1**)

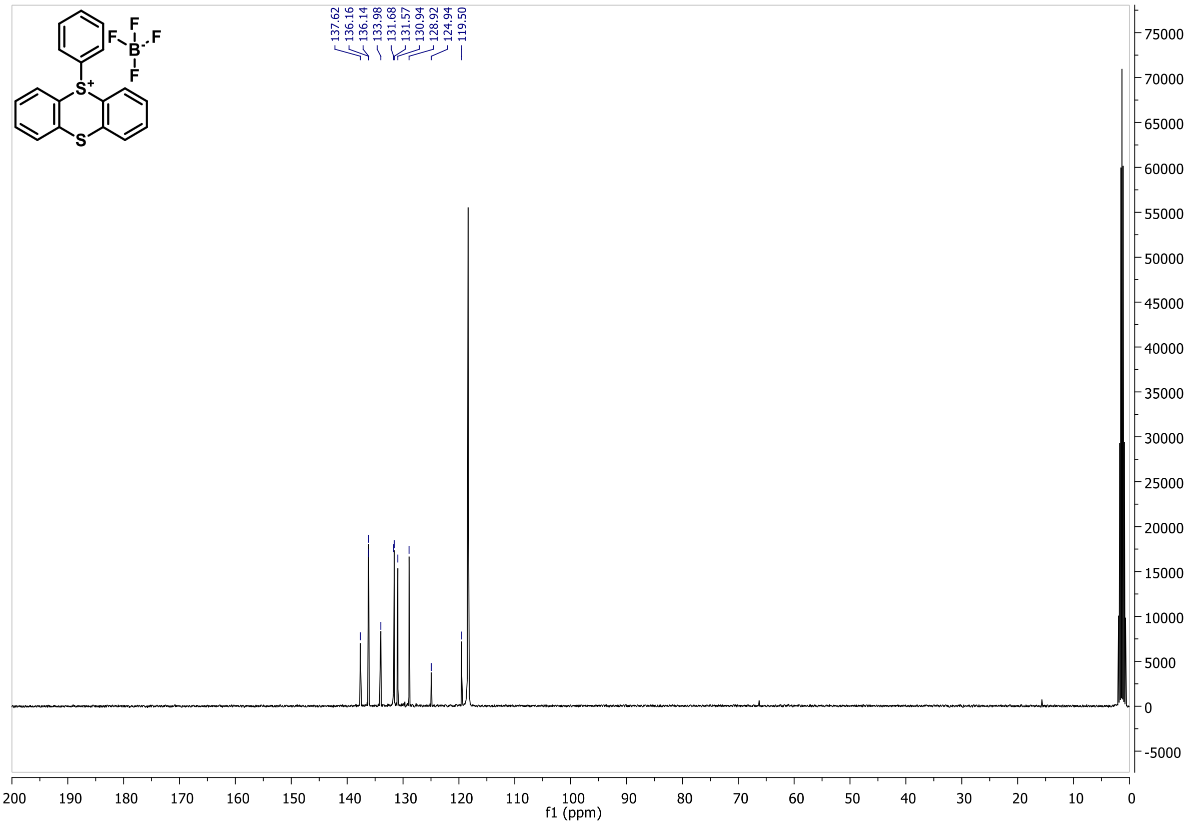

^{1}H NMR (400 MHz, CD_3CN), 5-(phenyl-*d5*)-5*H*-thianthren-5-ium BF_4^- (**[^{2}H$_5$]1**):

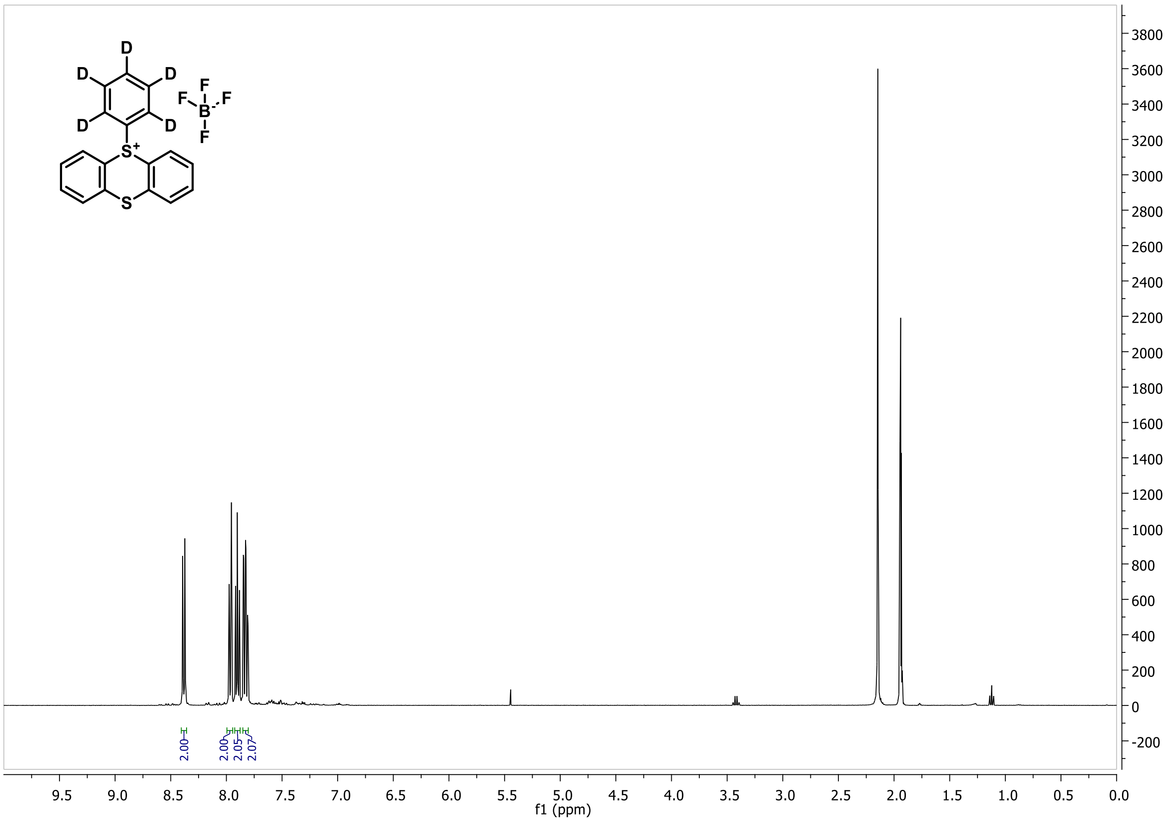

^{2}H NMR-{^{1}H}NMR (61 MHz, $CHCl_3$), 5-(phenyl-*d5*)-5*H*-thianthren-5-ium BF_4^- **[^{2}H$_5$]1**) :

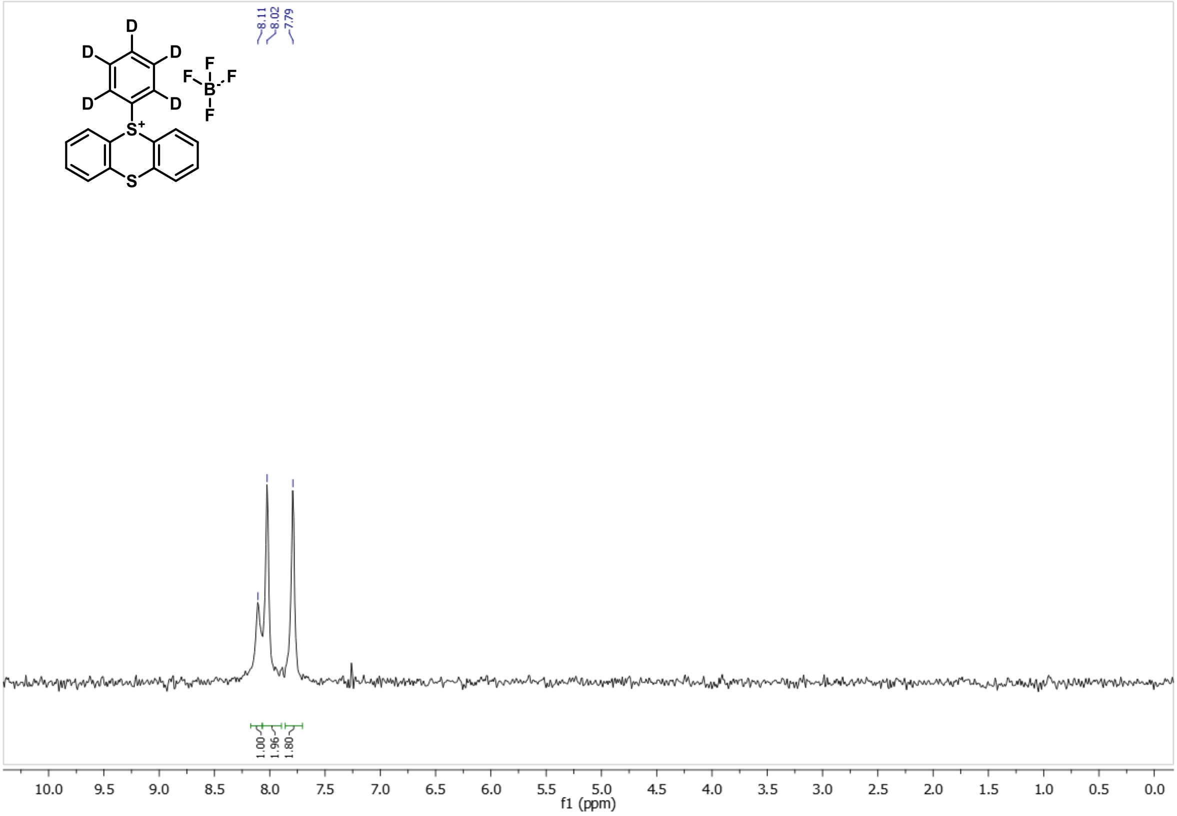

^{13}C NMR (100 MHz, CD_3CN), 5-(phenyl-*d5*)-5*H*-thianthren-5-ium BF_4^- (**[^{2}H$_5$]1**):

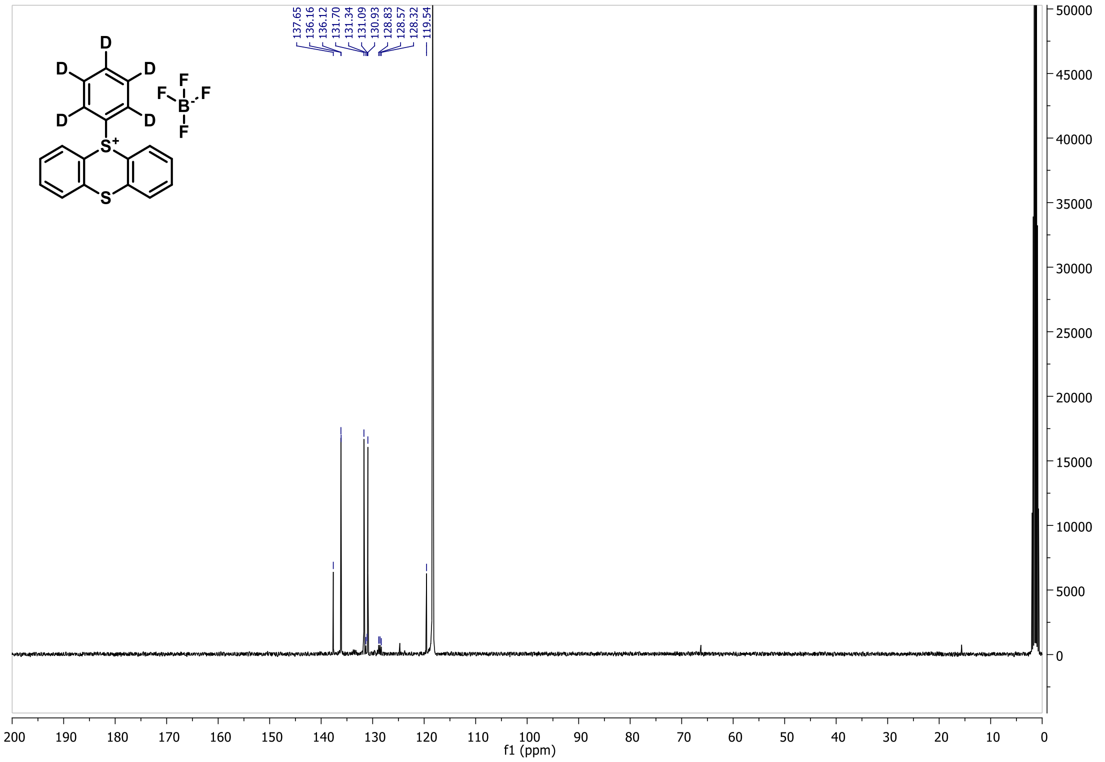

^{19}F NMR (376 MHz, CD_3CN), 5-(phenyl-*d5*)-5*H*-thianthren-5-ium BF_4^- (**[^{2}H$_5$]1**):

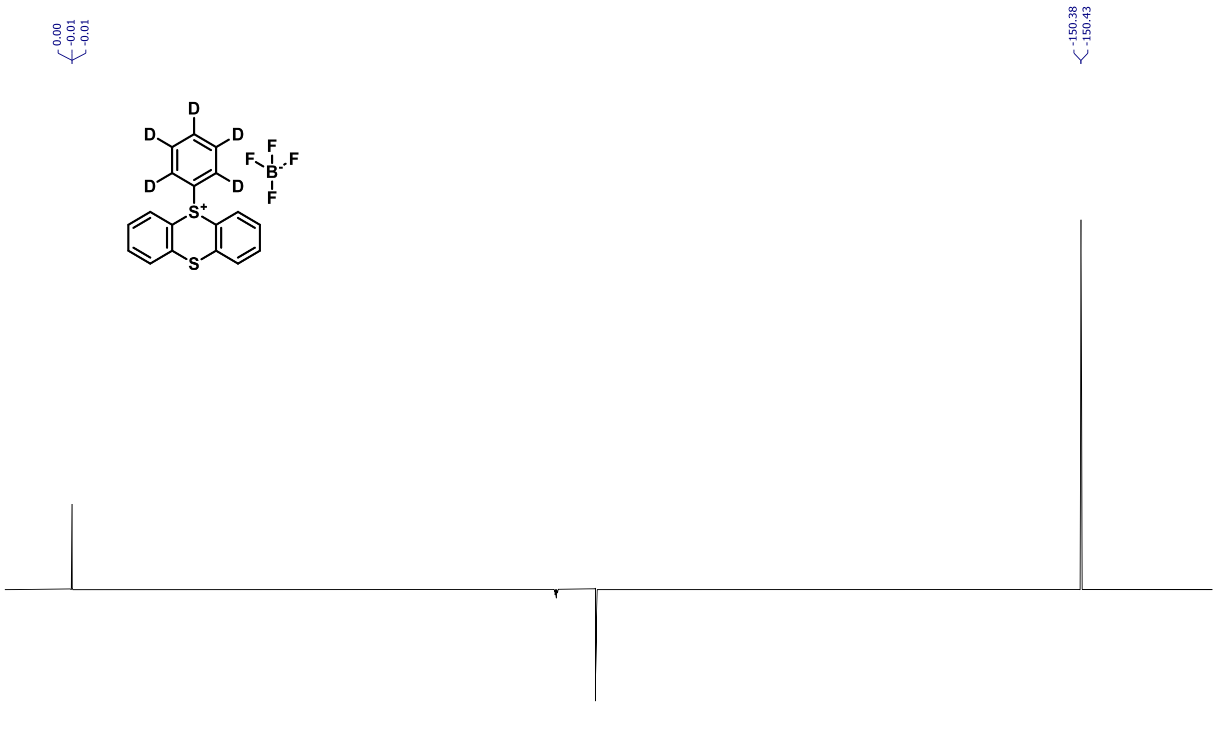

^{1}H NMR (400 MHz, CD_3CN), 5-(phenyl-$^{13}C_6$)-5*H*-thianthren-5-ium BF_4^- (**[^{13}C$_6$]1**)

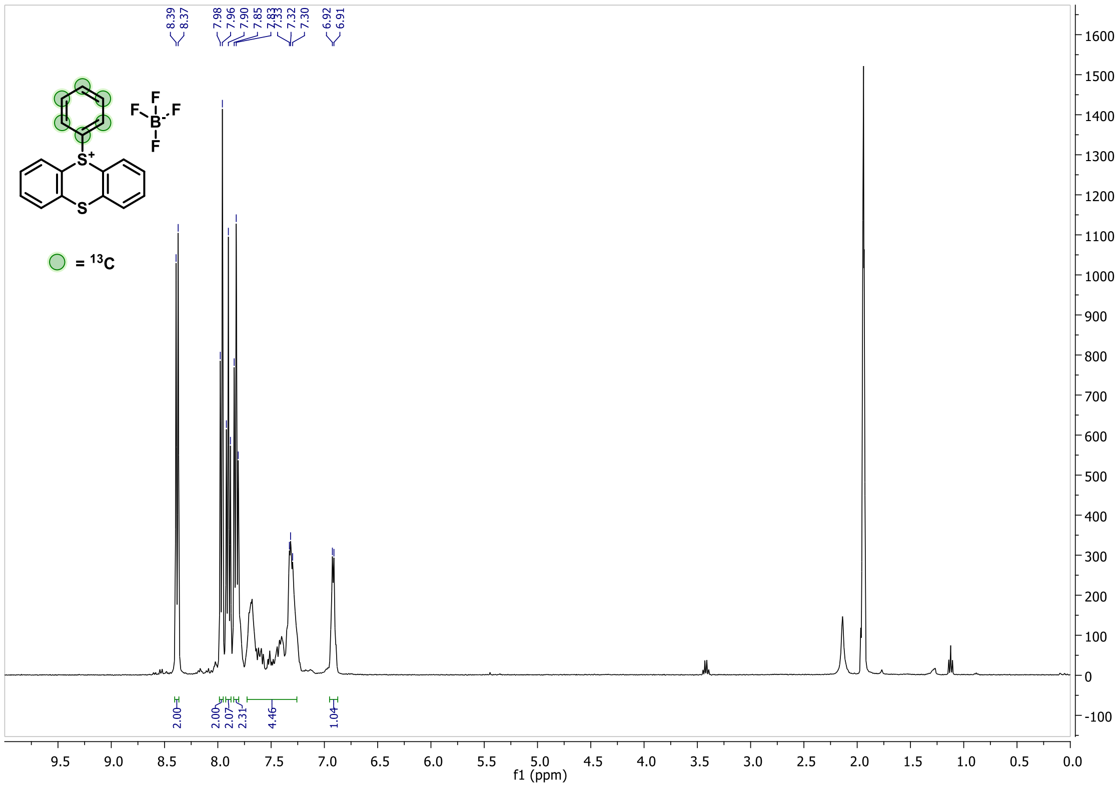

^{1}H NMR-{^{13}C}NMR (400 MHz), 5-([$^{13}C_6$]phenyl)-5H-thianthren-5-ium BF_4^- (**[^{13}C$_6$]1**)

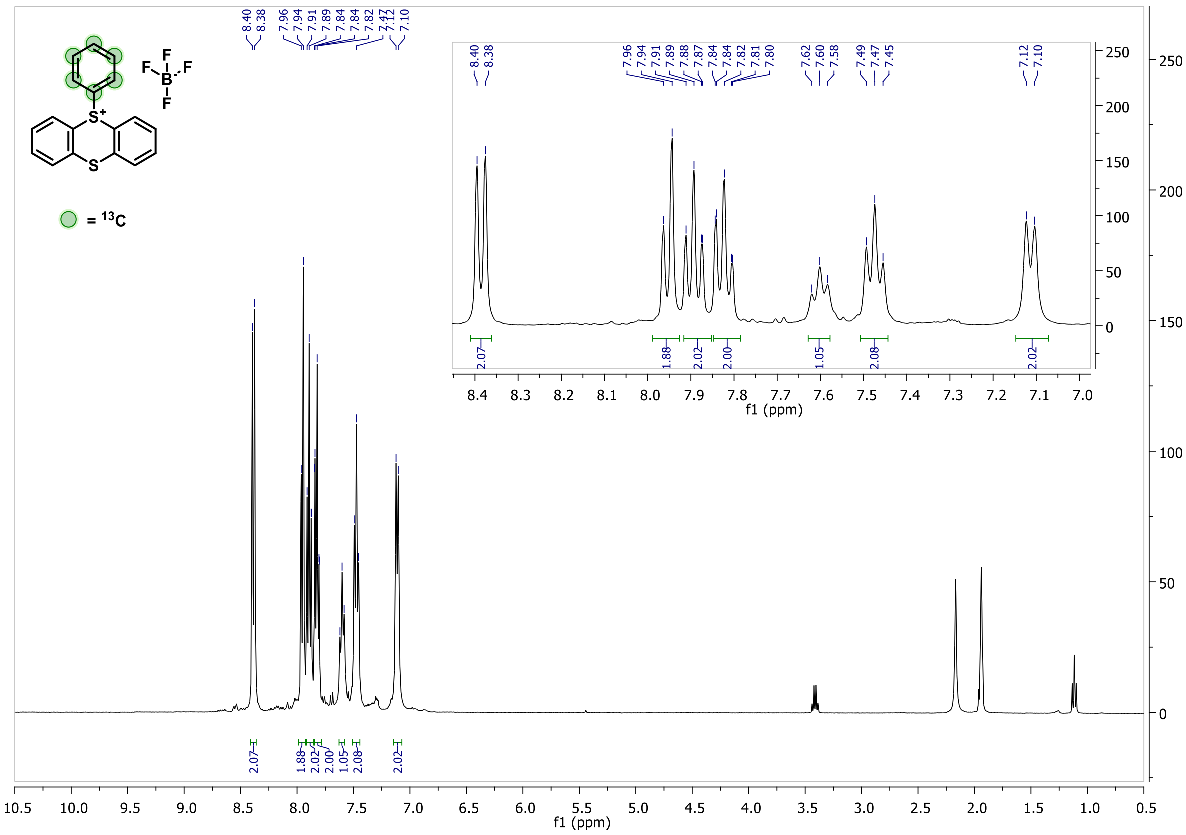

^{13}C NMR (100 MHz, CD_3CN), 5-([$^{13}C_6$]phenyl)-5H-thianthren-5-ium BF_4^- (**[$^{13}C_6$]1**):

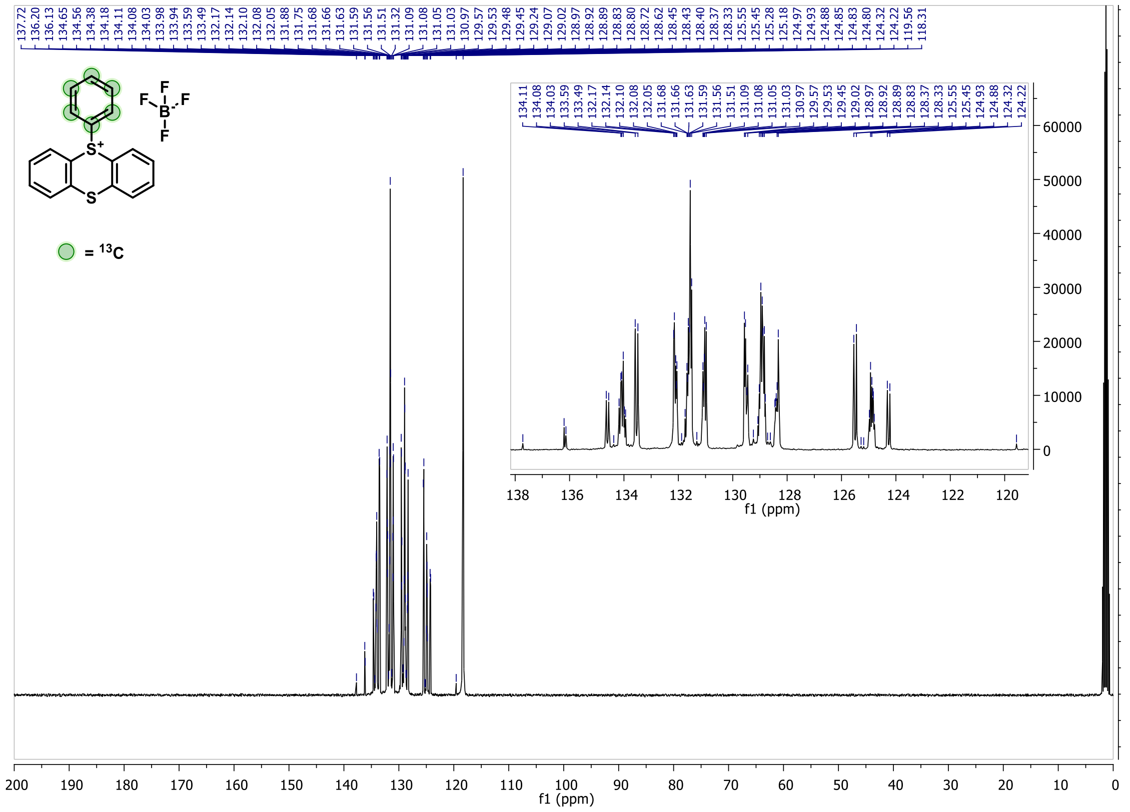

^{19}F NMR (376 MHz, CD_3CN), 5-([$^{13}C_6$]phenyl)-5H-thianthren-5-ium BF_4^- (**[$^{13}C_6$]1**):

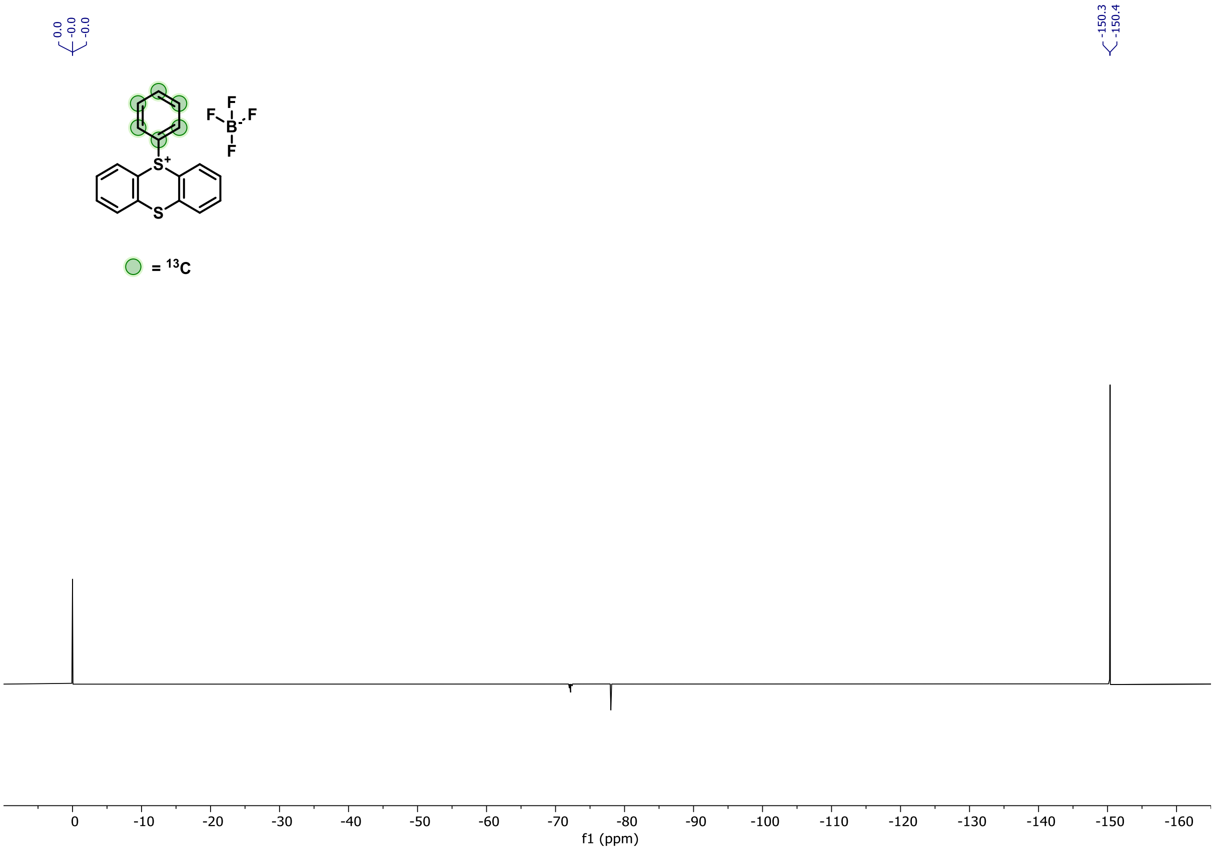

^{1}H NMR (400 MHz, $CDCl_3$), 4-methoxy-1,1'-biphenyl (**3**) :

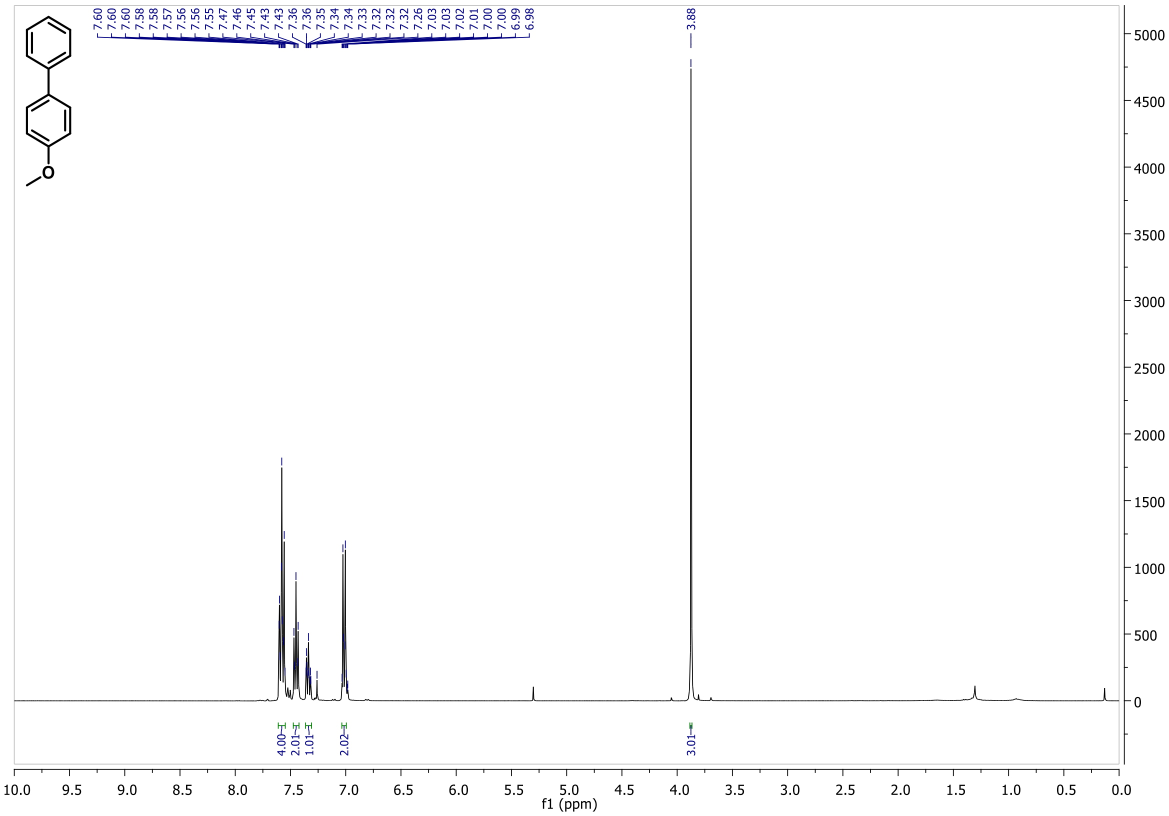

^{13}C NMR (100 MHz, $CDCl_3$),4-methoxy-1,1'-biphenyl (**3**):

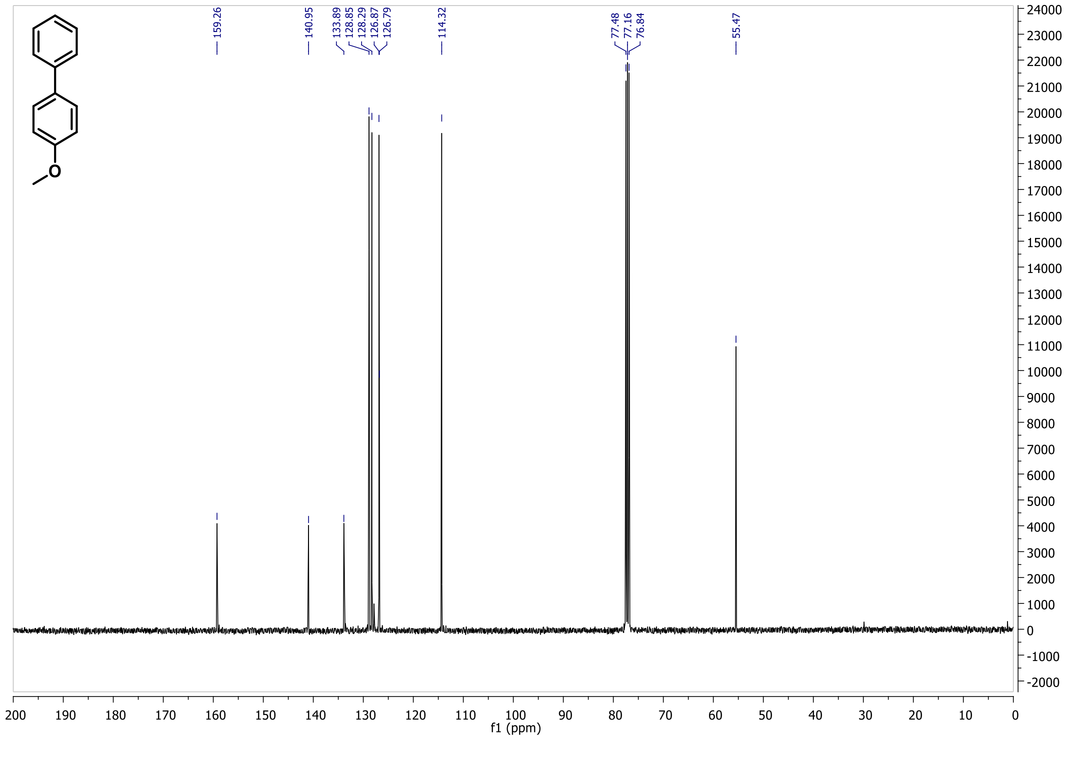

^1H NMR (400 MHz, $CDCl_3$), 4-methoxy-1,1'-biphenyl-2',3',4',5',6'-d_5 (**[$^2H_5$]3**)

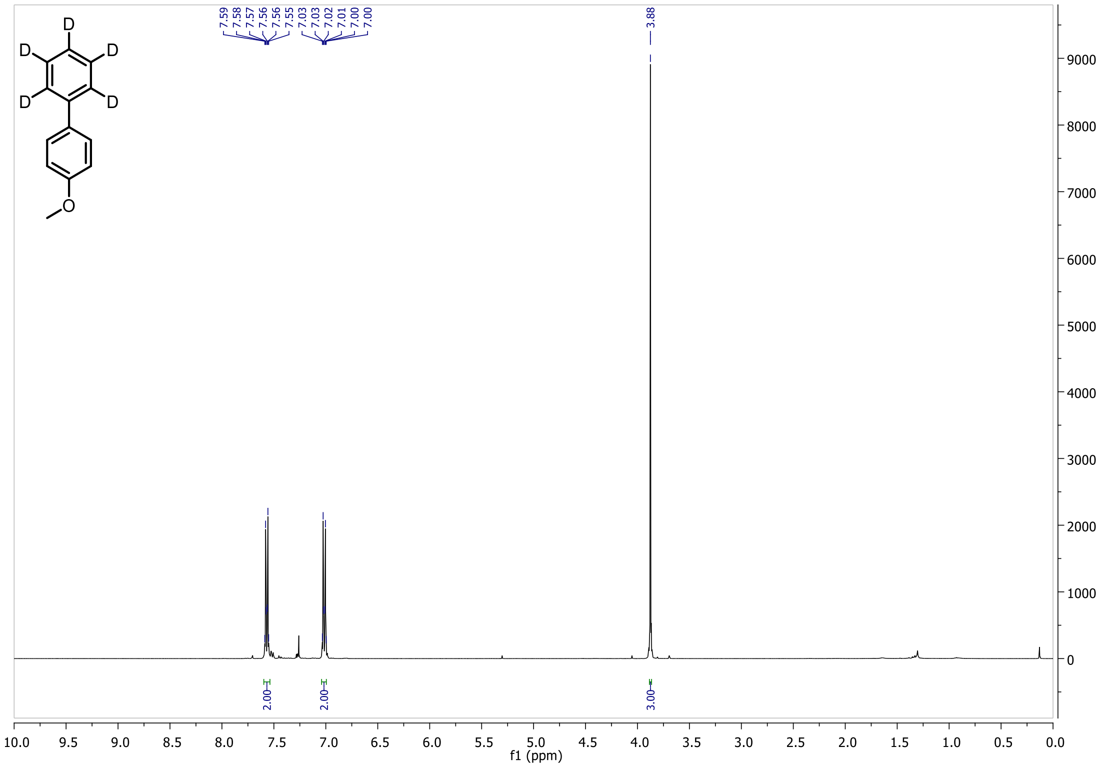

^2H NMR-{^1H}NMR (61 MHz, $CHCl_3$), 4-methoxy-1,1'-biphenyl-2',3',4',5',6'-d_5 (**[$^2H_5$]3**)

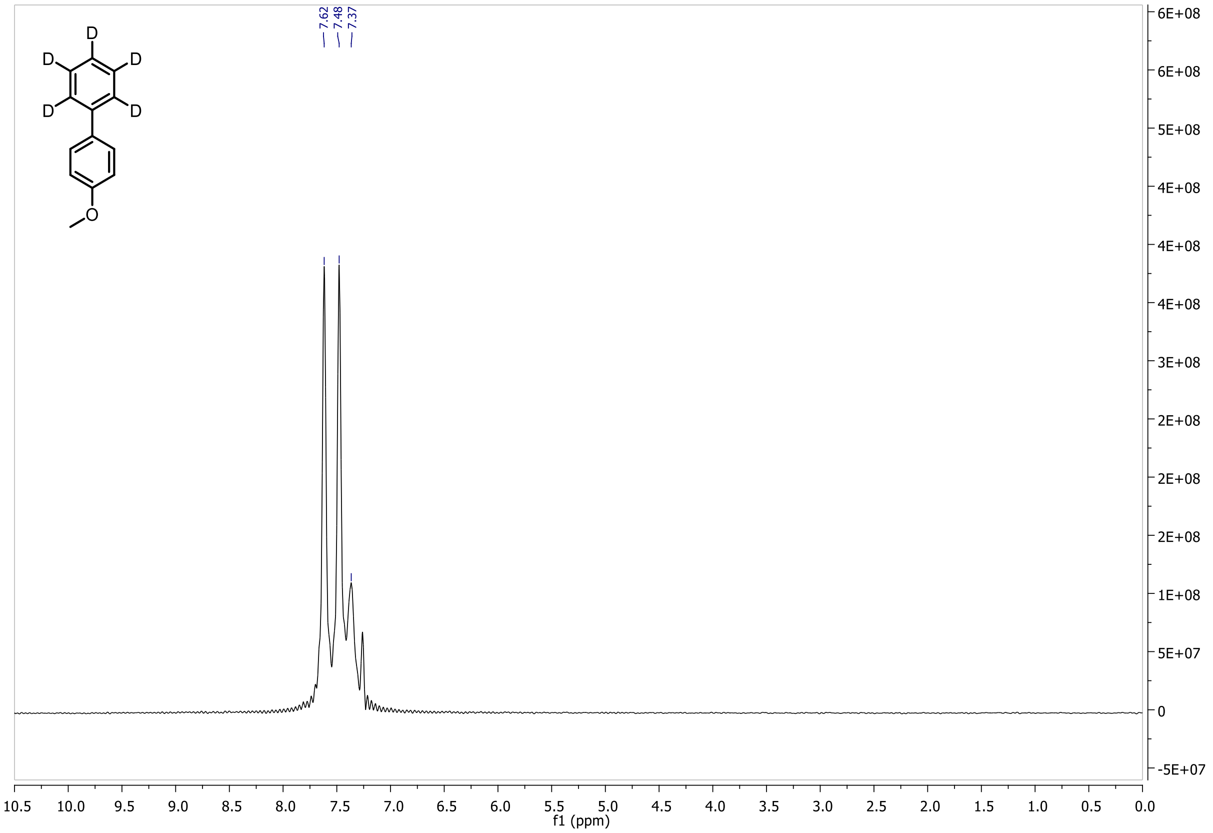

^{13}C NMR (100 MHz, CDCl$_3$), 4-methoxy-1,1'-biphenyl-2',3',4',5',6'-d_5 (**[^{2}H$_5$]3**)

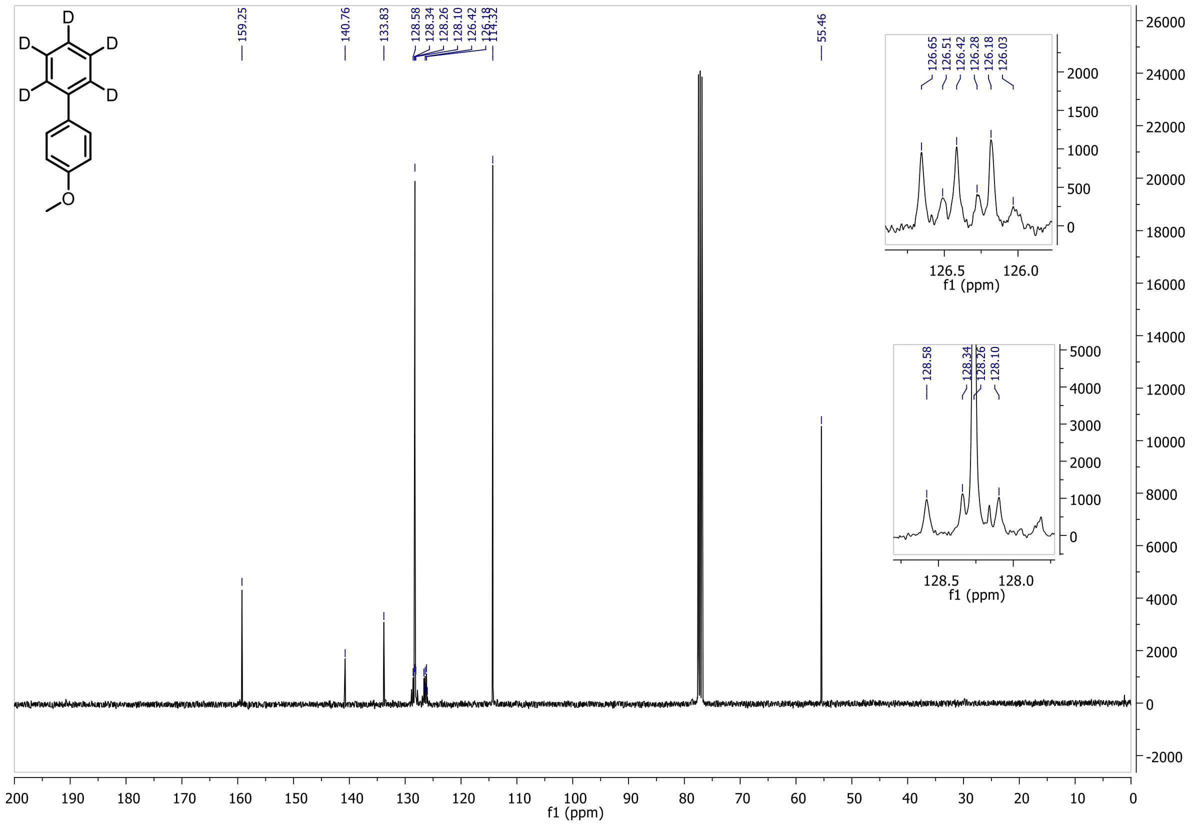

^{1}H NMR (400 MHz, CDCl$_3$), 1-methoxy-4-(phenyl-$^{13}C_6$)benzene (**[^{13}C$_6$]3**)

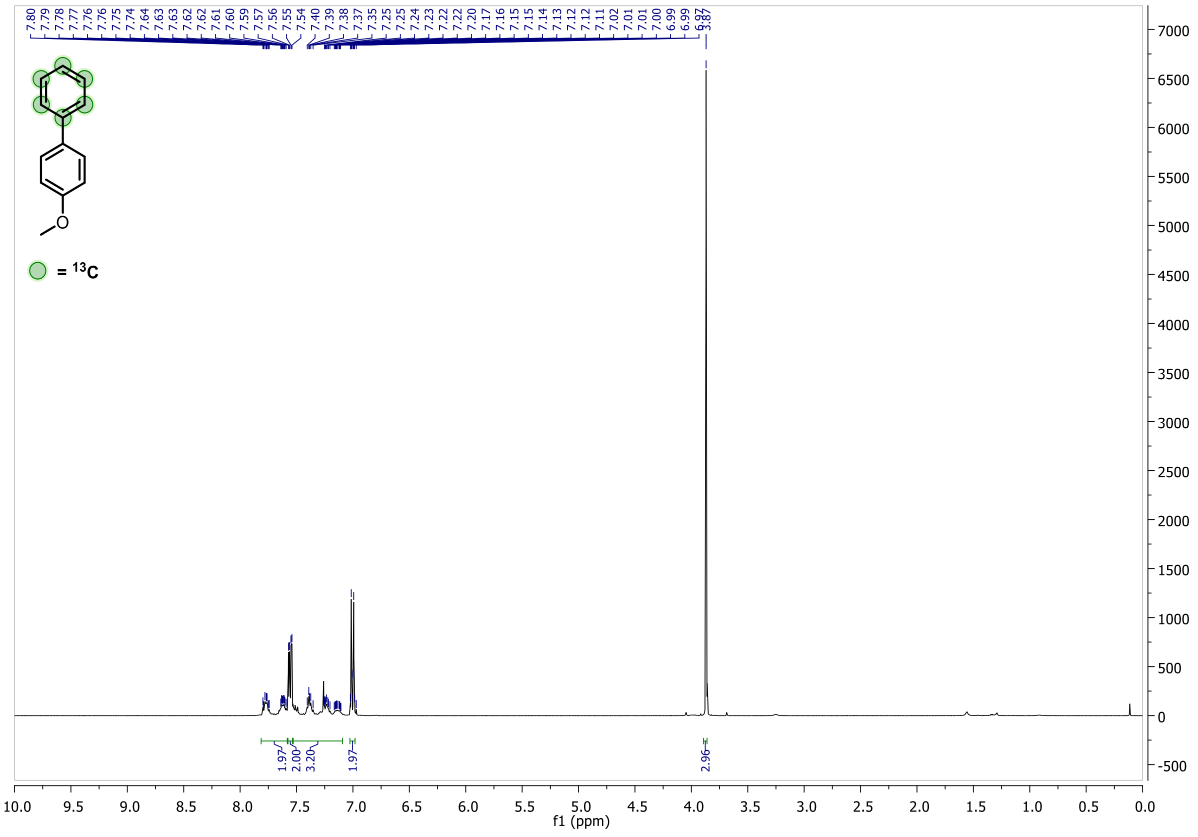

^{1}H NMR-{^{13}C}NMR (400 MHz, $CDCl_3$), 1-methoxy-4-(phenyl-$^{13}C_6$)benzene (**[$^{13}C_6$]3**)

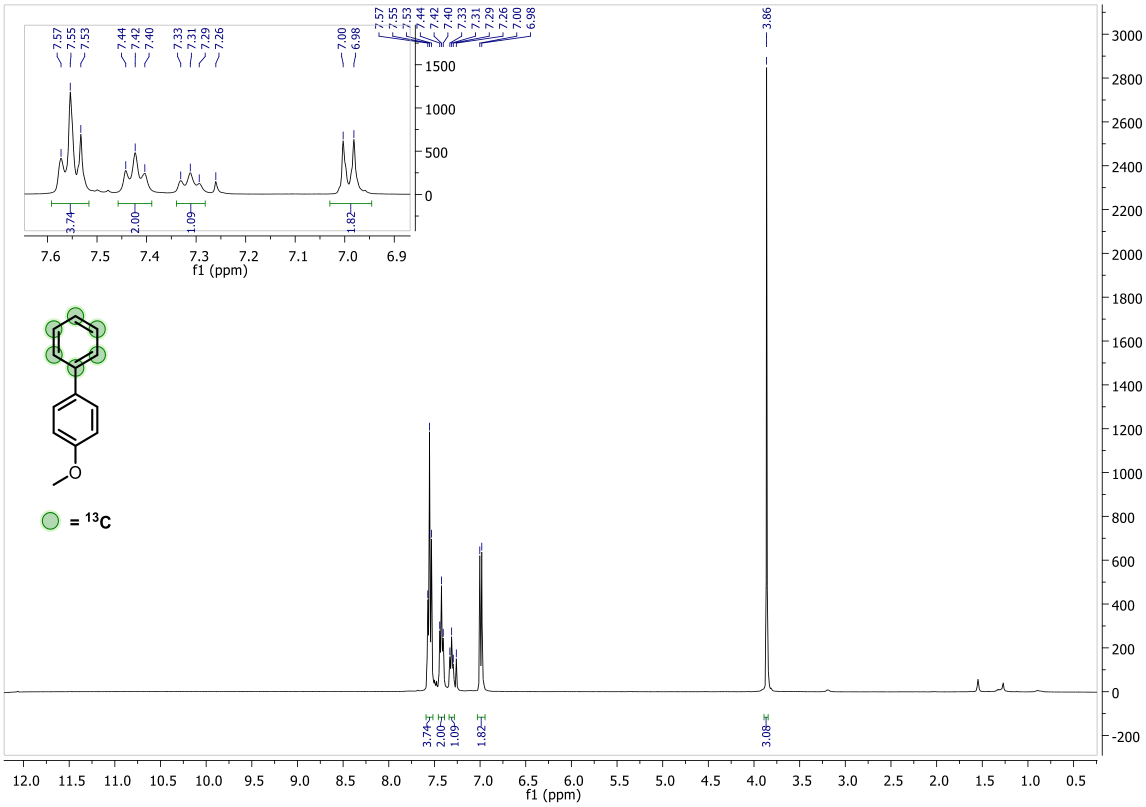

^{13}C NMR (100 MHz, $CDCl_3$), 1-methoxy-4-(phenyl-$^{13}C_6$)benzene (**[$^{13}C_6$]3**)

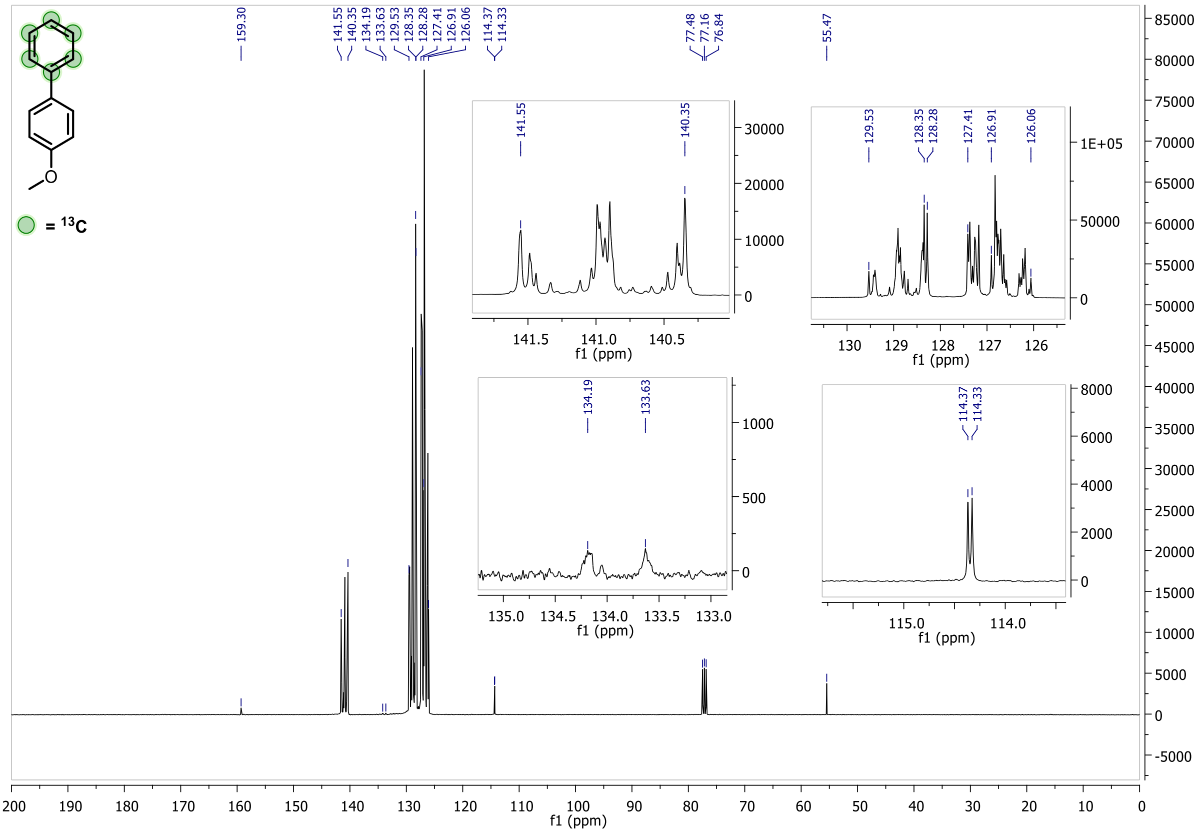

^{1}H NMR (400 MHz, $CDCl_3$), 3,4-dimethoxy-1,1'-biphenyl-2',3',4',5',6'-d_5 (**[$^2H_5$]4**)

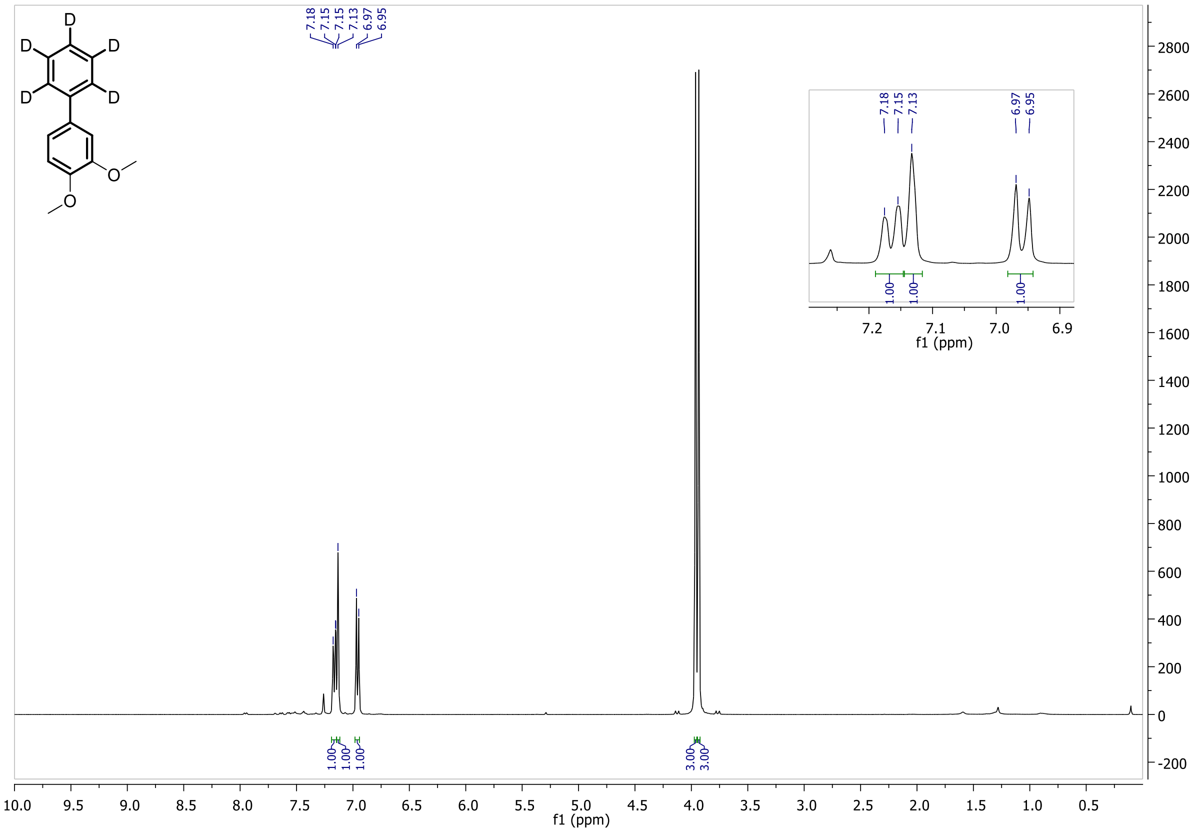

^{2}H NMR-{^{1}H}NMR (61 MHz, $CHCl_3$), 3,4-dimethoxy-1,1'-biphenyl-2',3',4',5',6'-d_5 (**[$^2H_5$]4)**

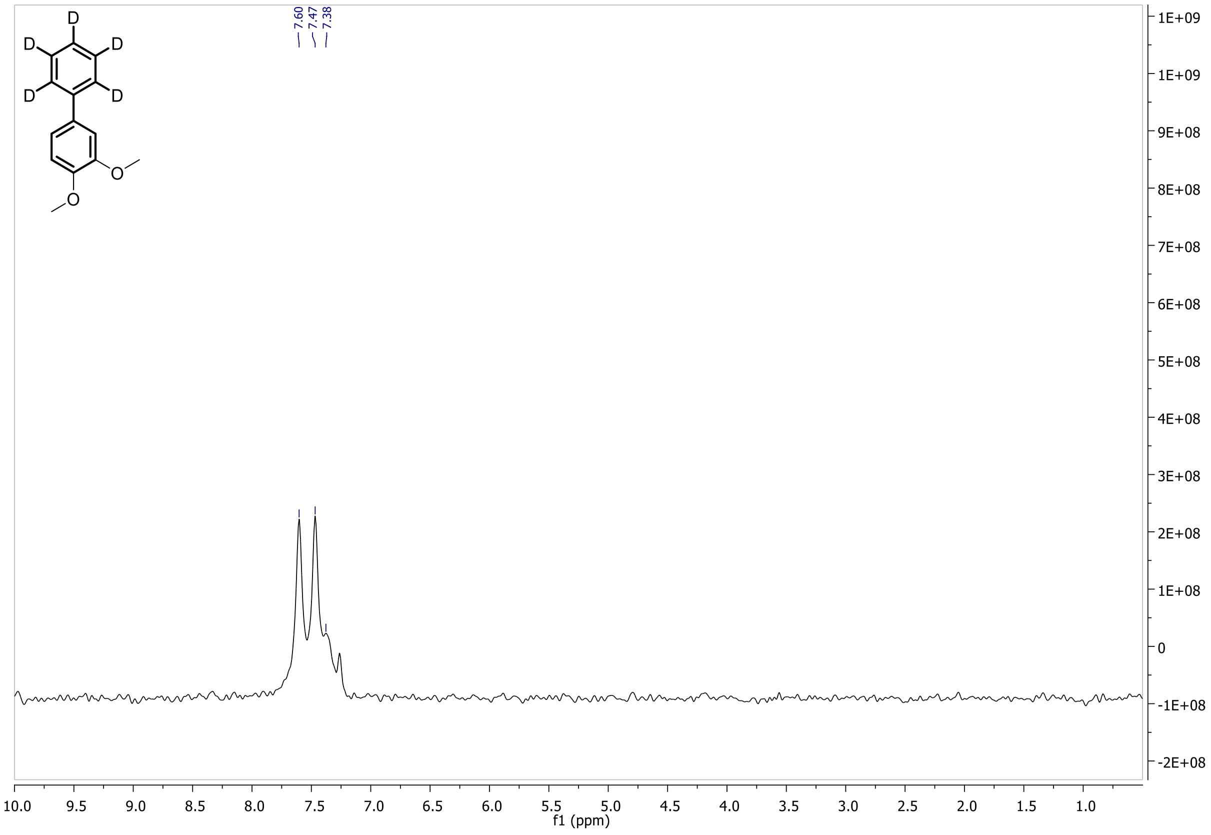

^{13}C NMR (100 MHz, $CDCl_3$), 3,4-dimethoxy-1,1'-biphenyl-2',3',4',5',6'-d_5 (**[$^2H_5$]4)**

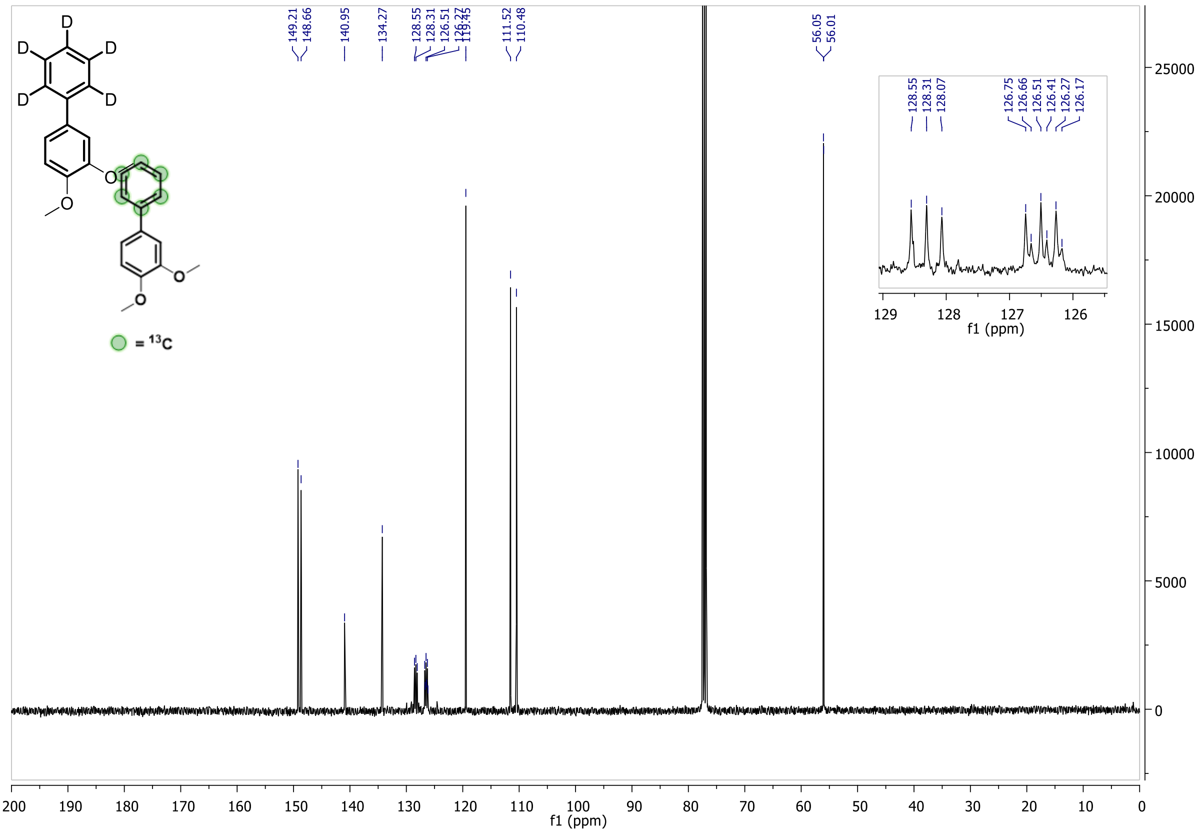

^1H NMR-{^{13}C}NMR (400 MHz, $CDCl_3$),1,2-dimethoxy-4-(phenyl-$^{13}C_6$) benzene (**[$^{13}C_6$]4)**

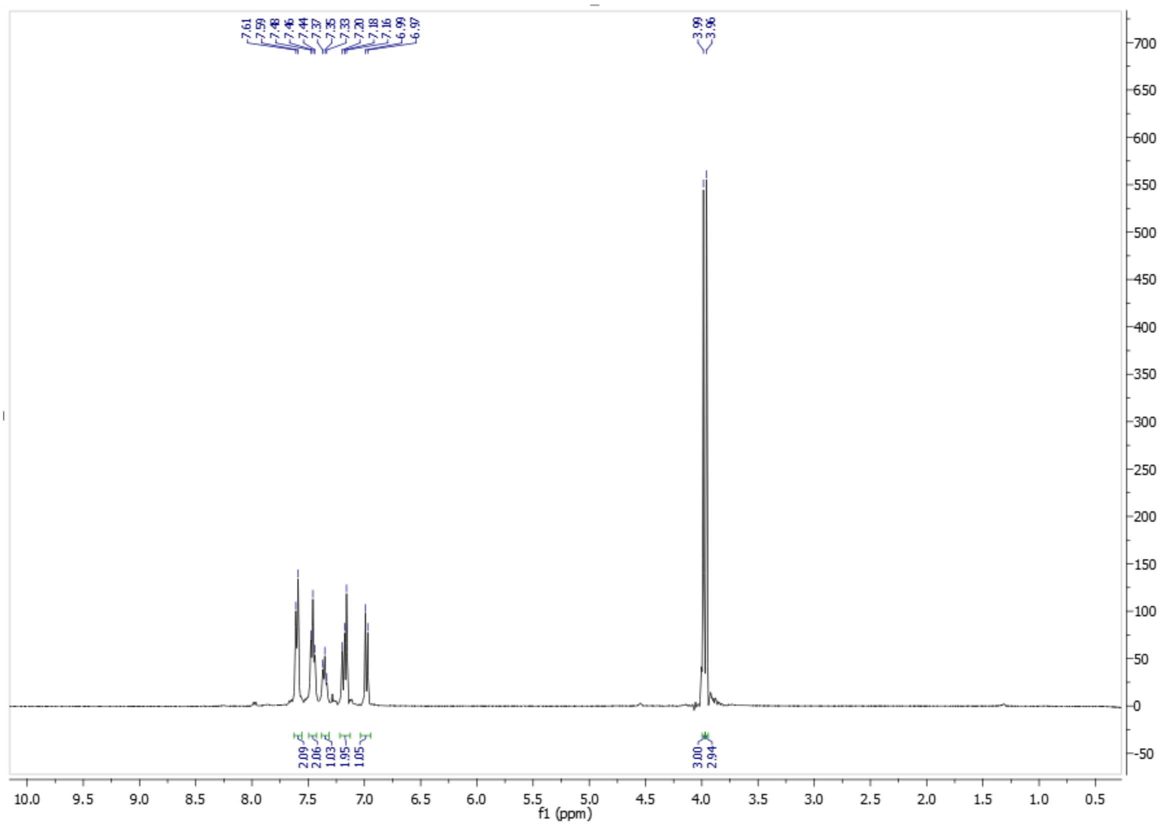

^{13}C NMR (100 MHz, $CDCl_3$), 1,2-dimethoxy-4-(phenyl-$^{13}C_6$) benzene (**[$^{13}C_6$]4**)

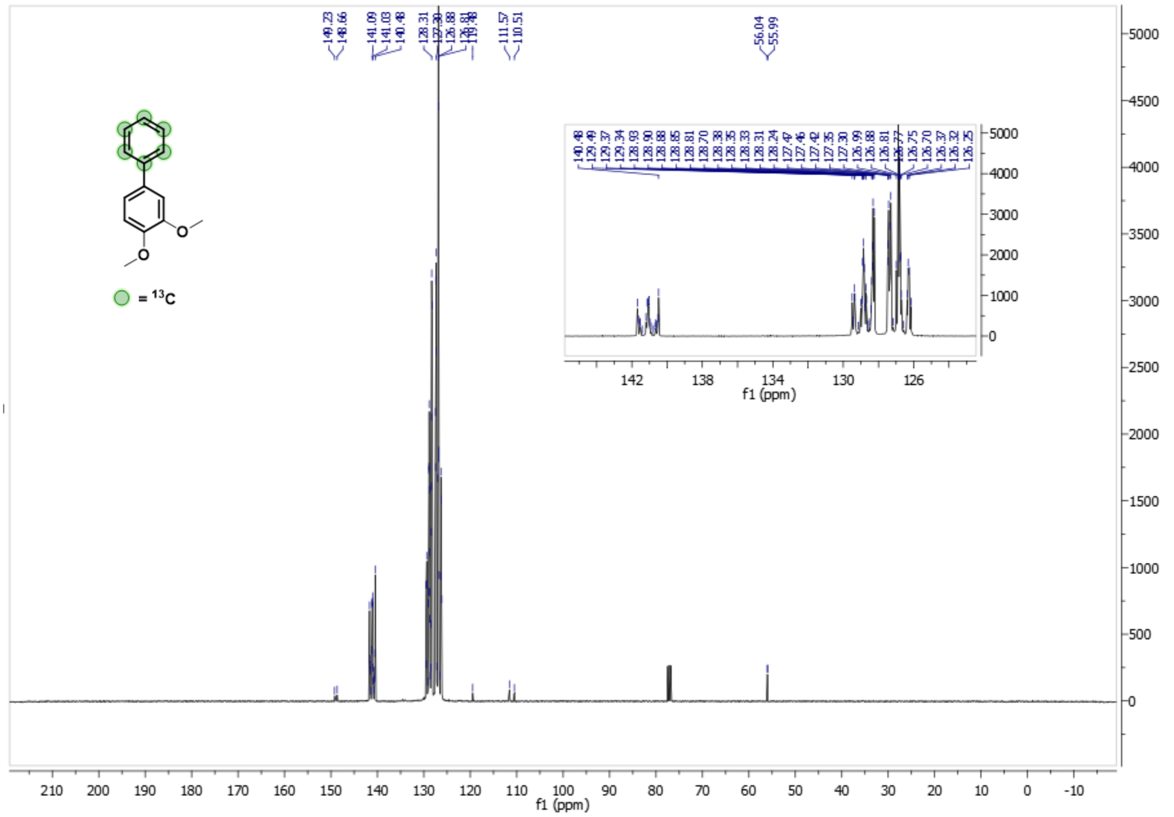

^{1}H NMR (400 MHz, $CDCl_3$), 1-(tert-butyl)-4-(phenyl-$^{13}C_6$)benzene (**[$^{13}C_6$]5**)

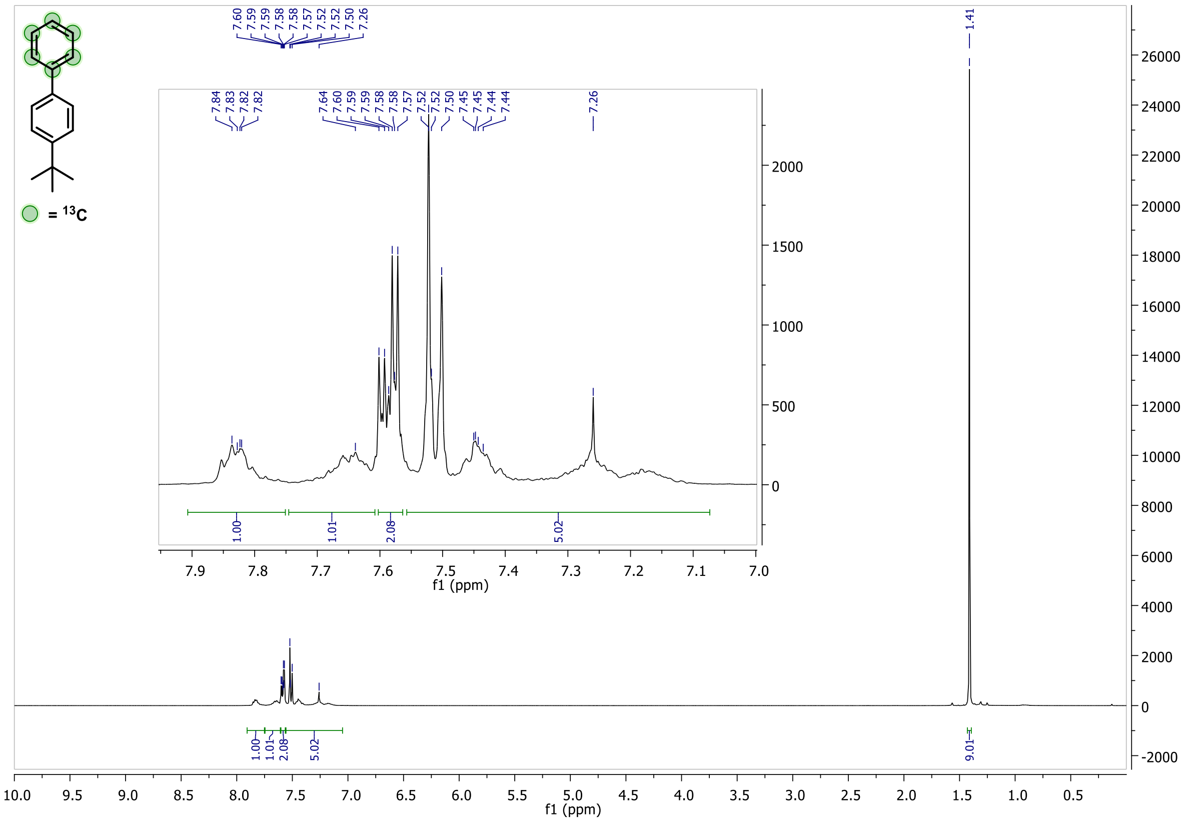

^{2}H NMR-{^{1}H}NMR (61 MHz, $CDCl_3$), 1-(tert-butyl)-4-(phenyl-$^{13}C_6$)benzene (**[^{13}C$_6$]5**)

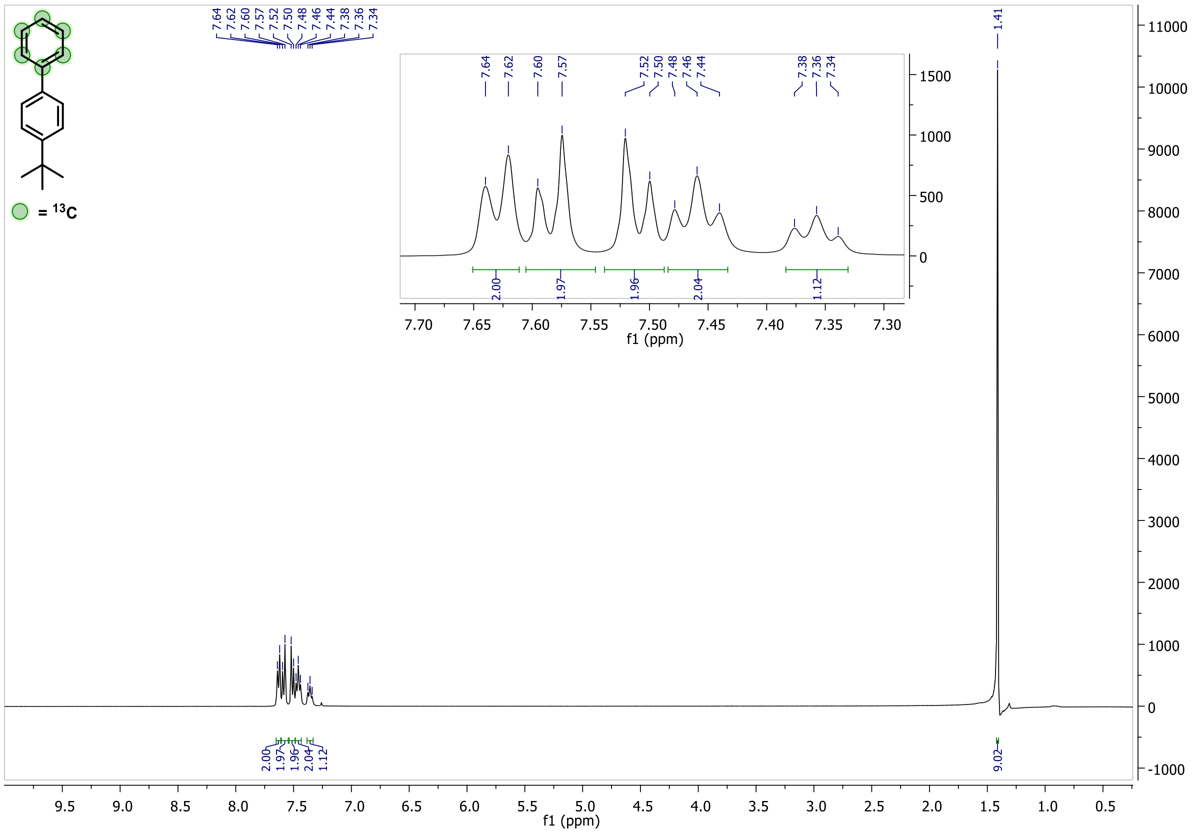

^{13}C NMR (100 MHz, $CDCl_3$), 1-(tert-butyl)-4-(phenyl-$^{13}C_6$)benzene (**[^{13}C$_6$]5**)

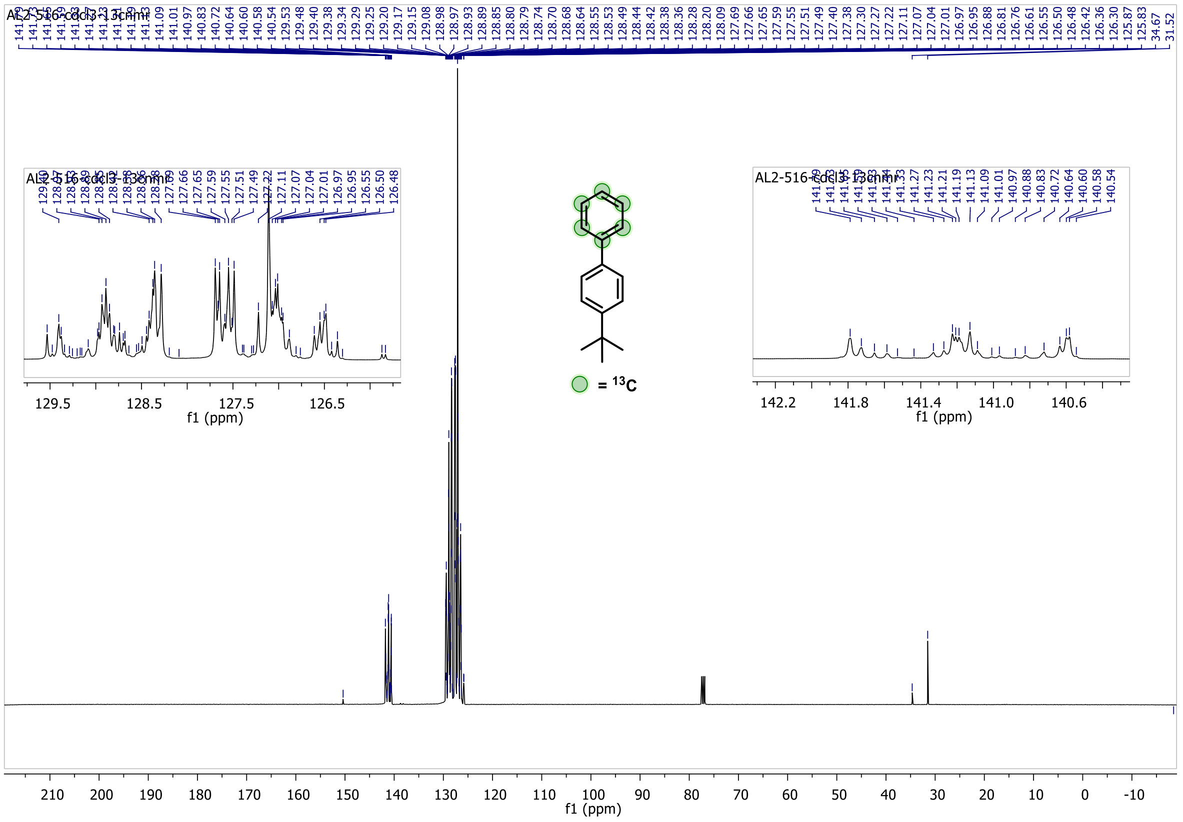

^{1}H NMR (400 MHz, $CDCl_3$), 4-chloro-1,1'-biphenyl-2',3',4',5',6'-d_5 (**[^{2}H$_5$]6**)

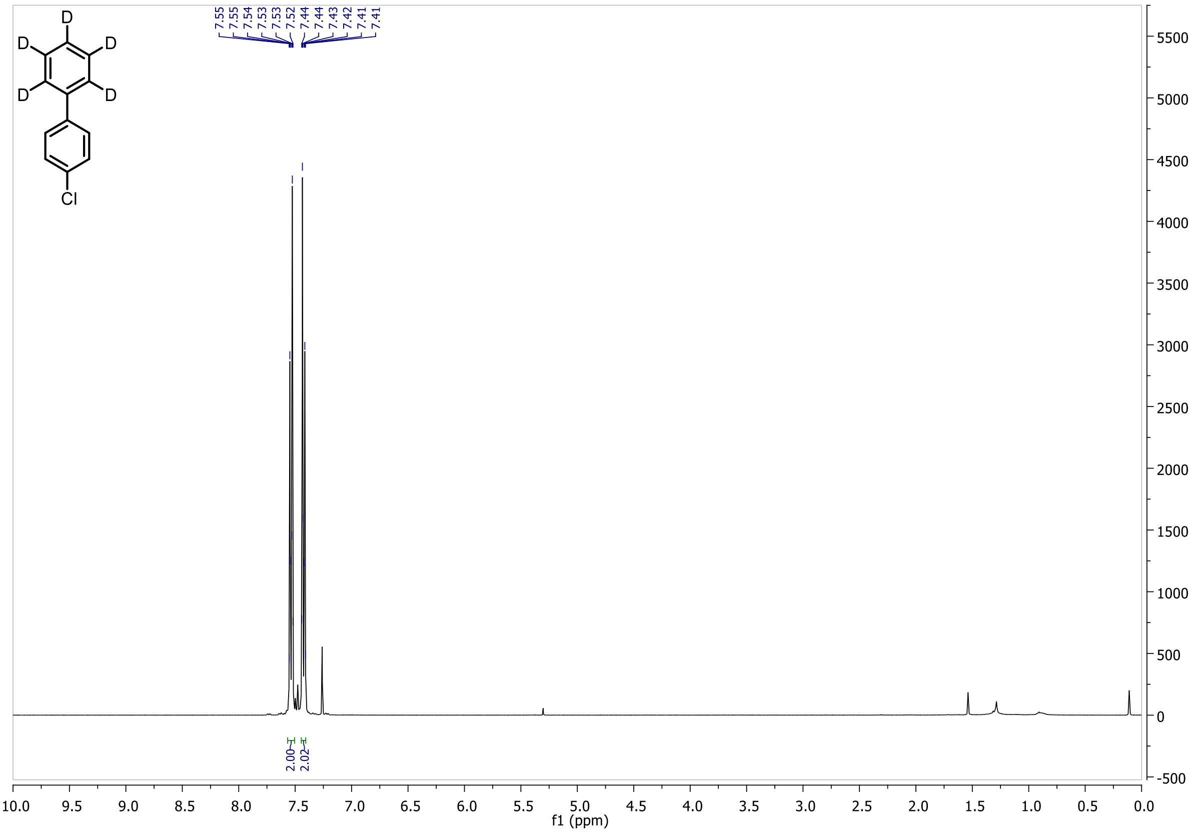

^{2}H NMR-{^{1}H}NMR (61 MHz, $CHCl_3$), 4-chloro-1,1'-biphenyl-2',3',4',5',6'-d_5 (**[^{2}H$_5$]6**)

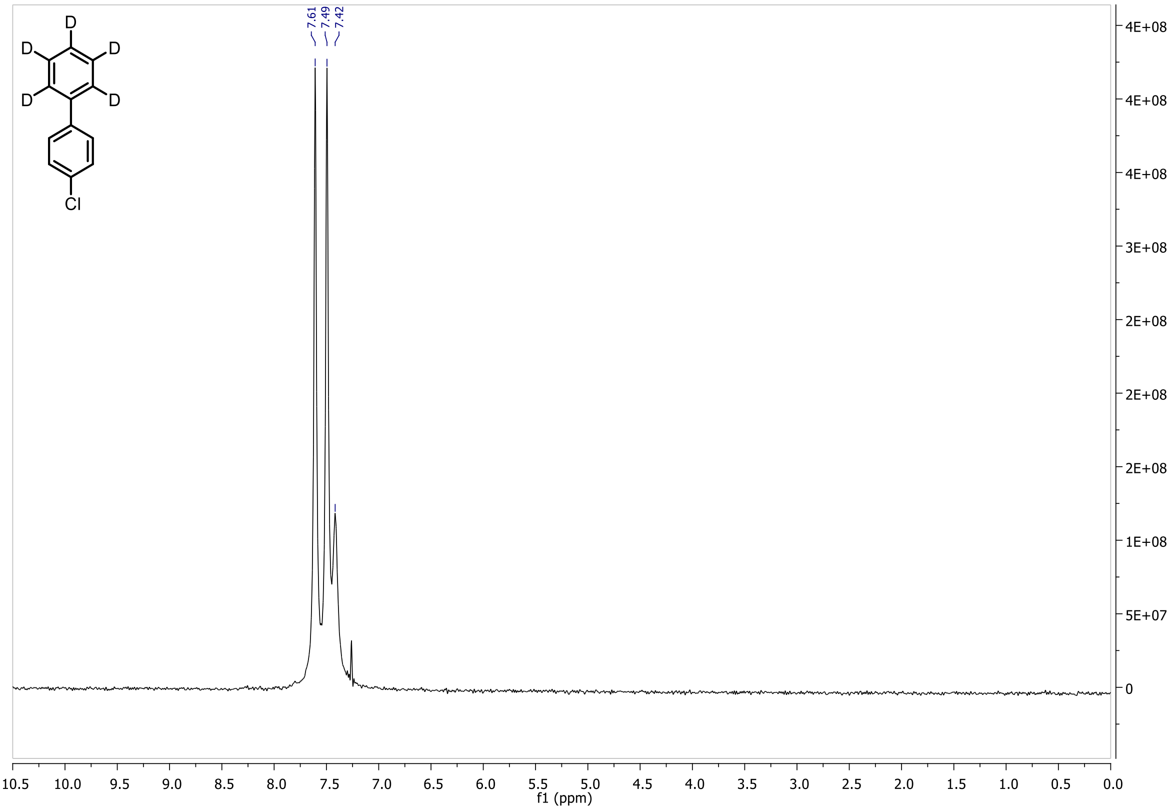

^{13}C NMR (100 MHz, $CDCl_3$), 4-chloro-1,1'-biphenyl-2',3',4',5',6'-d_5 (**[^{2}H$_5$]6**)

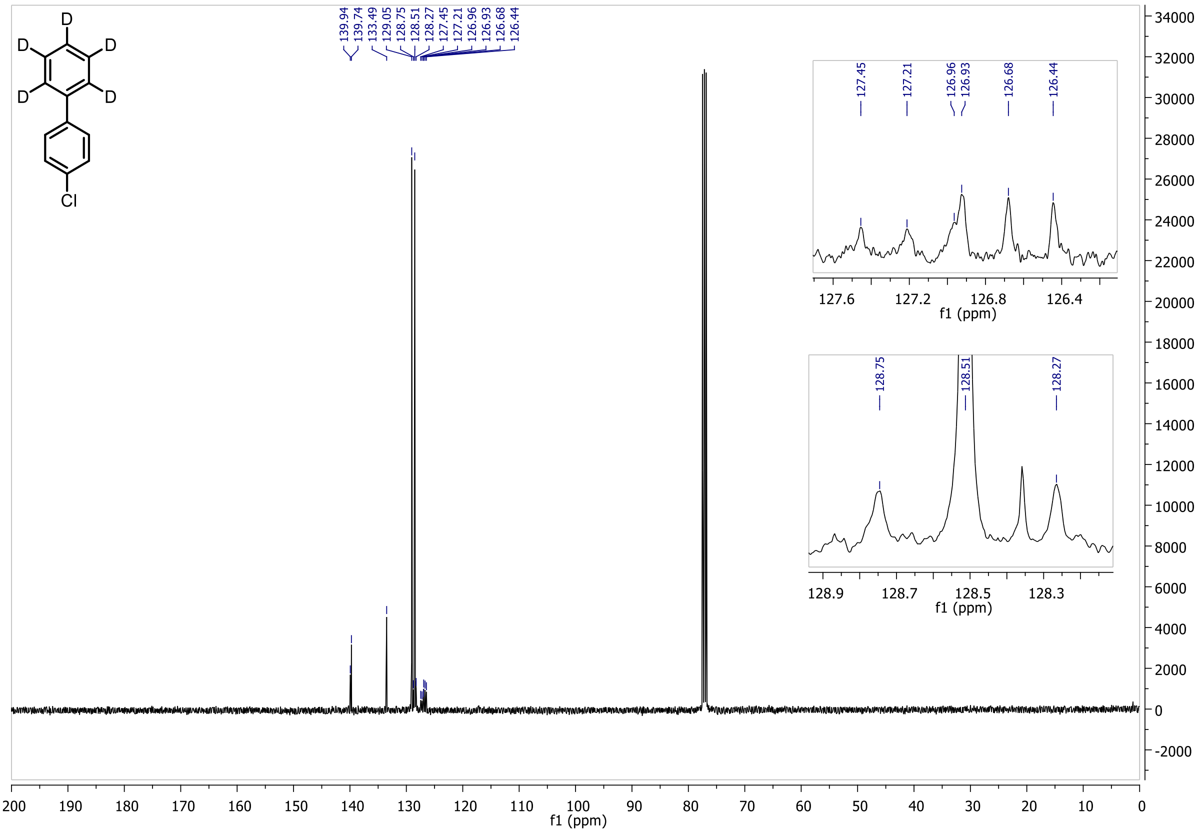

^{1}H NMR (400 MHz, $CDCl_3$), [1,1'-biphenyl]-4-carbonitrile-2',3',4',5',6'-d_5 (**[^{2}H$_5$]7**)

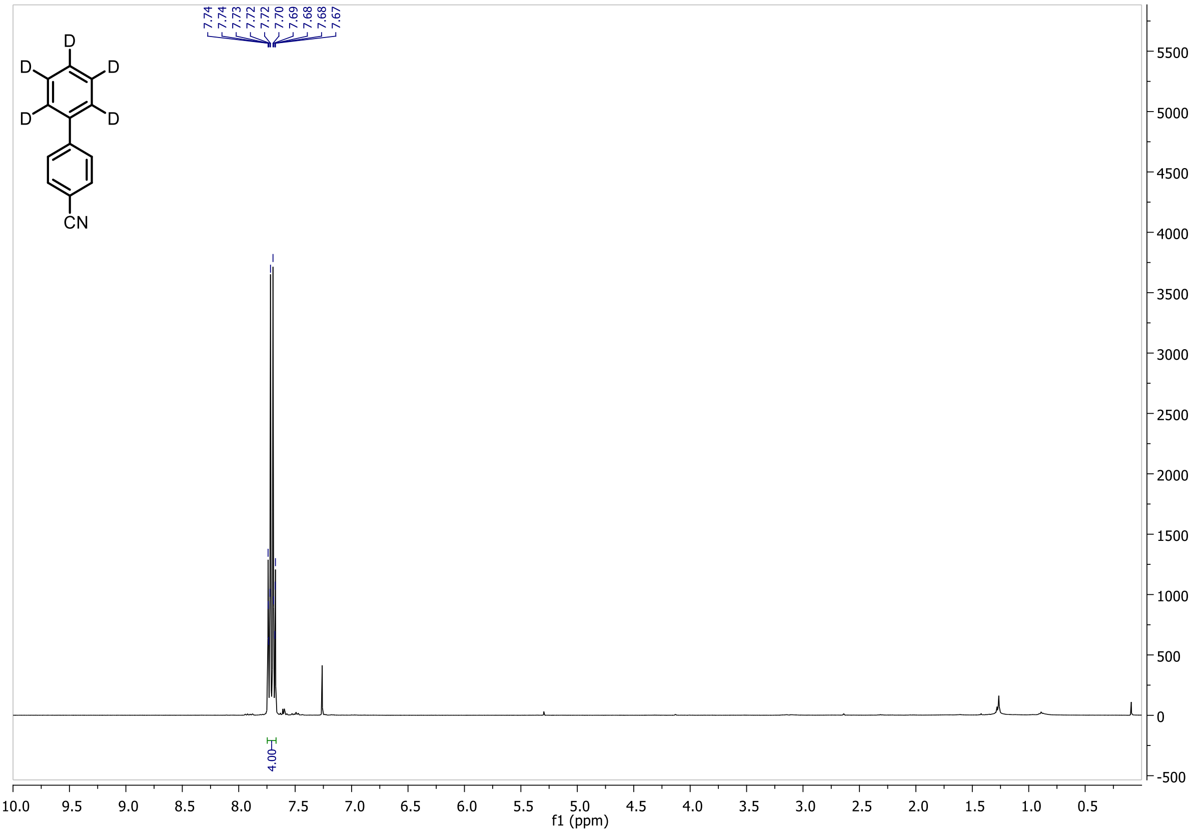

^{2}H NMR-{^{1}H}NMR (61 MHz, $CHCl_3$), [1,1'-biphenyl]-4-carbonitrile-2',3',4',5',6'-d_5 (**[^{2}H$_5$]7**)

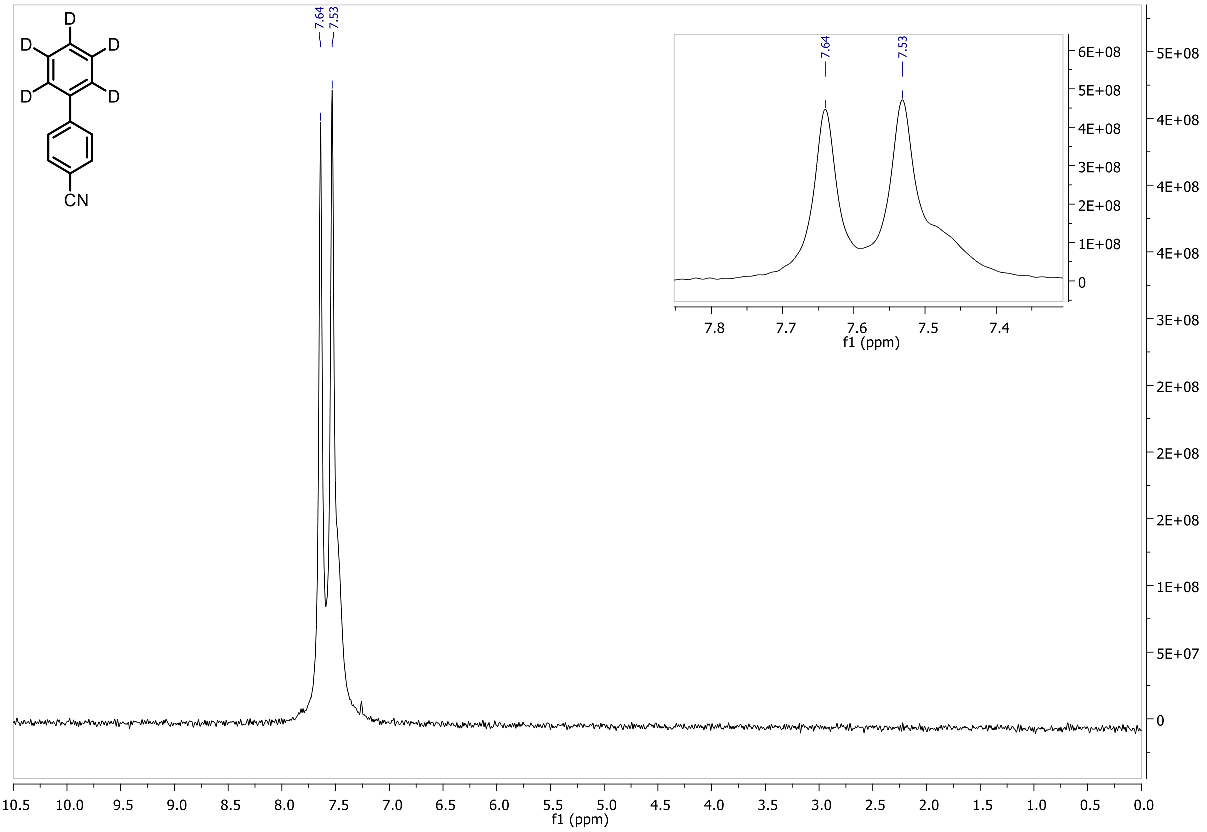

^{13}C NMR (100 MHz, $CDCl_3$), [1,1'-biphenyl]-4-carbonitrile-2',3',4',5',6'-d_5 (**[^{2}H$_5$]7**)

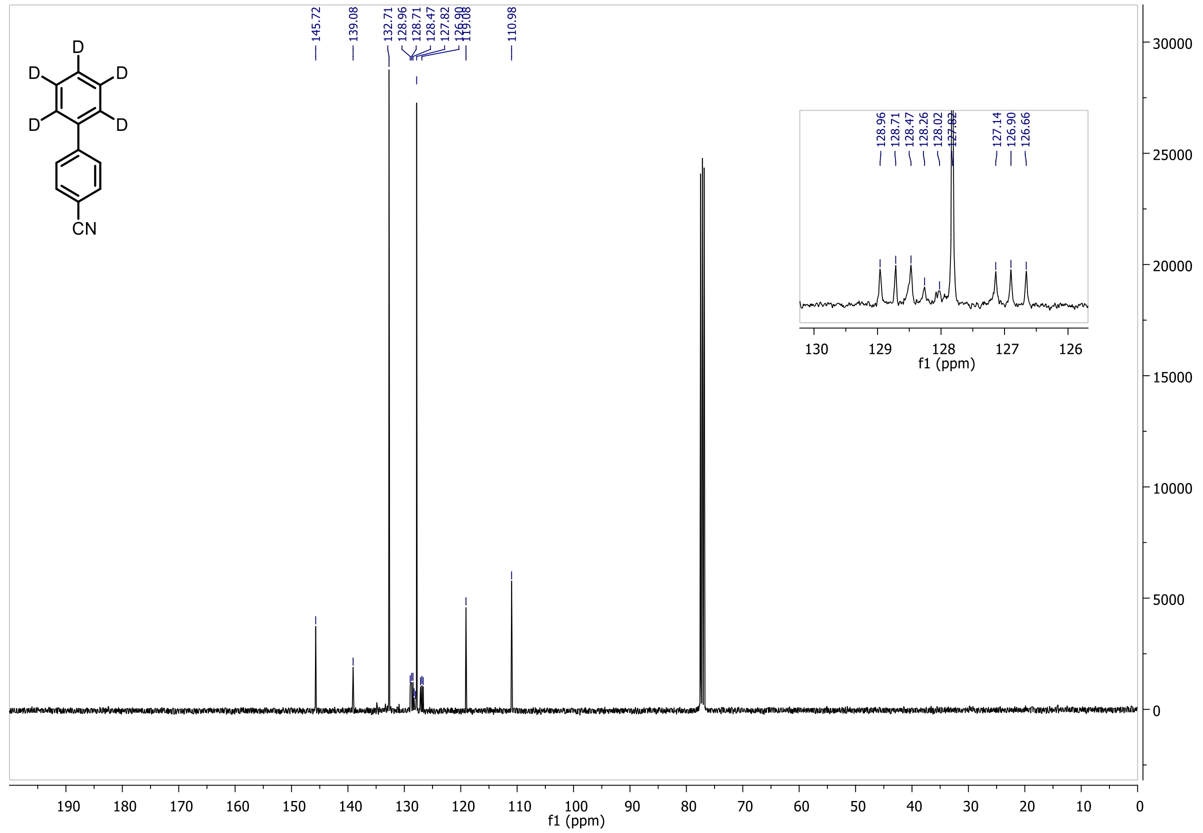

^{1}H NMR (400 MHz, MeOD), 4-(phenyl-$^{13}C_6$)benzoic acid (**[^{13}C$_6$]8**)

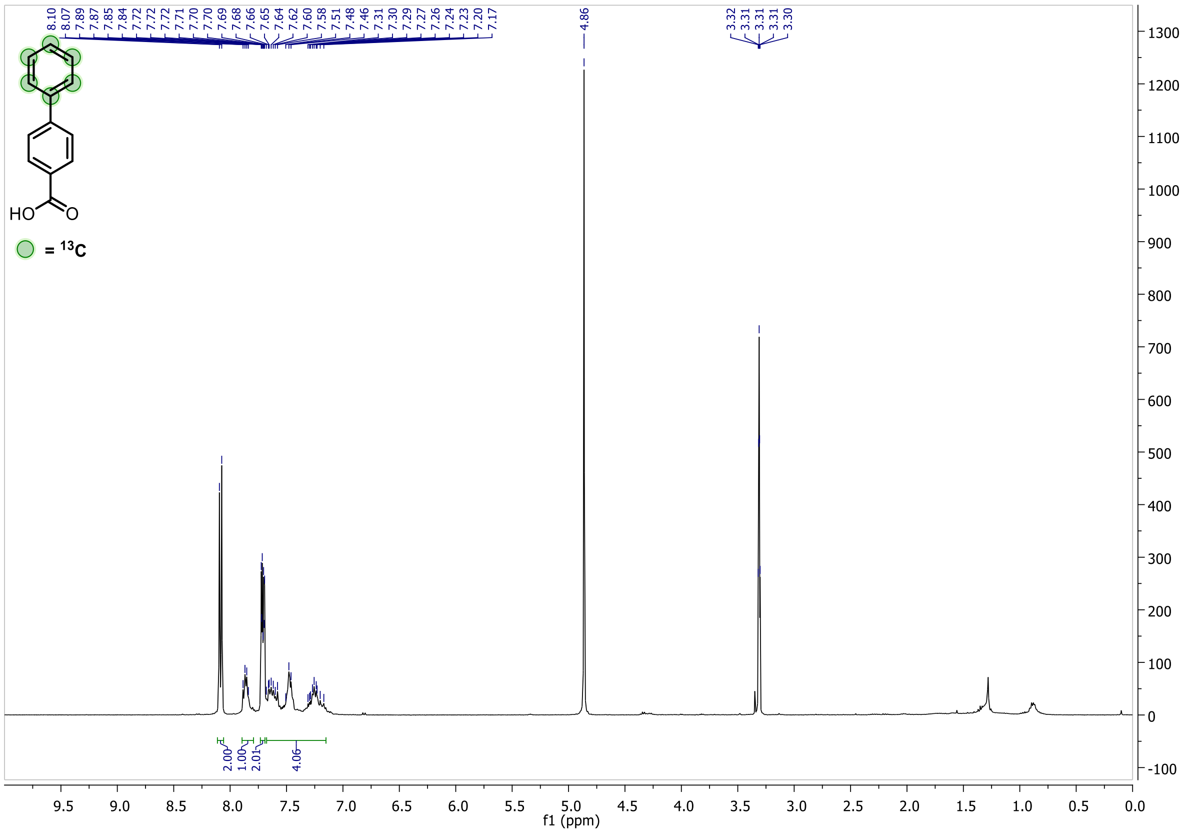

^{1}H NMR-{^{13}C}NMR (400 MHz, DMF-d_7), 4-(phenyl-$^{13}C_6$)benzoic acid (**[^{13}C$_6$]8**)

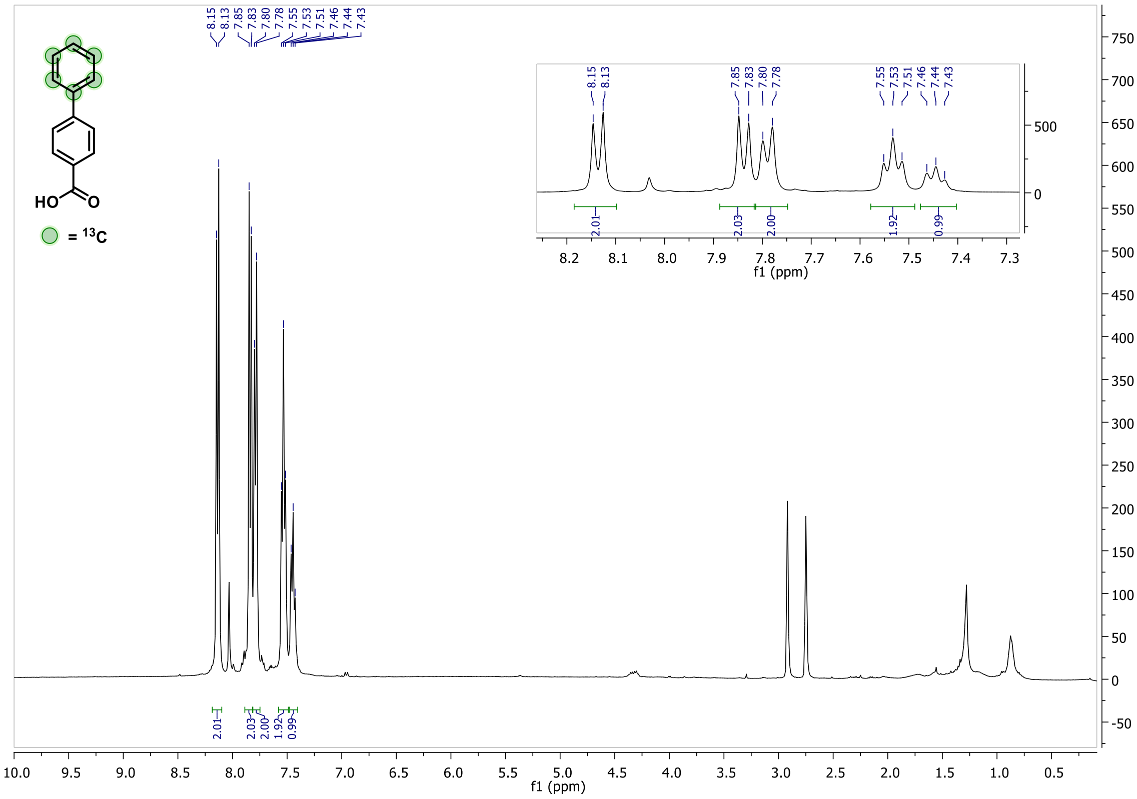

^{13}C NMR (100 MHz, MeOD), 4-(phenyl-$^{13}C_6$)benzoic acid (**[^{13}C$_6$]8**)

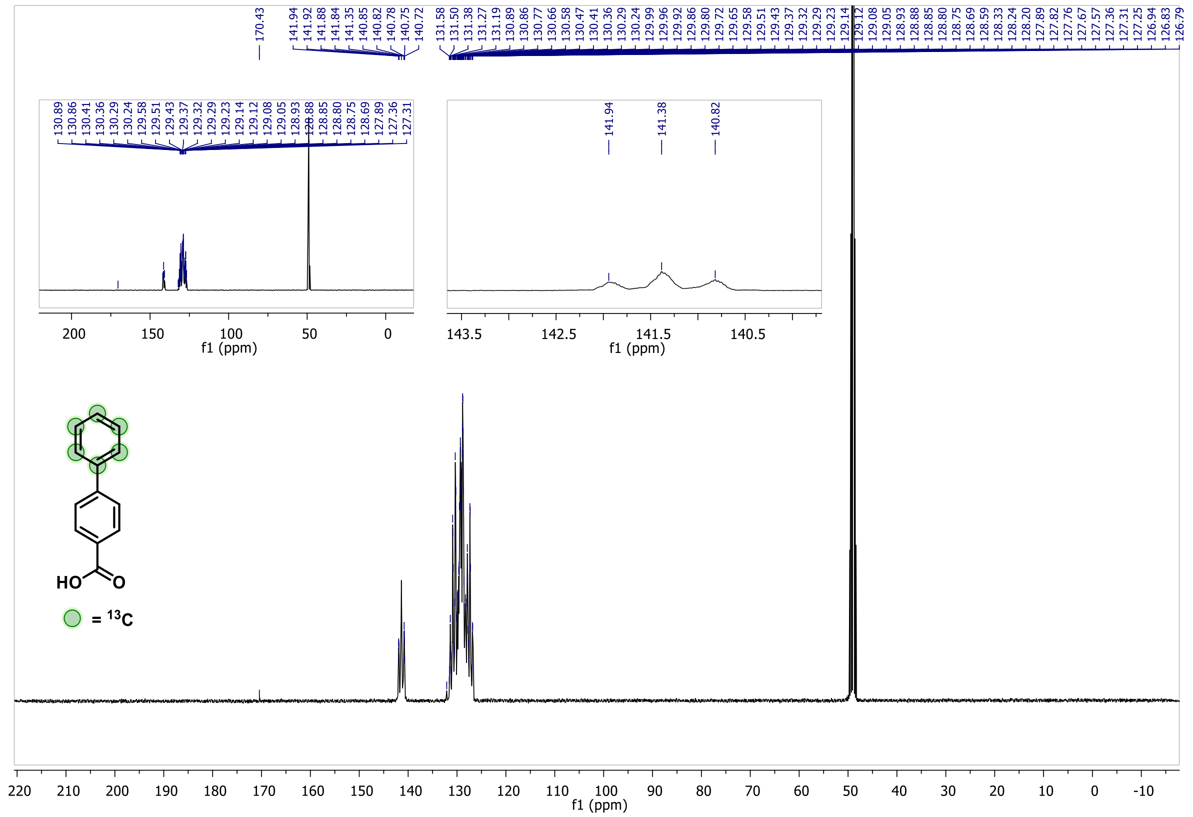

^{1}H NMR (400 MHz, CDCl$_3$), phenyl(4-(phenyl-^{13}C$_6$)phenyl)methanone (**[^{13}C$_6$]9)**

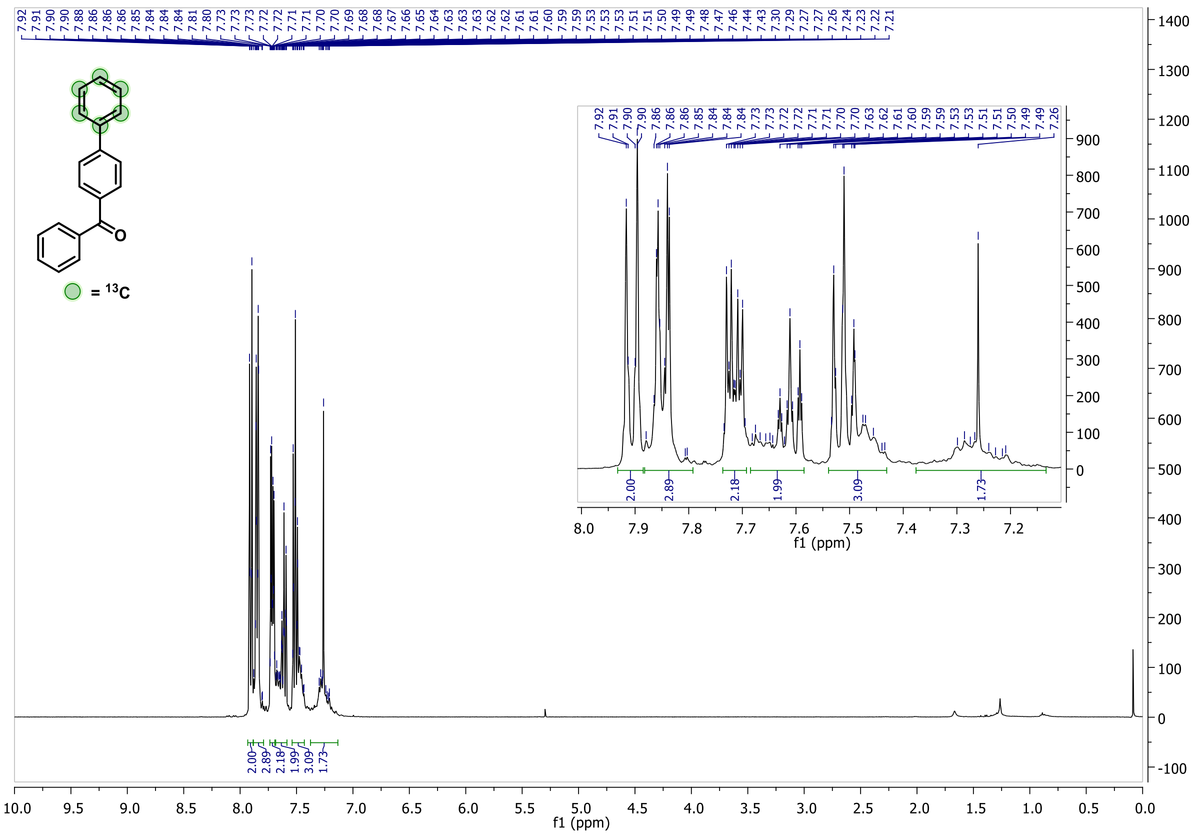

^{1}H NMR-{^{13}C}NMR (400 MHz, $CDCl_3$), phenyl(4-(phenyl-$^{13}C_6$)phenyl)methanone (**[$^{13}C_6$]9)**

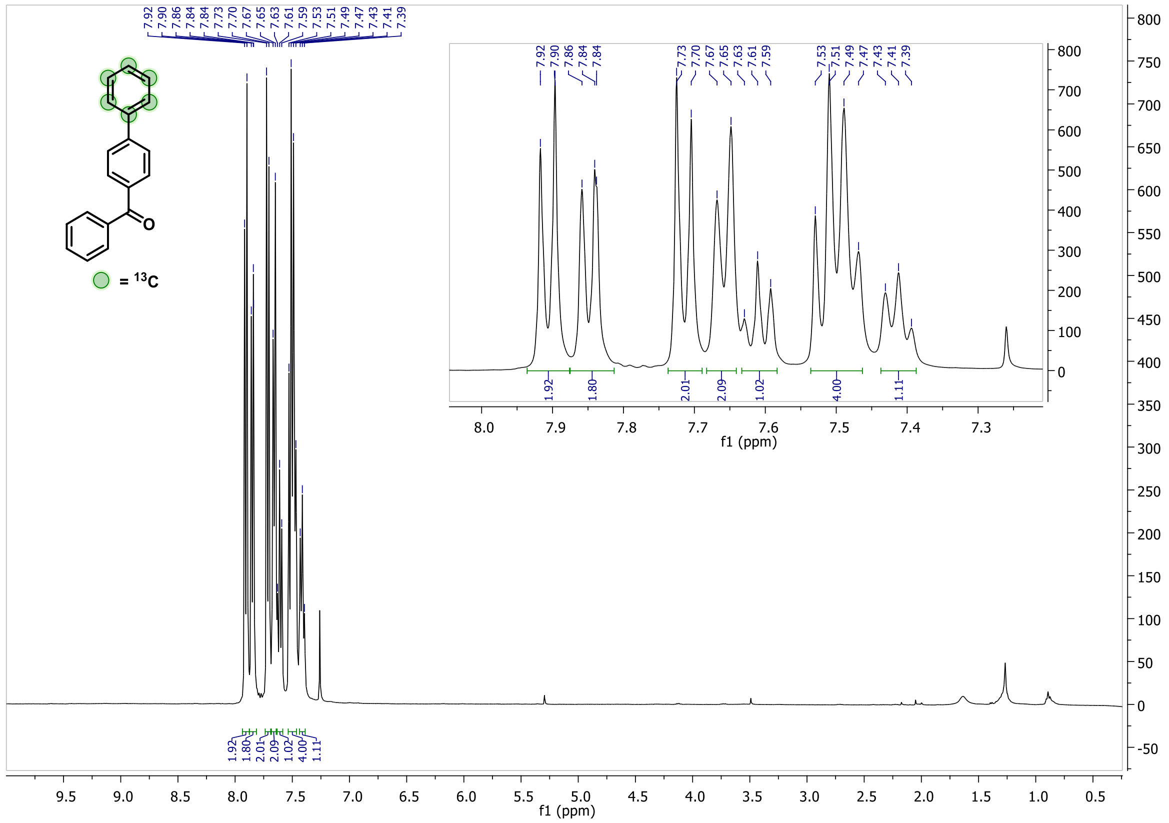

^{13}C NMR (100 MHz, $CDCl_3$), phenyl(4-(phenyl-$^{13}C_6$)phenyl)methanone (**[$^{13}C_6$]9)**

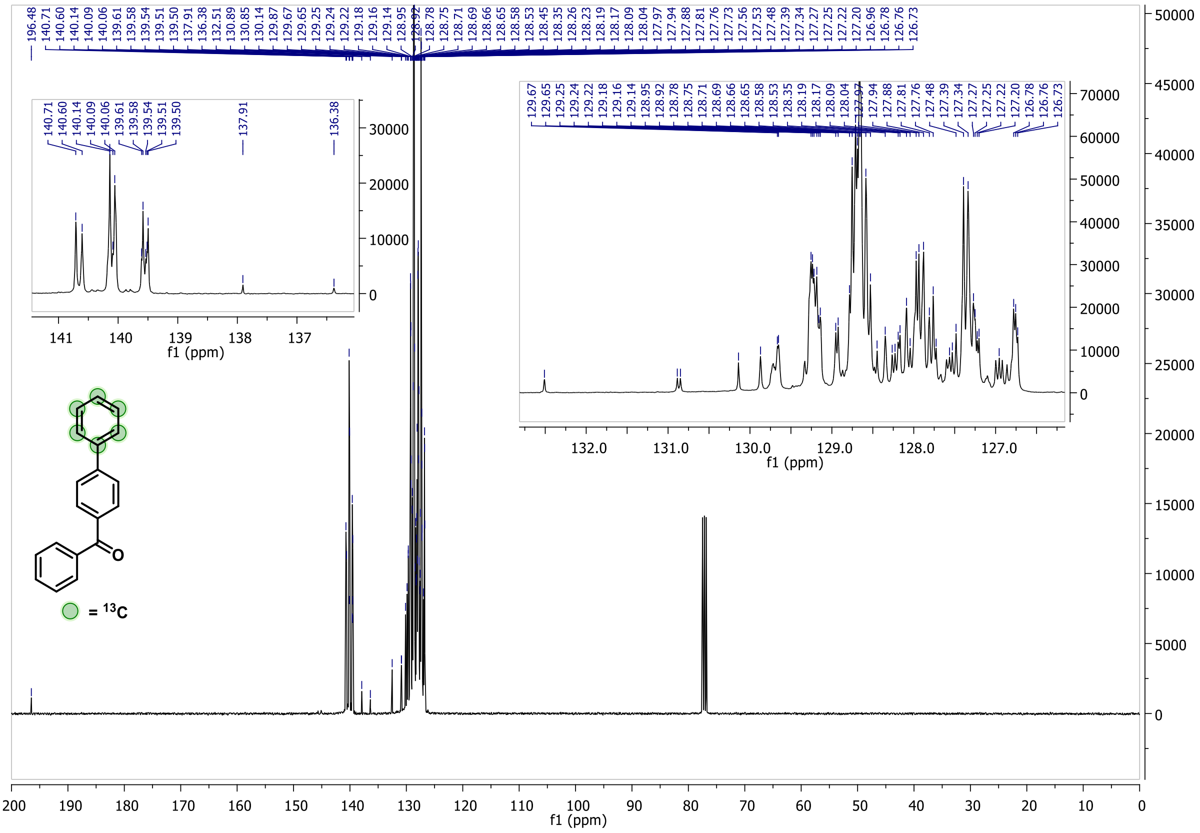

^{1}H NMR (400 MHz, $CDCl_3$), 2-fluoro-1,1'-biphenyl-2',3',4',5',6'-d_5 (**[$^2H_5$]10**)

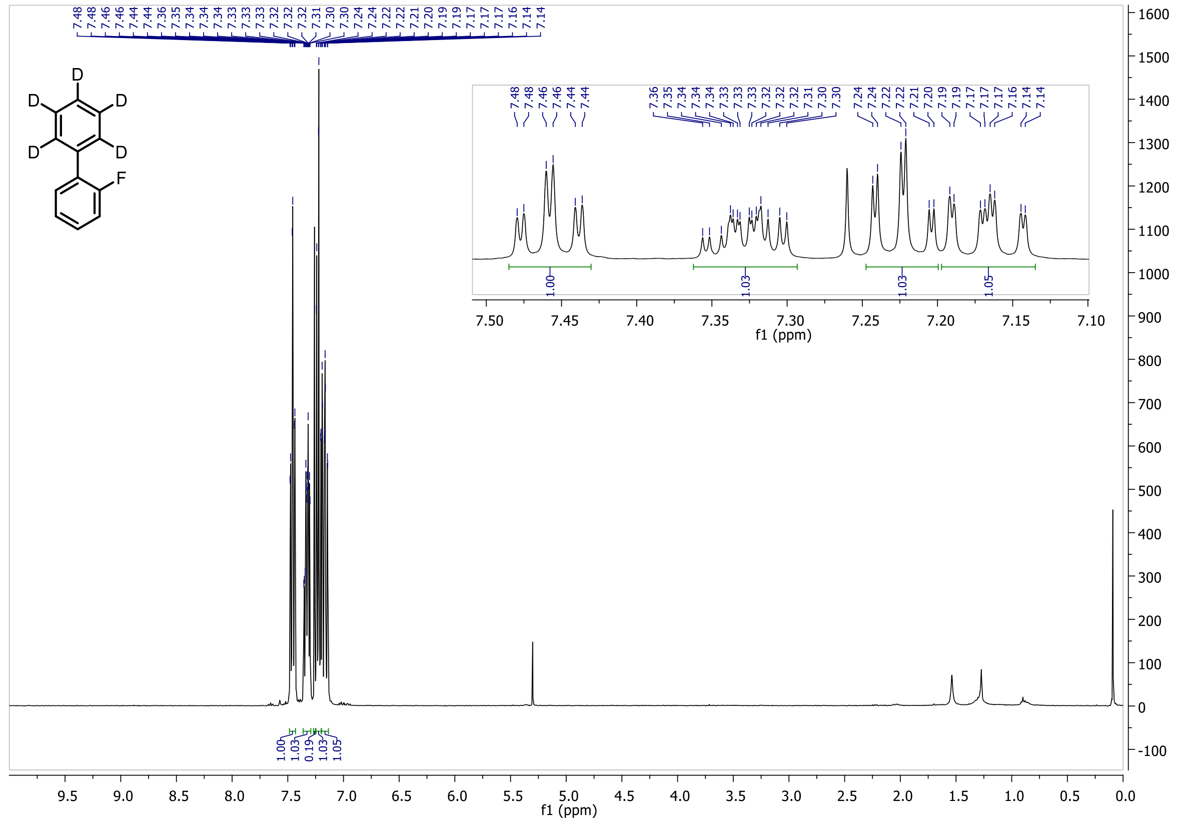

^{2}H NMR-{^{1}H}NMR (61 MHz, $CHCl_3$), 2-fluoro-1,1'-biphenyl-2',3',4',5',6'-d_5 (**[$^2H_5$]10**)

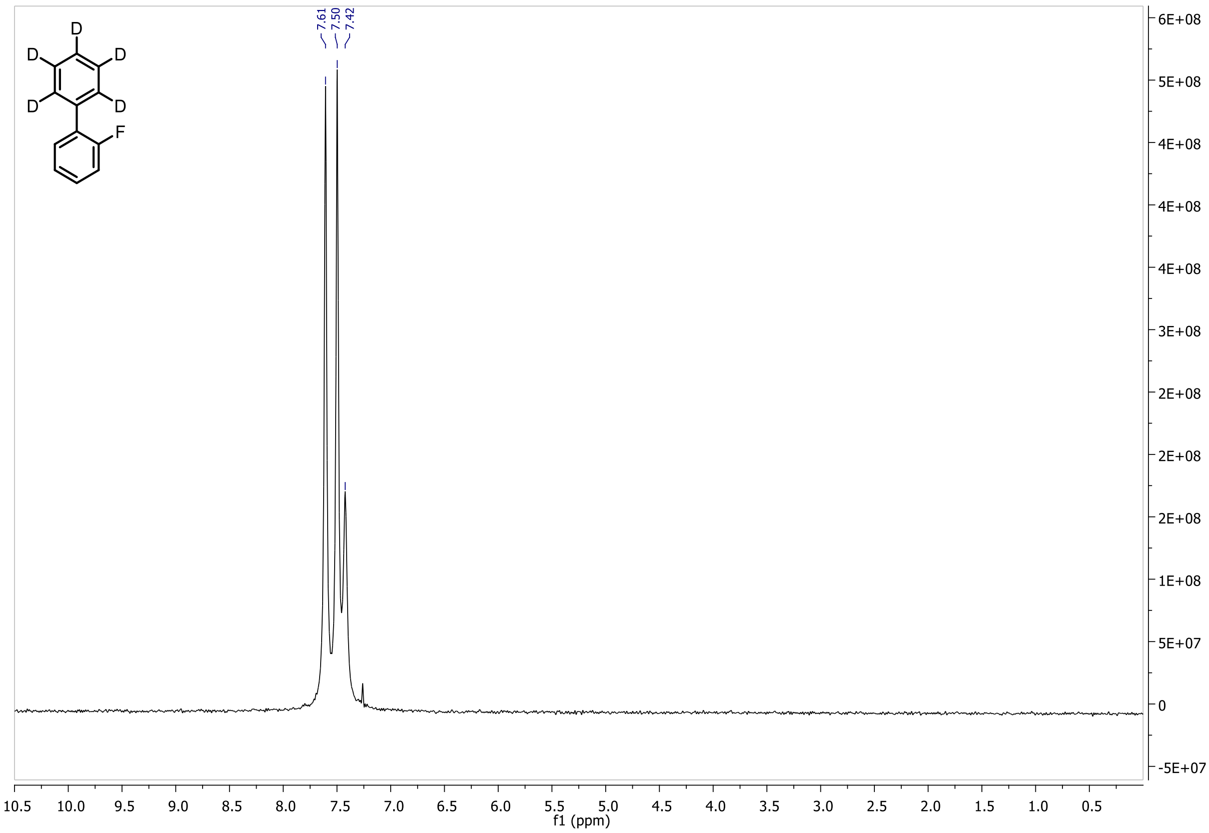

^{13}C NMR (100 MHz, CDCl$_3$), 2-fluoro-1,1'-biphenyl-2',3',4',5',6'-d_5 (**[^{2}H$_5$]10**) :

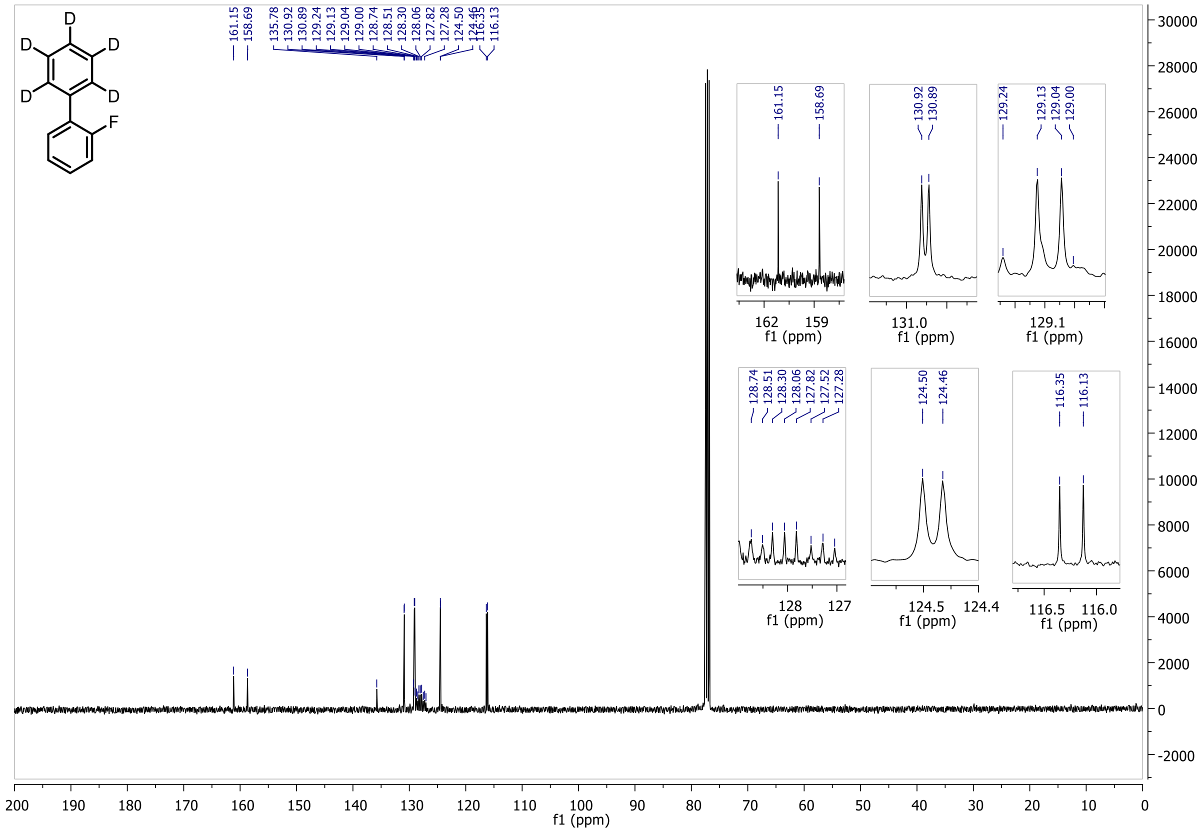

^{1}H NMR (400 MHz, CDCl$_3$), 2-(phenyl-^{13}C$_6$)-1,1'-biphenyl (**[^{13}C$_6$]11**)

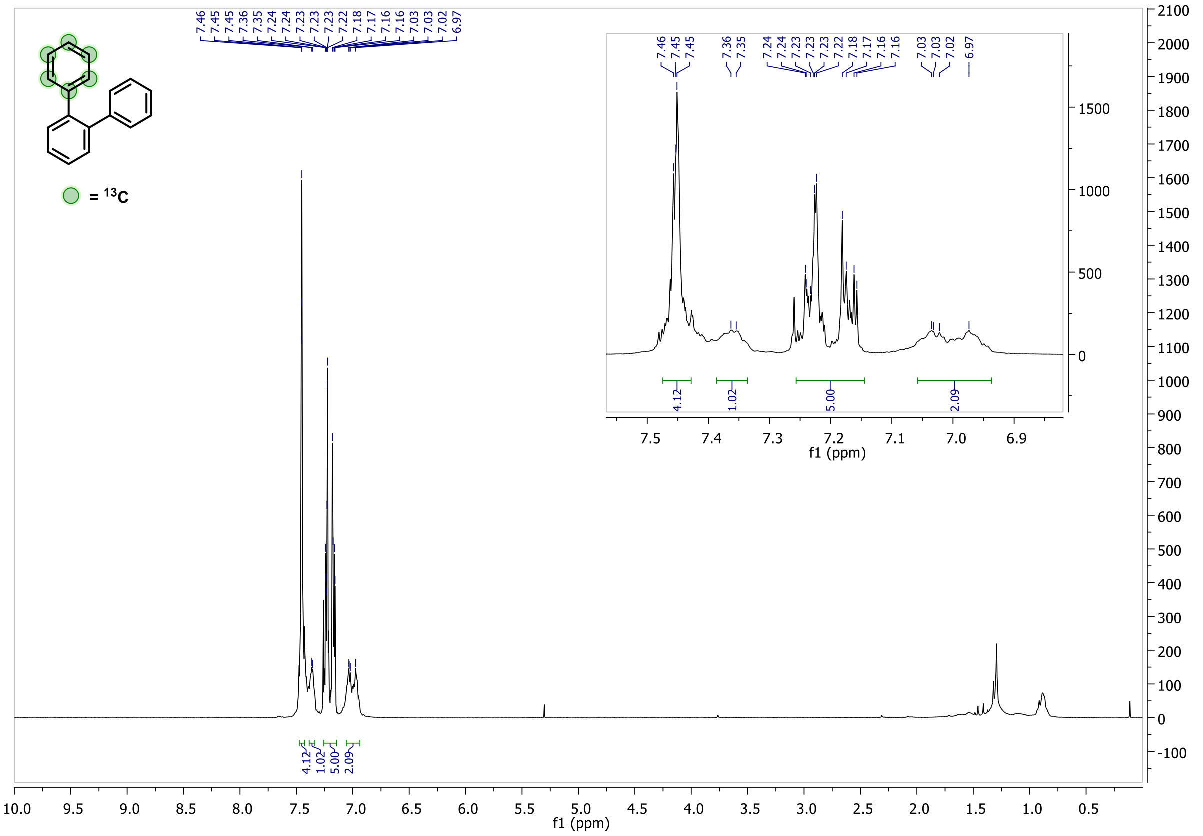

^{1}H NMR-{^{13}C}NMR (400 MHz, $CDCl_3$), 2-(phenyl-$^{13}C_6$)-1,1'-biphenyl (**[$^{13}C_6$]11**)

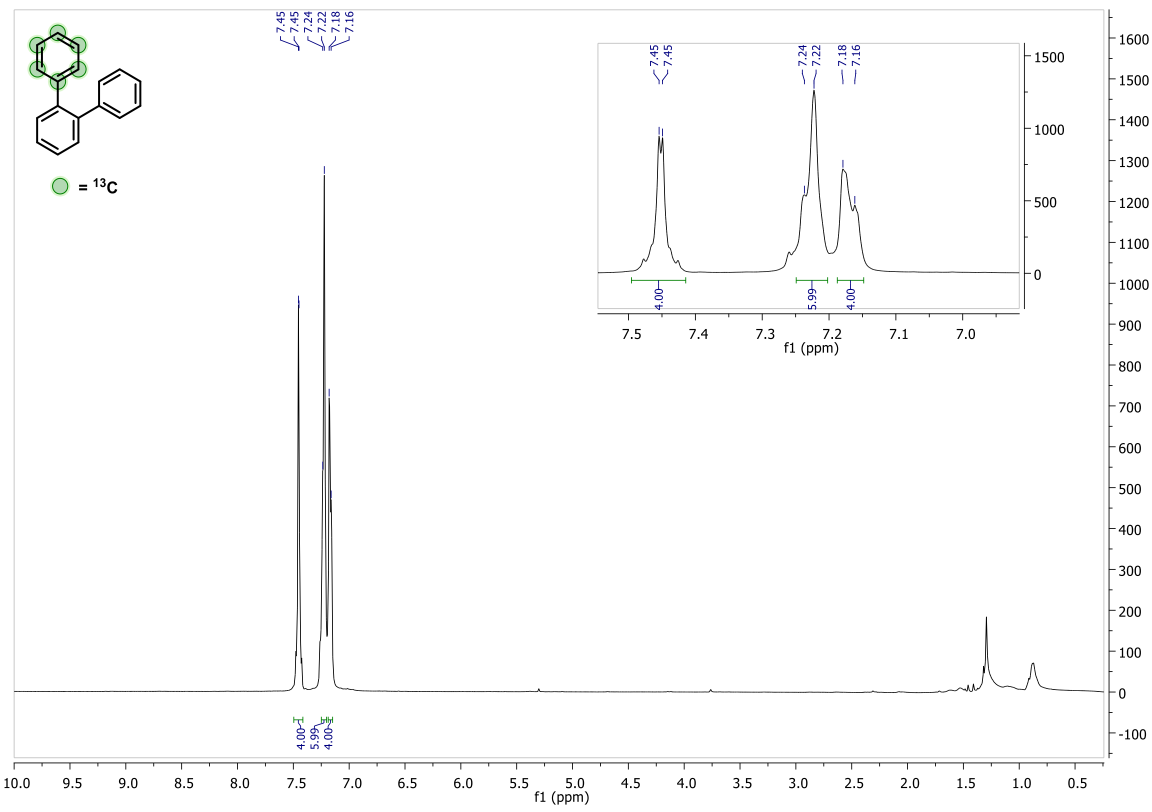

^{13}C NMR (100 MHz, $CDCl_3$), 2-(phenyl-$^{13}C_6$)-1,1'-biphenyl (**[$^{13}C_6$]11**)

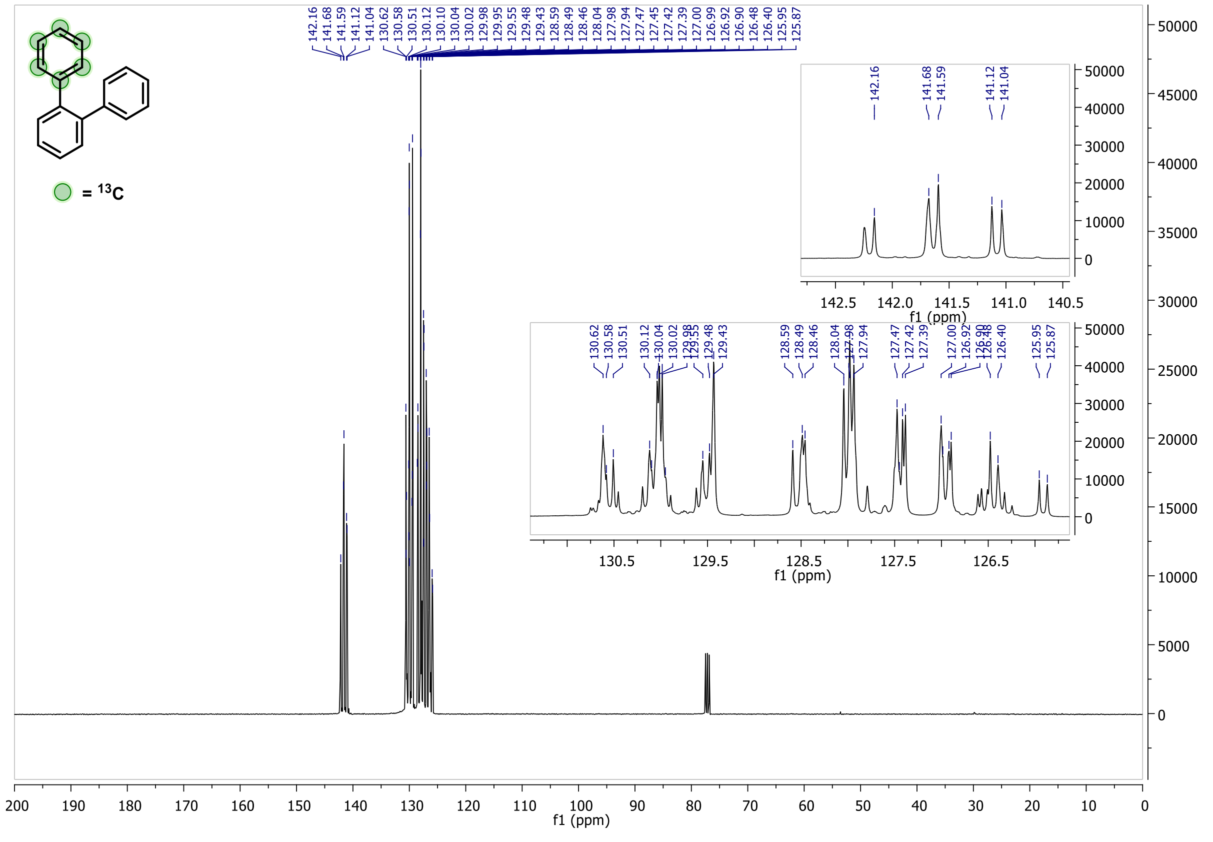

^{1}H NMR (400 MHz, $CDCl_3$), 1,1'-biphenyl-2,3,4,5,6-d_5 (**[^{2}H$_5$]12**) :

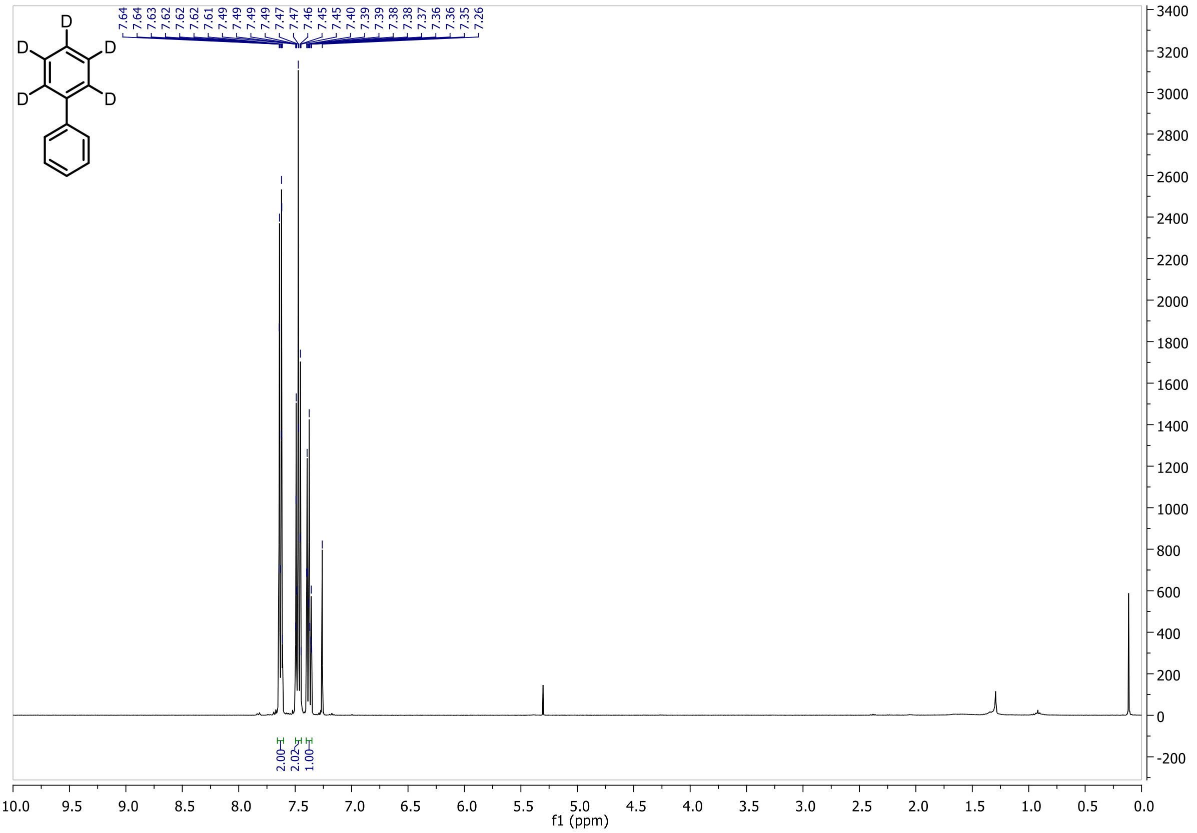

^{2}H NMR-{^{1}H}NMR (61 MHz, $CHCl_3$), 1,1'-biphenyl-2,3,4,5,6-d_5 (**[^{2}H$_5$]12**) :

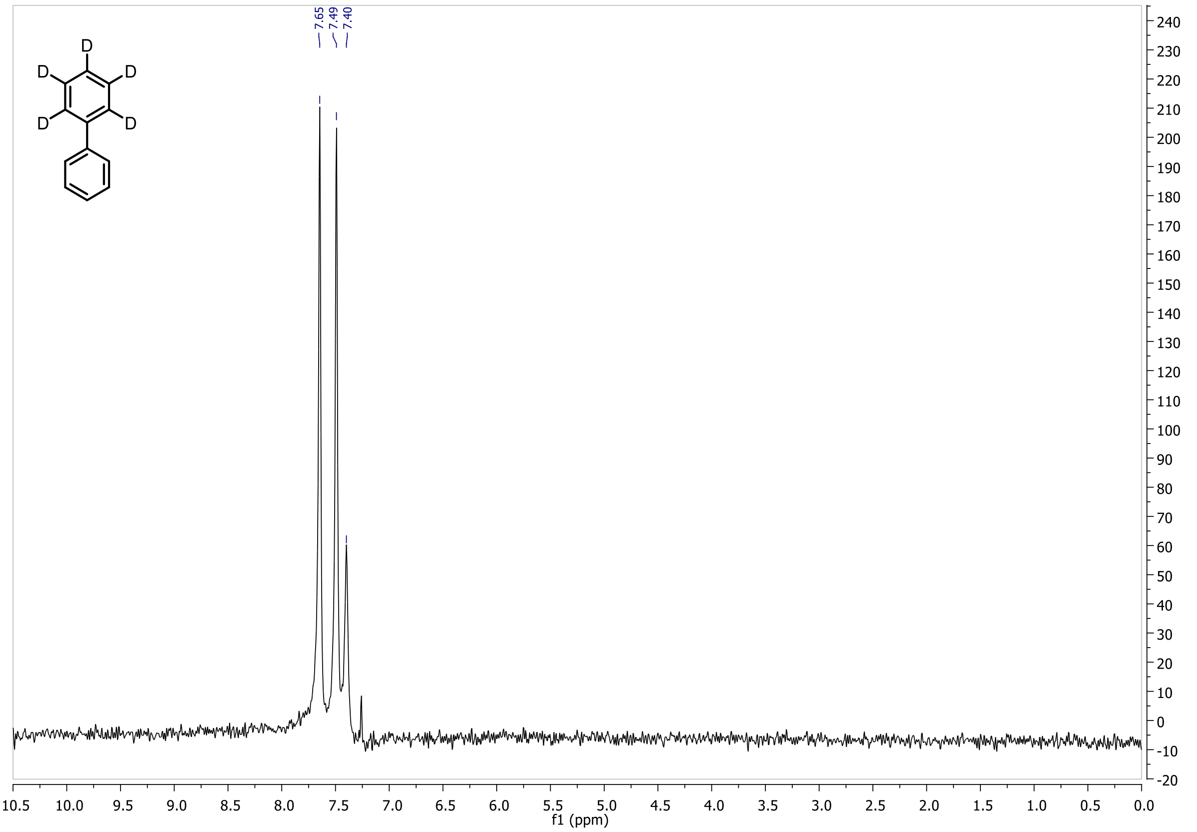

^{13}C NMR (100 MHz, $CDCl_3$), 1,1'-biphenyl-2,3,4,5,6-d_5 (**[$^2H_5$]12**) (1C-D missing)

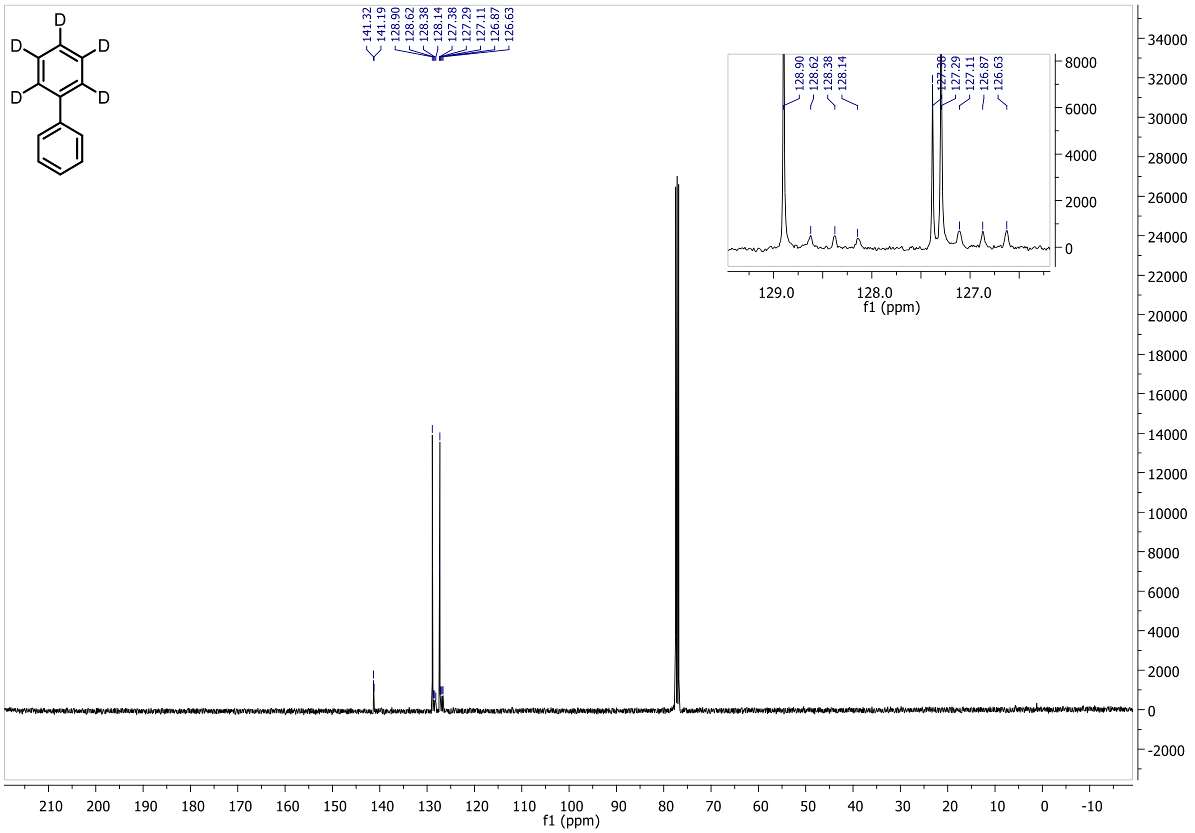

^{13}C NMR (100 MHz, $CDCl_3$), Top) biphenyl. Bottom) 1,1'-biphenyl-2,3,4,5,6-d_5 (**[$^2H_5$]12**)

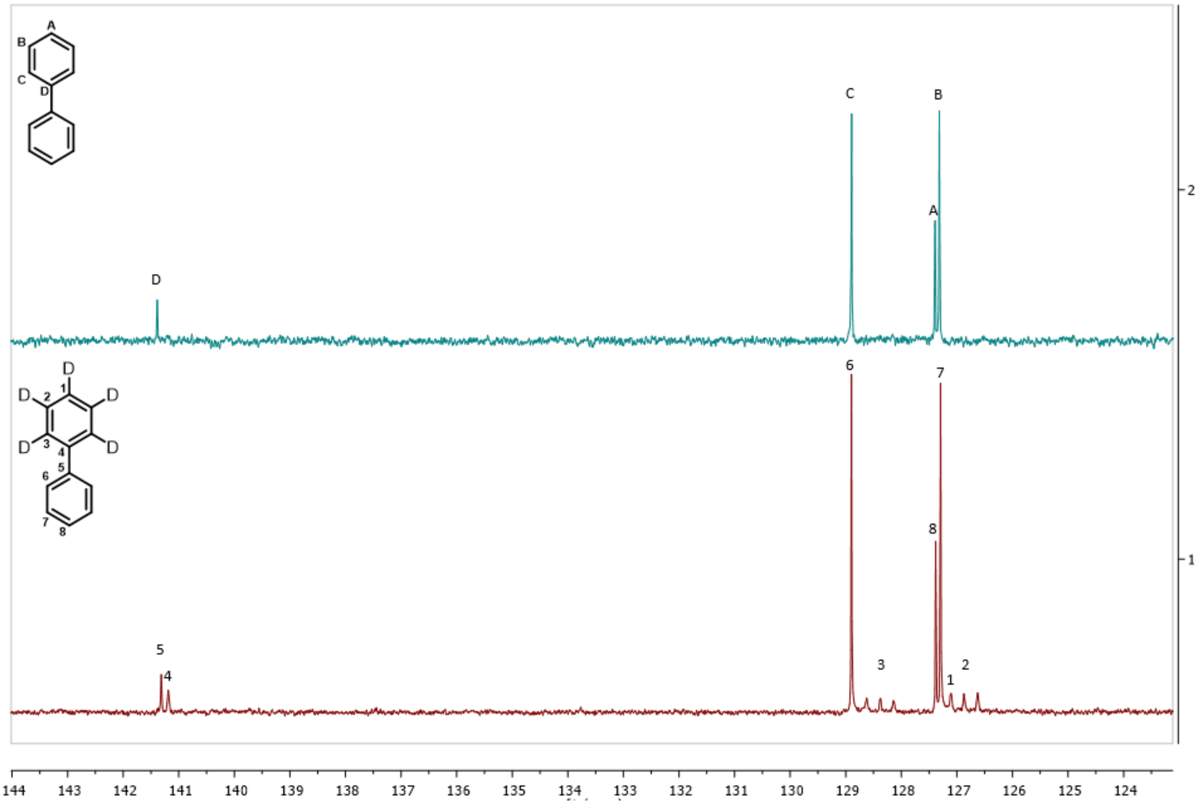

^{1}H NMR (400 MHz, $CDCl_3$), 1-(phenyl-$^{13}C_6$)dibenzo[b,d]furan (**[^{13}C$_6$]13**)

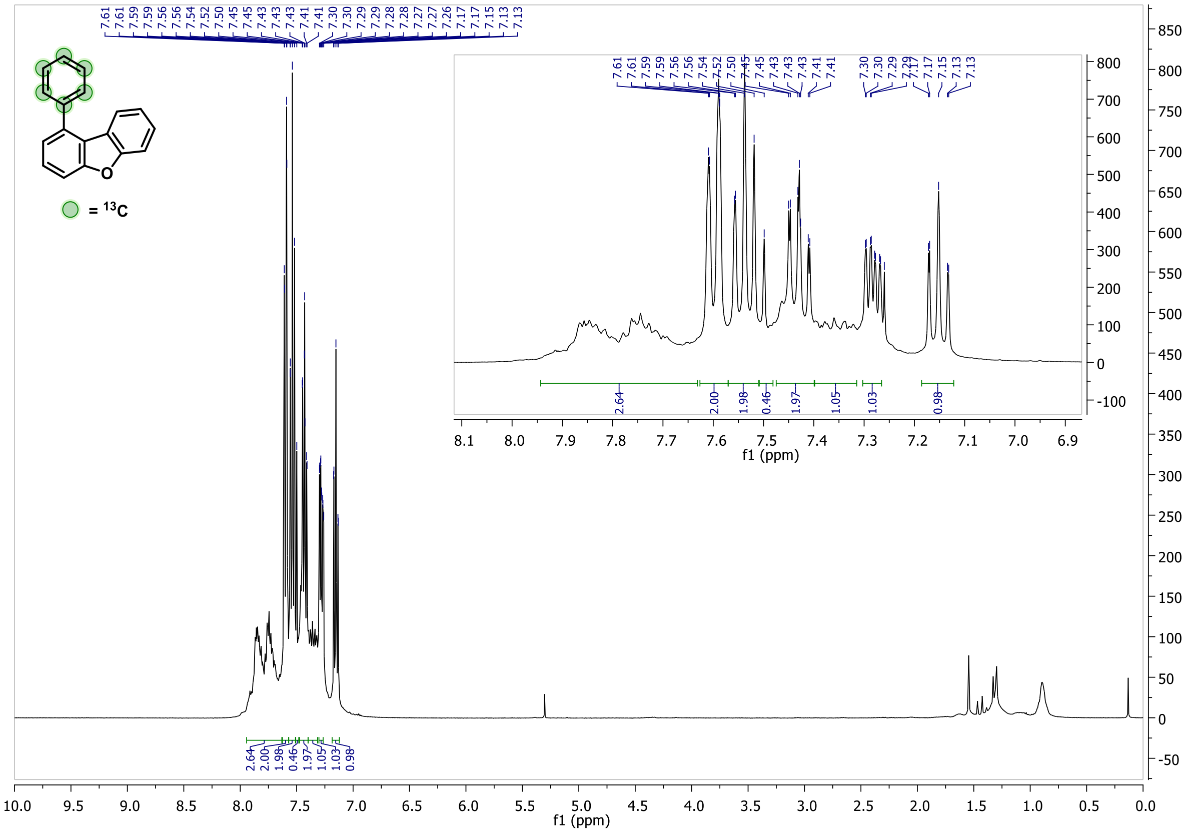

^{1}H NMR-{^{13}C}NMR (400 MHz, $CHCl_3$), 1-(phenyl-$^{13}C_6$)dibenzo[b,d]furan (**[^{13}C$_6$]13**)

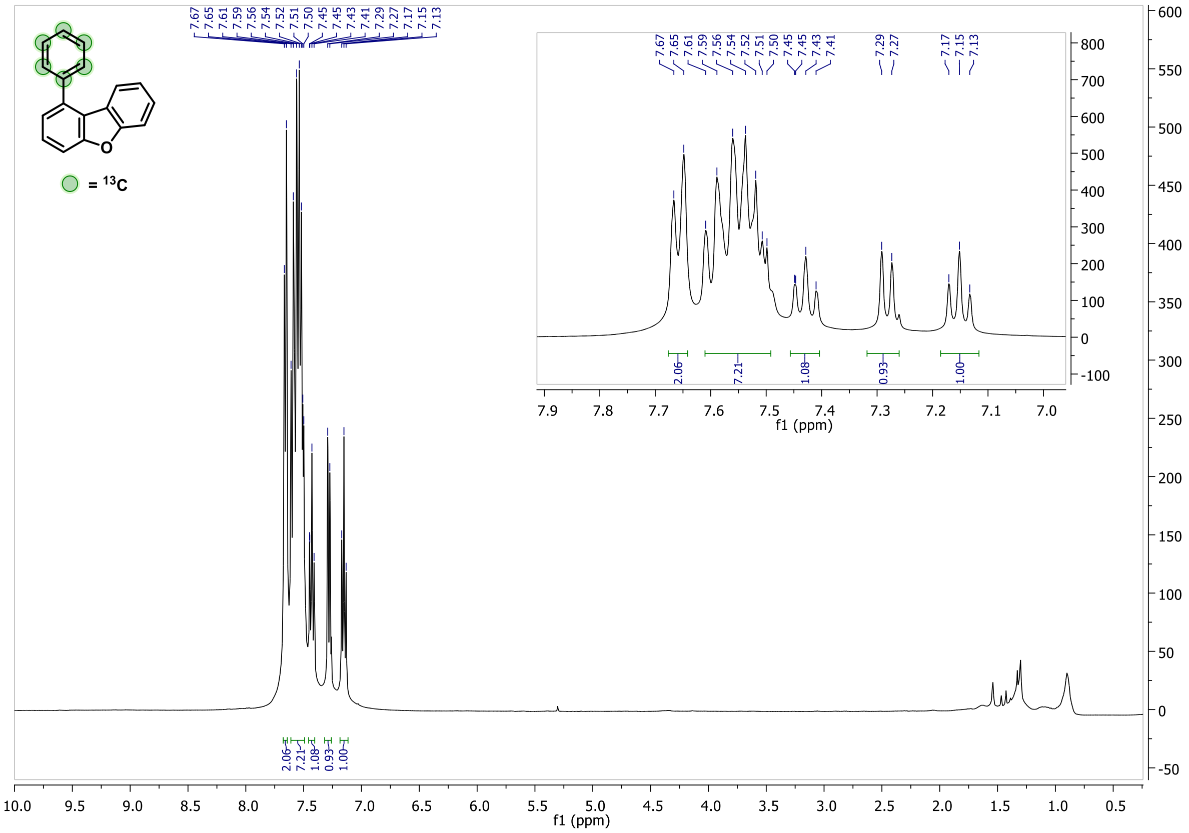

^{13}C NMR (100 MHz, $CDCl_3$), 1-(phenyl-$^{13}C_6$)dibenzo[b,d]furan (**[$^{13}C_6$]13**)

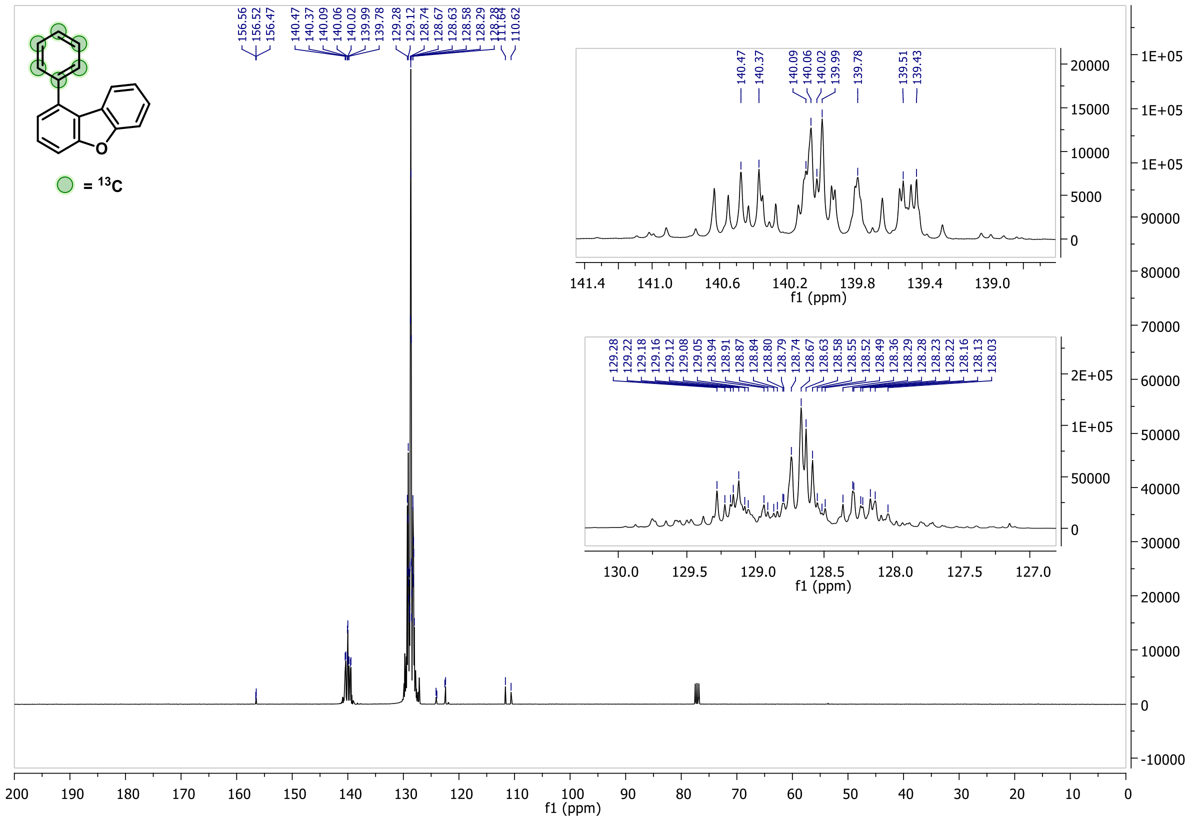

^{1}H NMR (400 MHz, $CDCl_3$), 4-(phenyl-$^{13}C_6$)dibenzo[b,d]furan (**[$^{13}C_6$]14**)

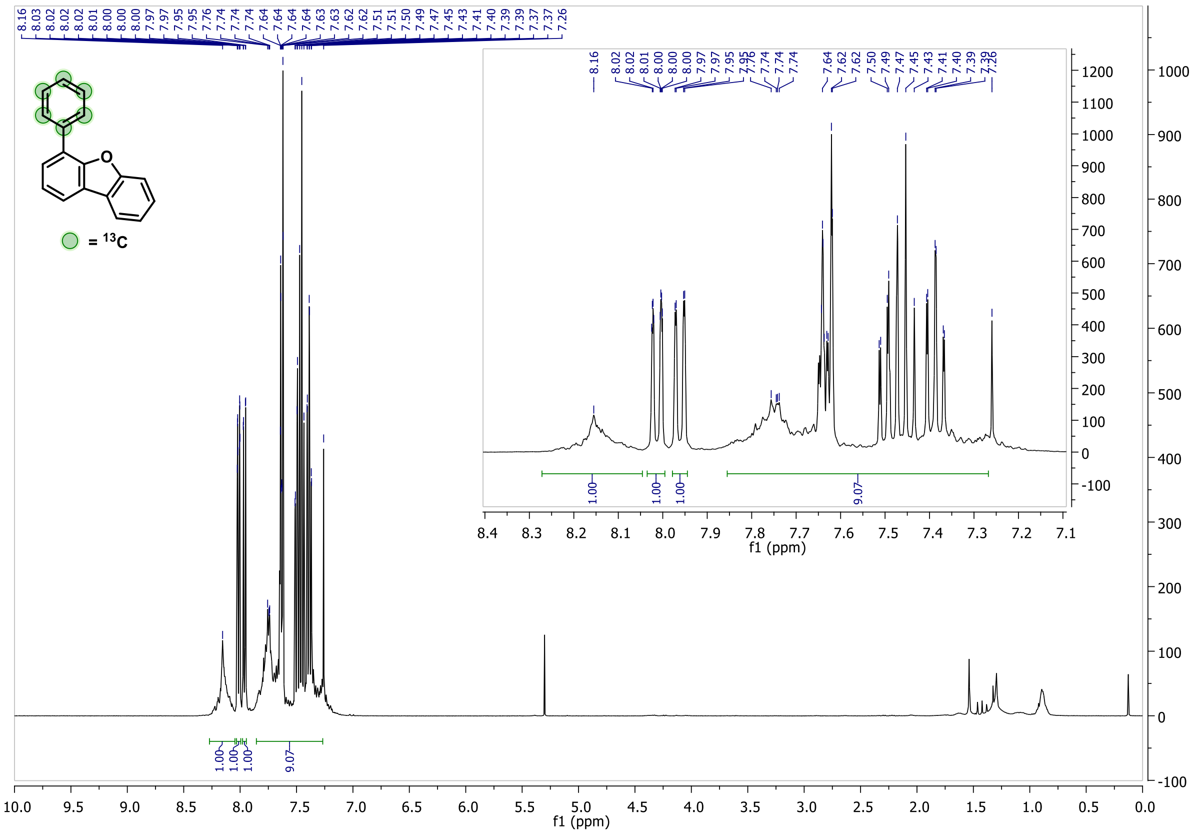

^{1}H NMR-{^{13}C}NMR (400 MHz, $CDCl_3$), 4-(phenyl-$^{13}C_6$)dibenzo[b,d]furan (**[$^{13}C_6$]14**)

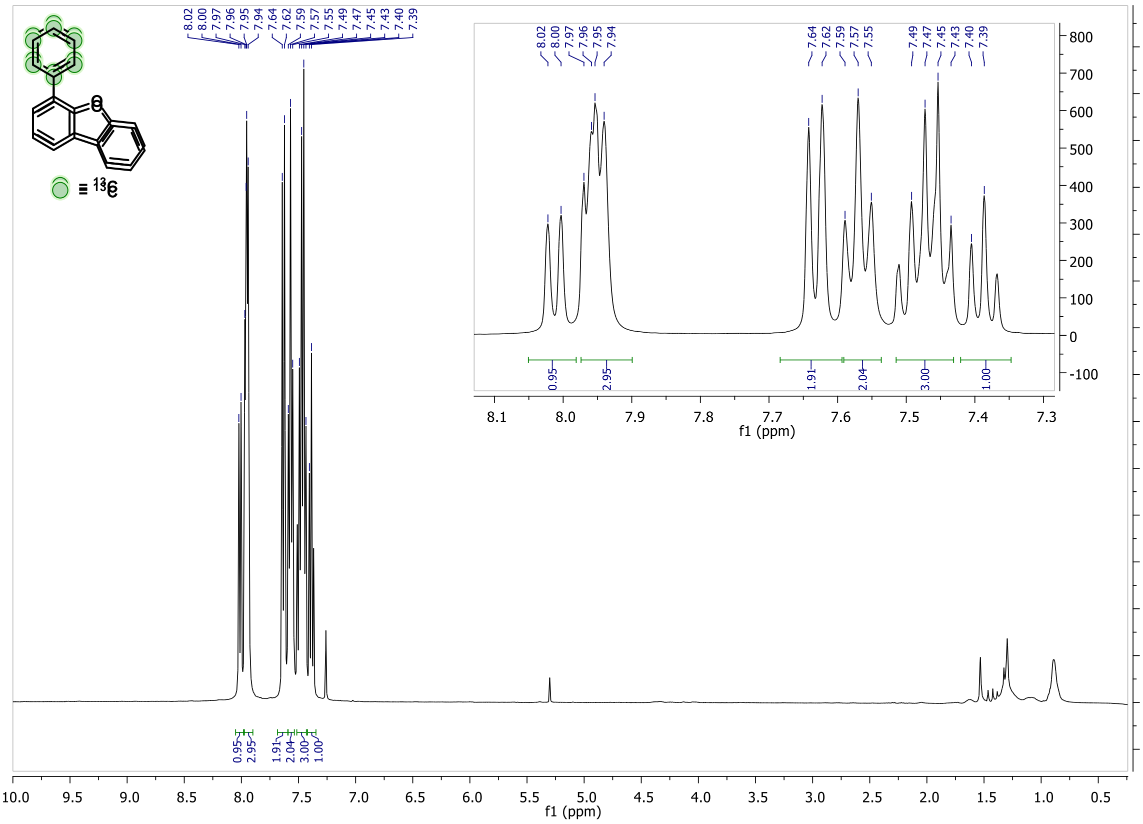

^{13}C NMR (100 MHz, $CDCl_3$), 4-(phenyl-$^{13}C_6$)dibenzo[b,d]furan (**[$^{13}C_6$]14**)

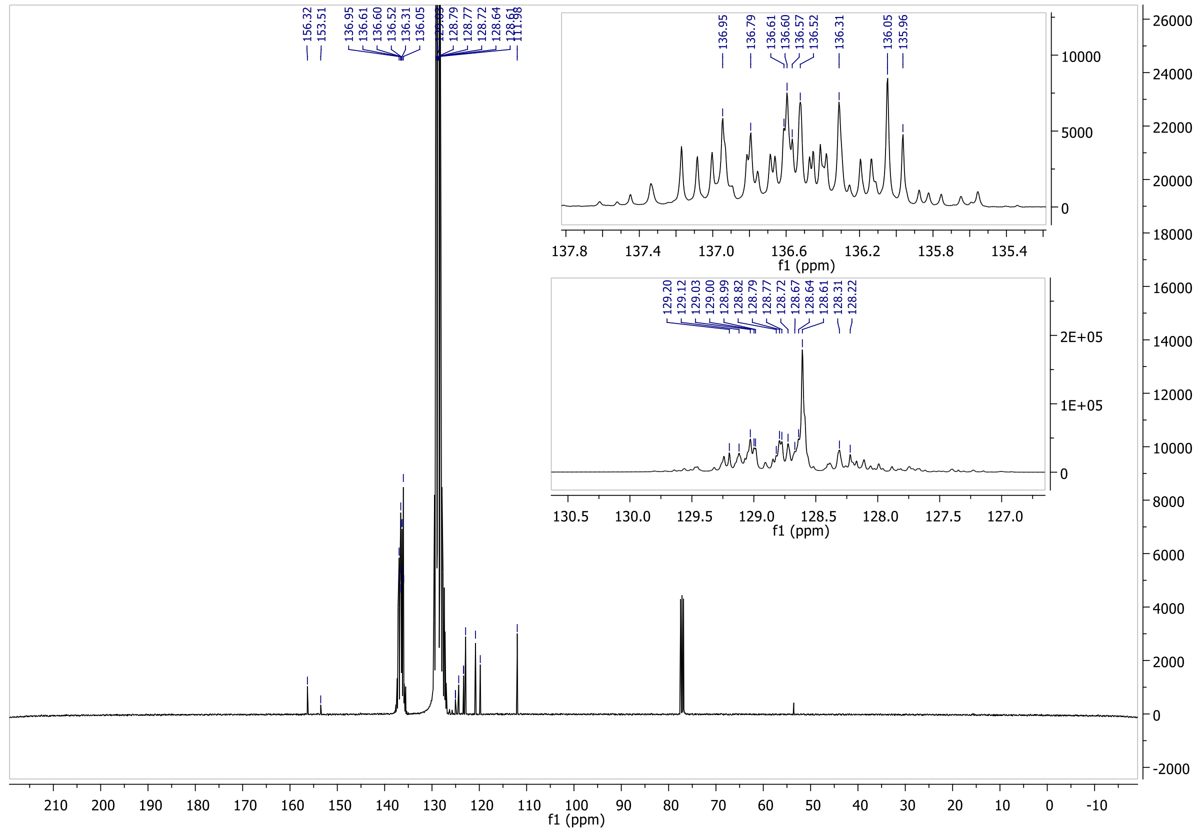

^{1}H NMR-{^{13}C}NMR (400 MHz, $CDCl_3$), Isopropyl 2-methyl-2-(4-(4-(phenyl-^{13}C)benzoyl)phenoxy)propanoate (**[^{13}C6]15**)

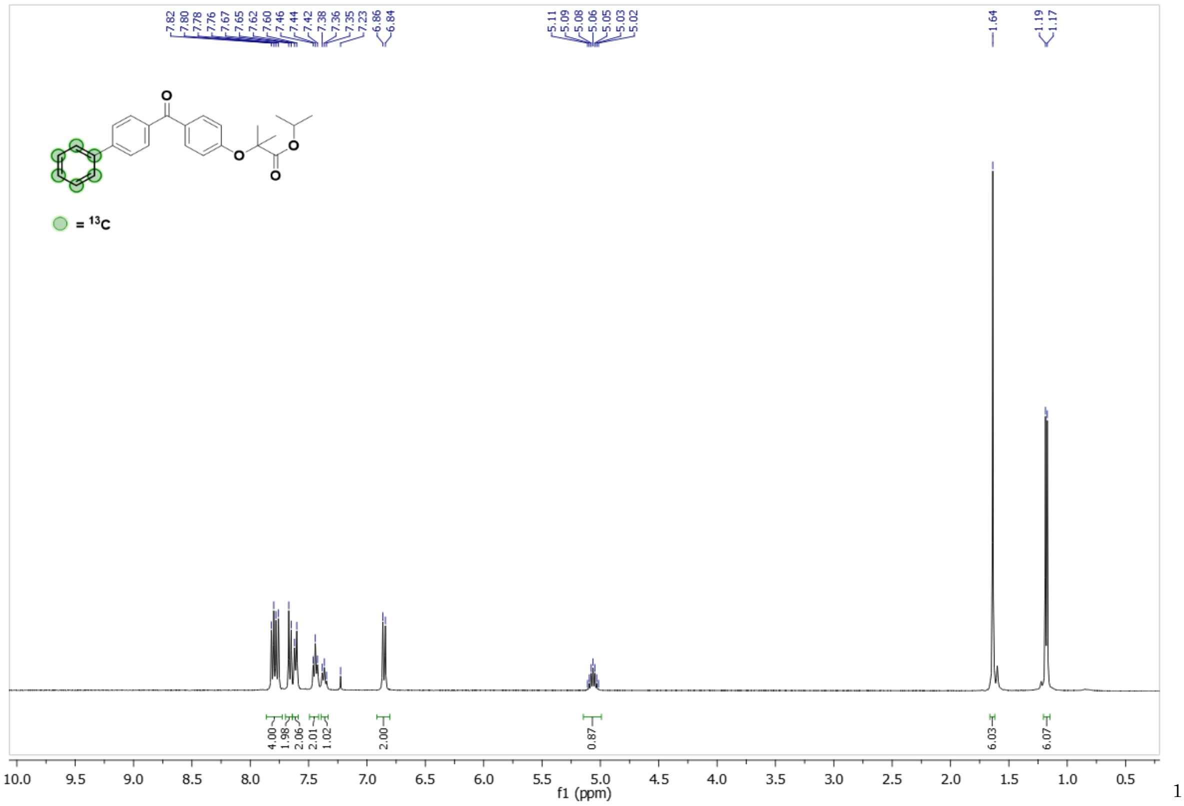

[3]C NMR (100 MHz, $CDCl_3$), Isopropyl 2-methyl-2-(4-(4-(phenyl-^{13}C6)benzoyl)phenoxy)propanoate (**[^{13}C6]15**)

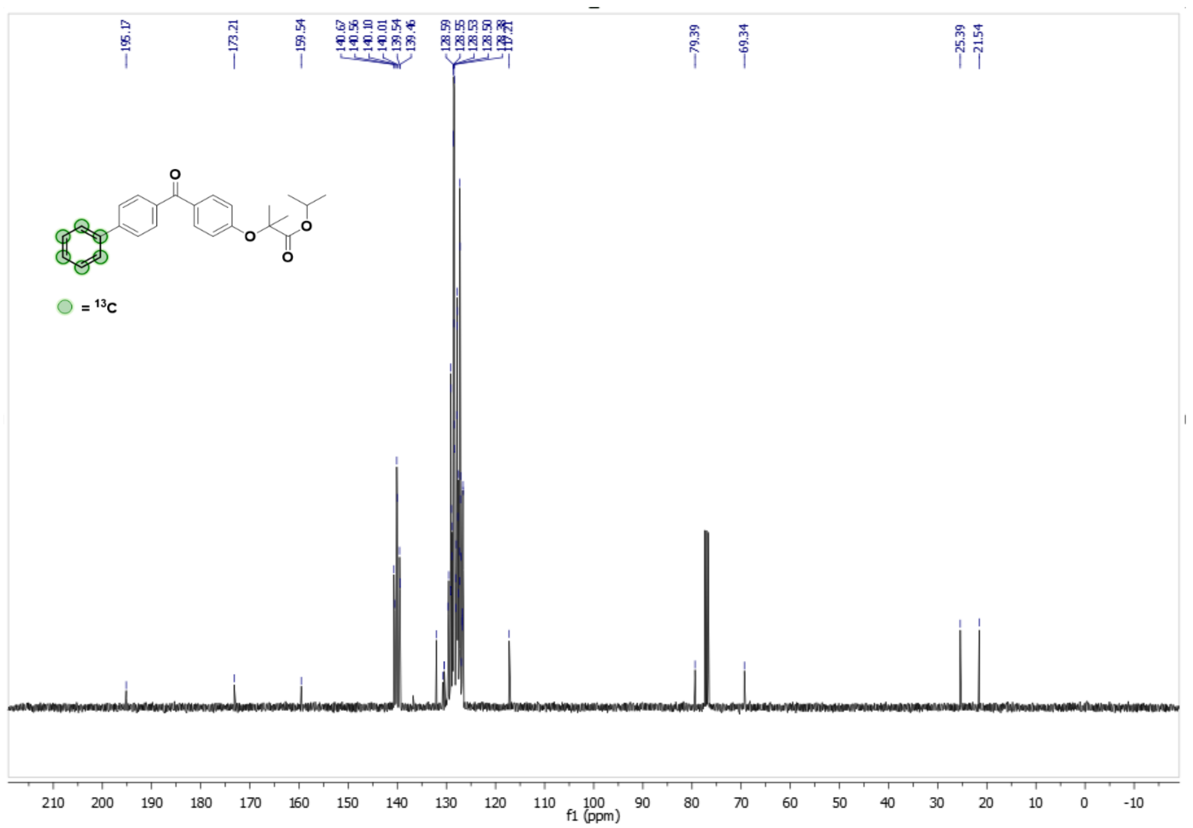

^{1}H NMR-{^{13}C}NMR (400 MHz, $CDCl_3$), Methyl 2-(4-(phenyl-$^{13}C_6$)phenyl)acetate (**[$^{13}C_6$]16**)

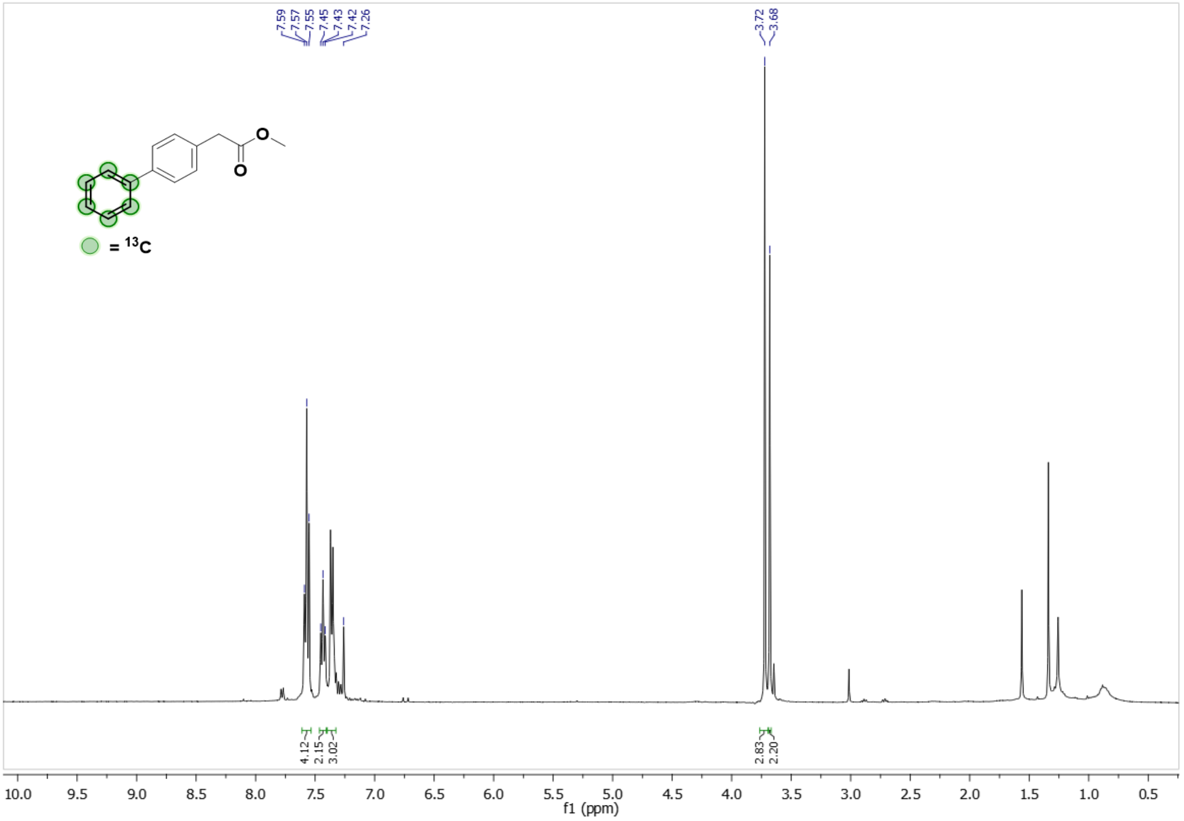

^{13}C NMR (100 MHz, $CDCl_3$), Methyl 2-(4-(phenyl-$^{13}C_6$)phenyl)acetate (**[$^{13}C_6$]16**)

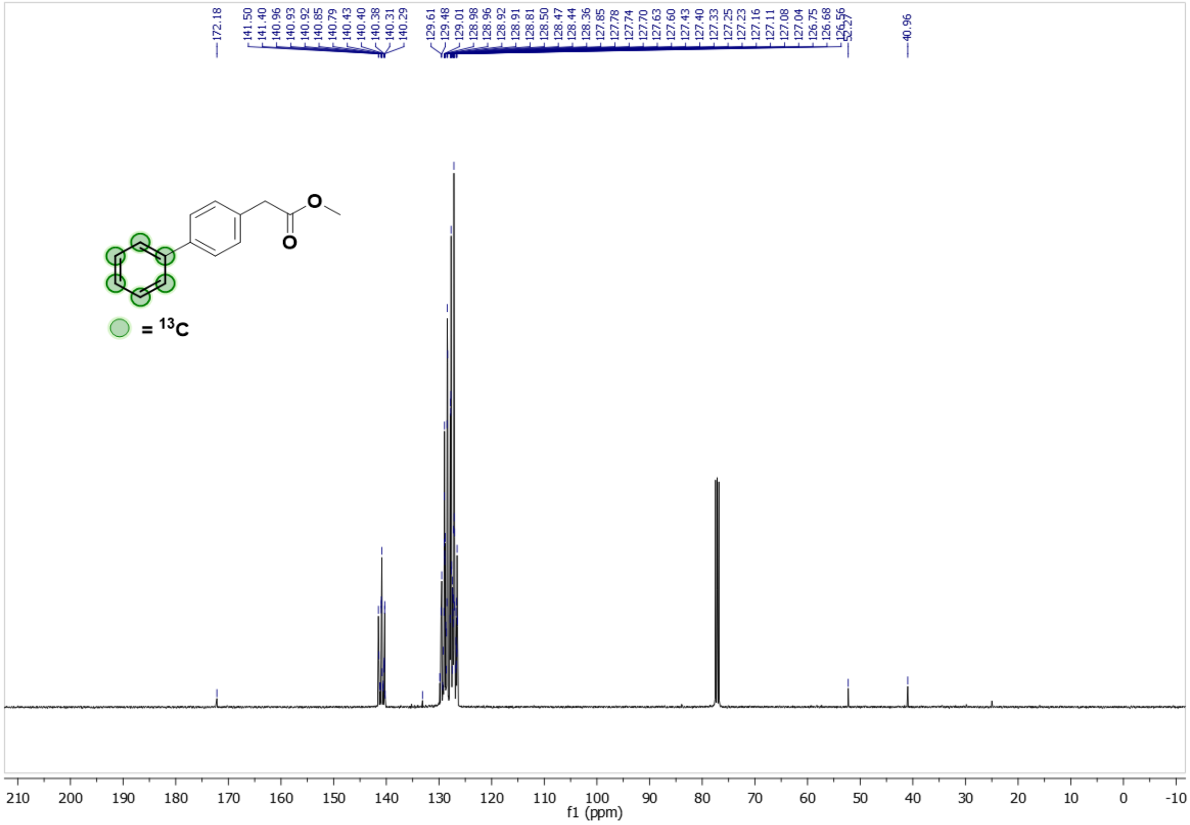

^{1}H NMR (400 MHz, Methanol-d_4), Ethyl 4-(8-(phenyl-13C6)-5,6-dihydro-11H benzo[5,6]cyclohepta[1,2-b]pyridin-11 ylidene)piperidine-1-carboxylate (**[^{13}C$_6$]17**)

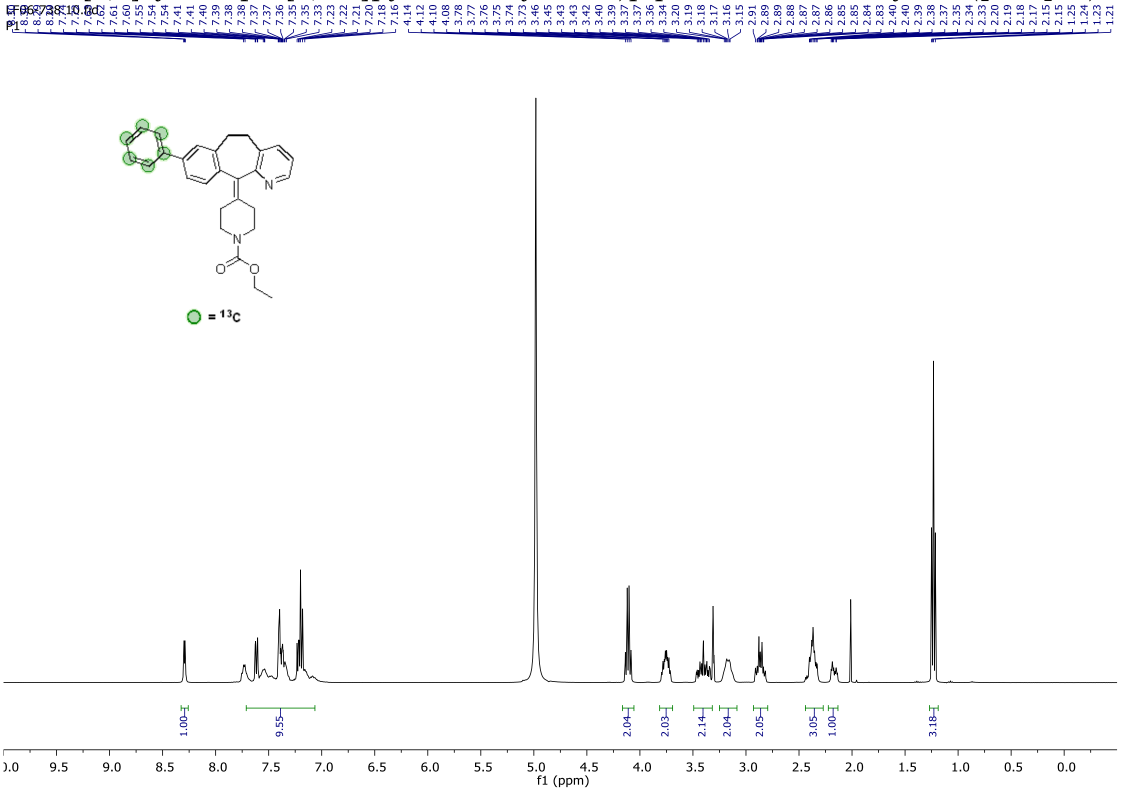

^{1}H NMR-{^{13}C}NMR (400 MHz, Methanol-d_4), Ethyl 4-(8-(phenyl-13C6)-5,6-dihydro 11H benzo[5,6]cyclohepta[1,2-b]pyridin-11 ylidene)piperidine-1-carboxylate (**[^{13}C$_6$]17**)

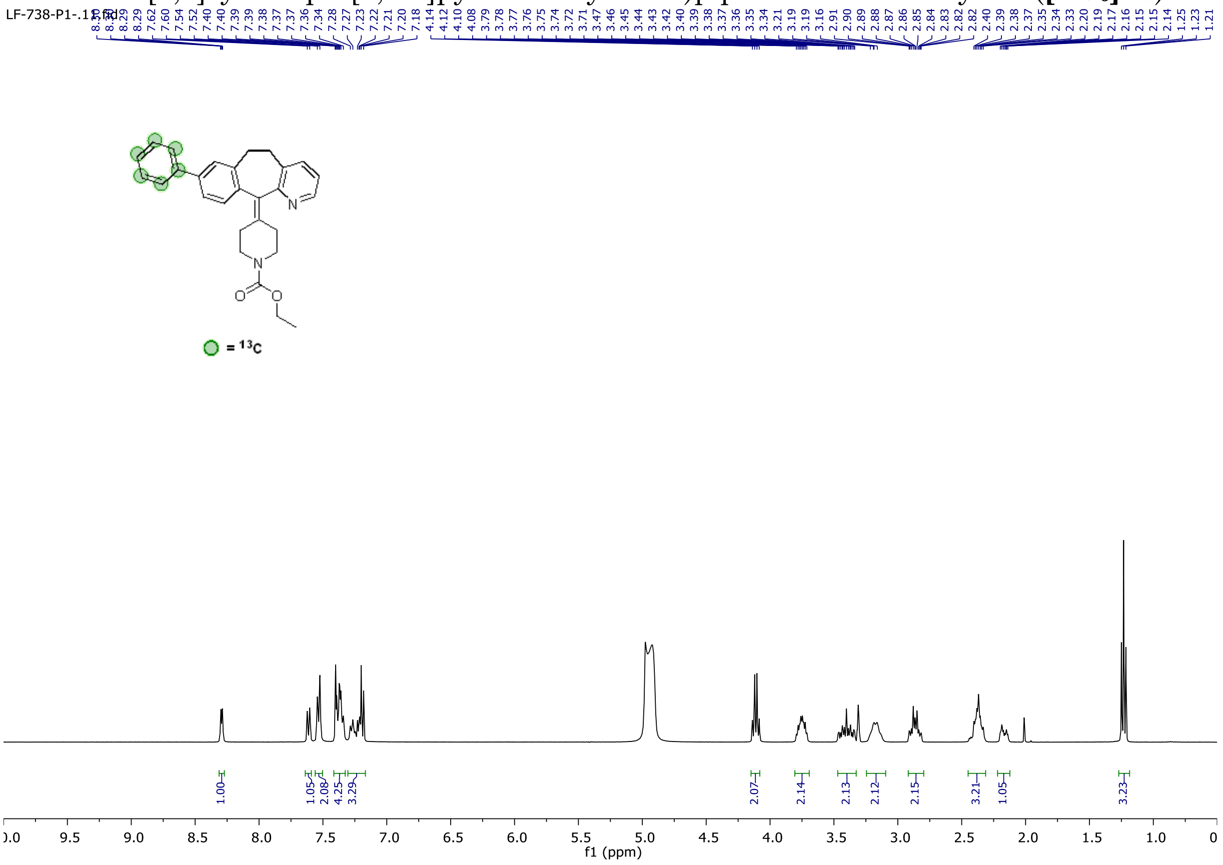

^{13}C NMR (101 MHz, Methanol-d_4) Ethyl 4-(8-(phenyl-13C6)-5,6-dihydro-11H benzo[5,6]cyclohepta[1,2-b]pyridin-11 ylidene)piperidine-1-carboxylate (**[$^{13}C_6$]17**)

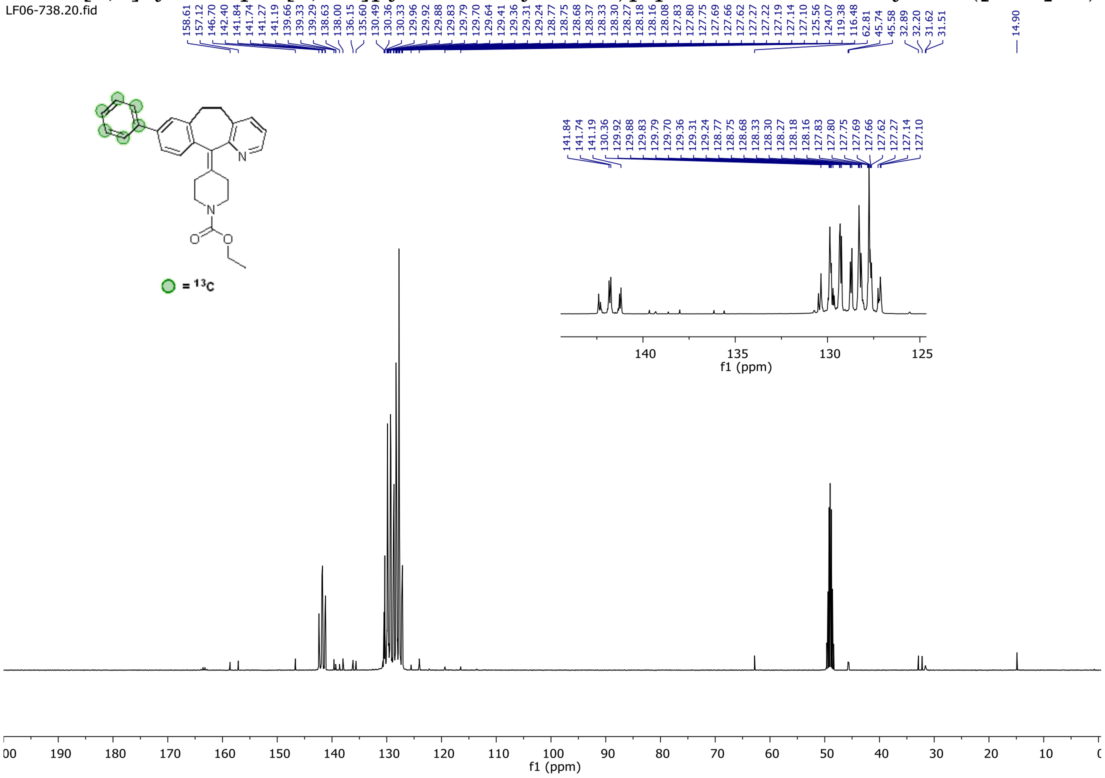

^{1}H NMR (400 MHz, Chloroform-d) Methyl 2-(5-methoxy-2-methyl-1-(4(phenyl-13C6) benzoyl)-1H-indol-3-yl)acetate **([^{13}C6]18)**

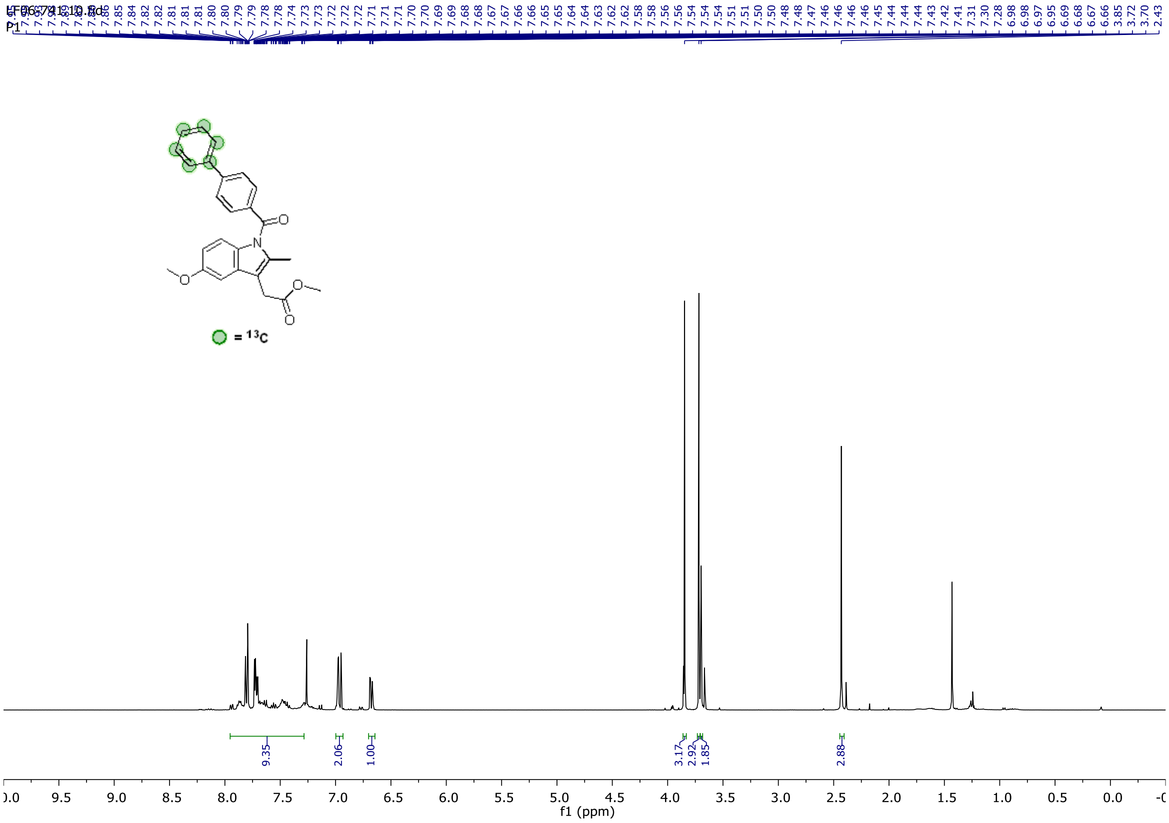

^{1}H NMR-{^{13}C}NMR (400 MHz, Chloroform-d), Methyl 2-(5-methoxy-2-methyl-1-(4(phenyl-13C6)benzoyl)-1H-indol-3-yl)acetate **([^{13}C6]18)**

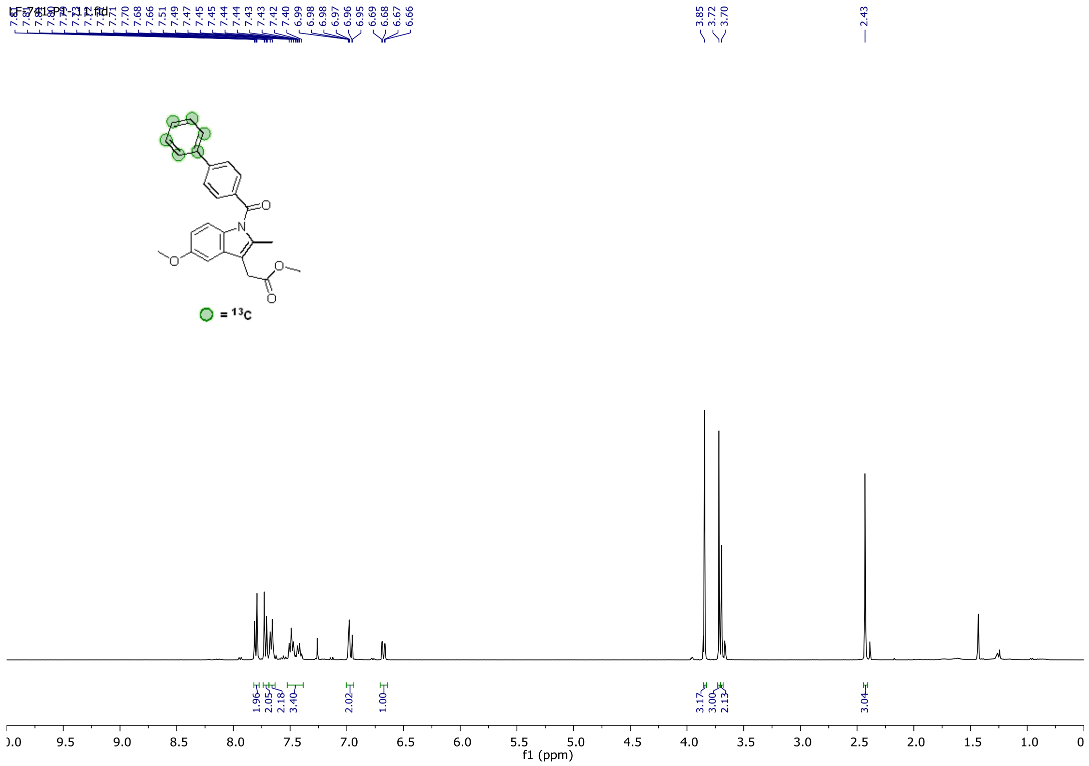

^{13}C NMR (101 MHz, $CDCl_3$) Methyl 2-(5-methoxy-2-methyl-1-(4(phenyl-13C6)benzoyl)-1H-indol-3-yl)acetate **([^{13}C6]18)**

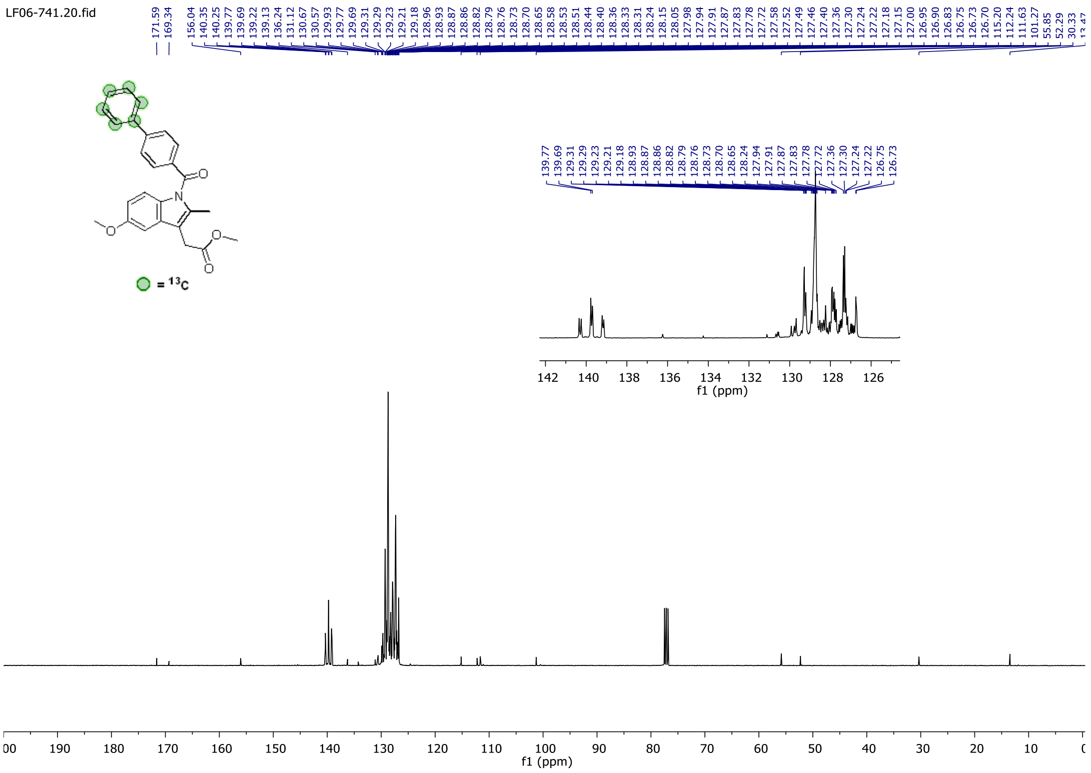

^{1}H NMR (400 MHz, $CDCl_3$), Ethyl 2-((phenyl-D_5)thio)acetate (**[$^2H_5$]19**)

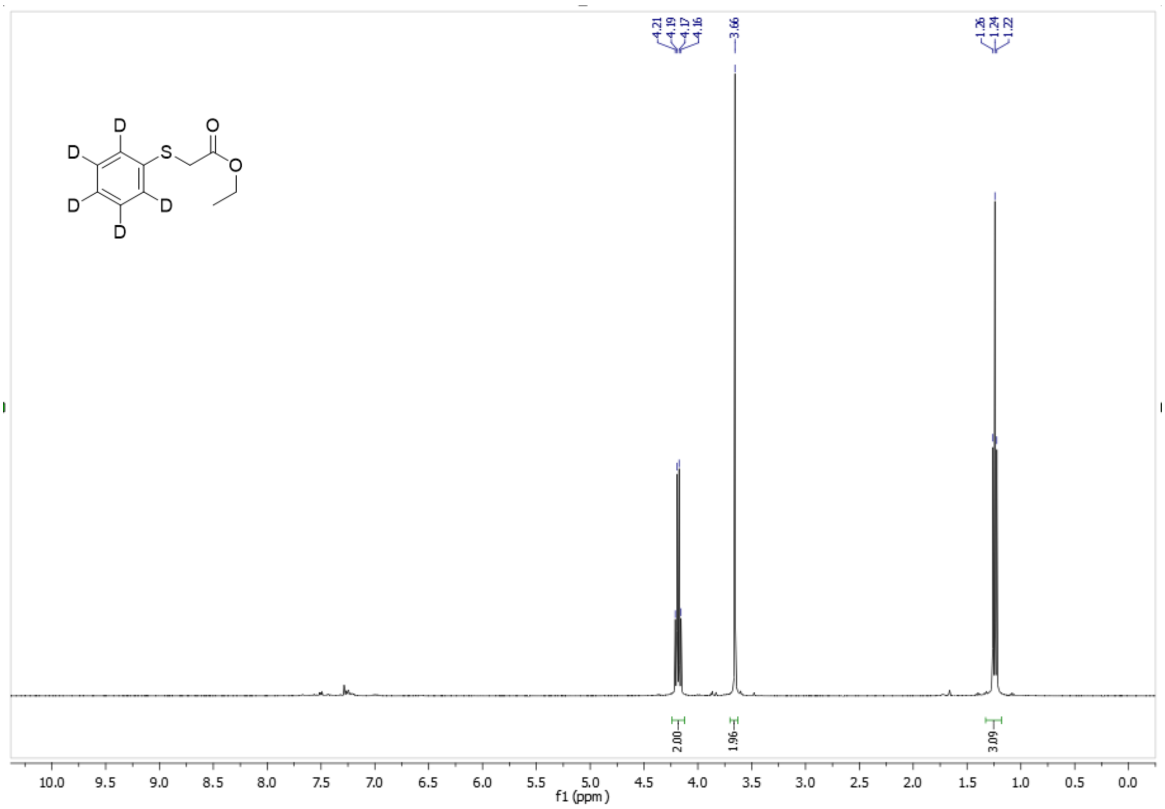

^{13}C NMR (100 MHz, $CDCl_3$), Ethyl 2-((phenyl-D5)thio)acetate (**[$^2H_5$]19**)

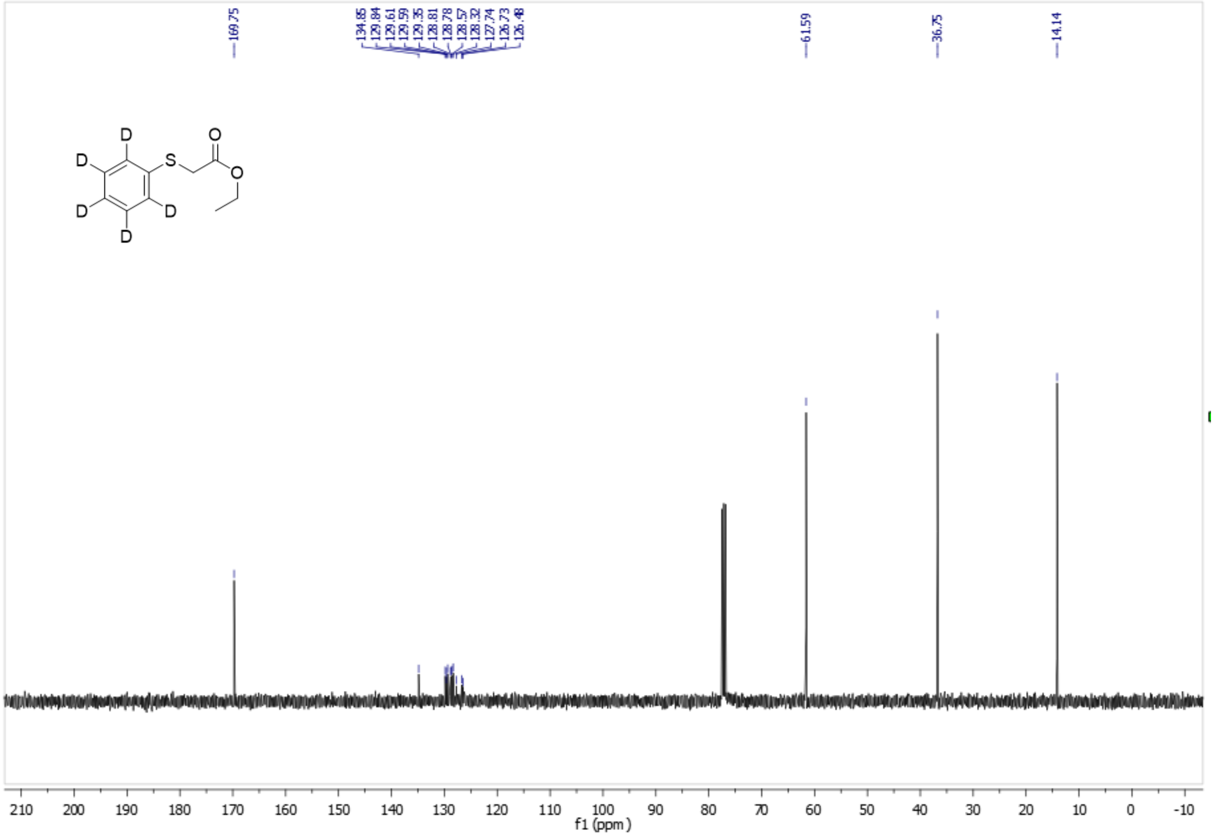

^{2}H NMR-{^{1}H}NMR (61 MHz, $CHCl_3$), Ethyl 2-((phenyl-D5)thio)acetate (**[$^{2}H_5$]19**)

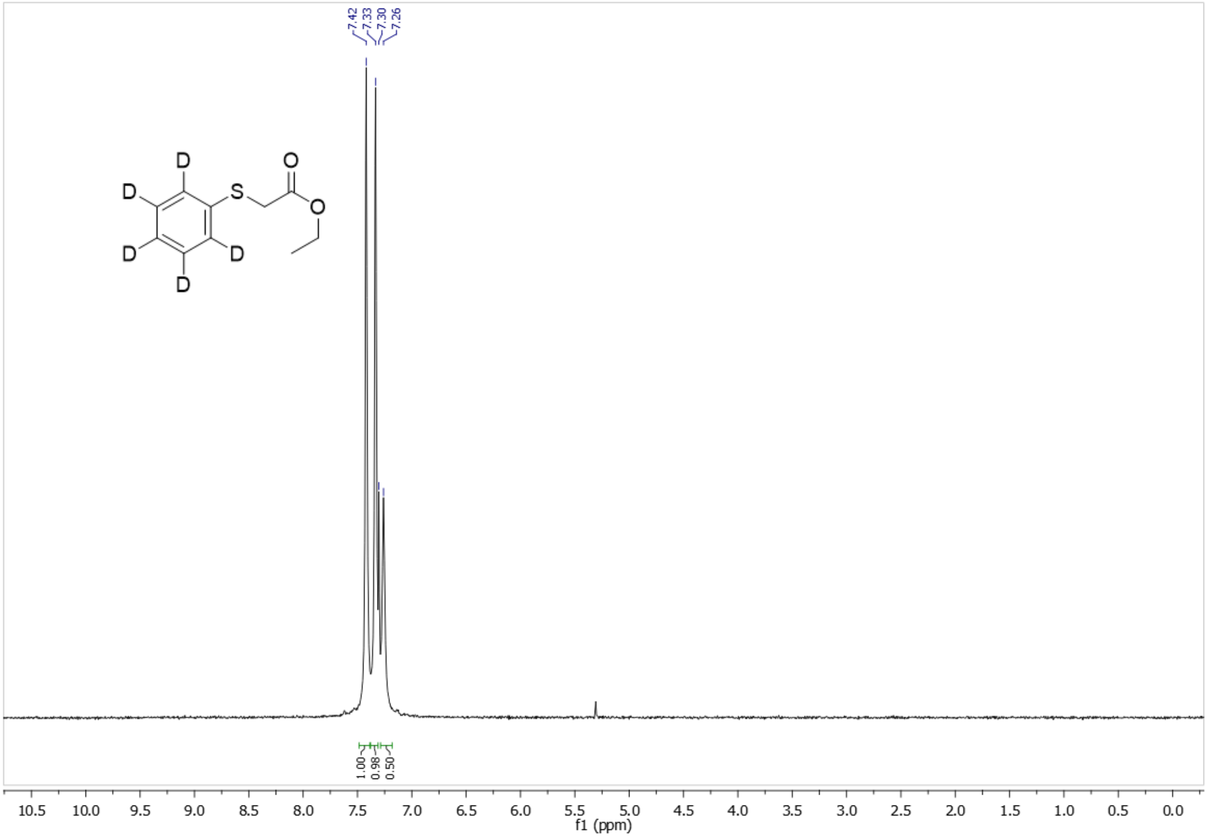

^{1}H NMR-{^{13}C}NMR (400 MHz, $CDCl_3$), methyl 2-((phenyl-$^{13}C_6$)thio)benzoate (**[$^{13}C_6$]20**)

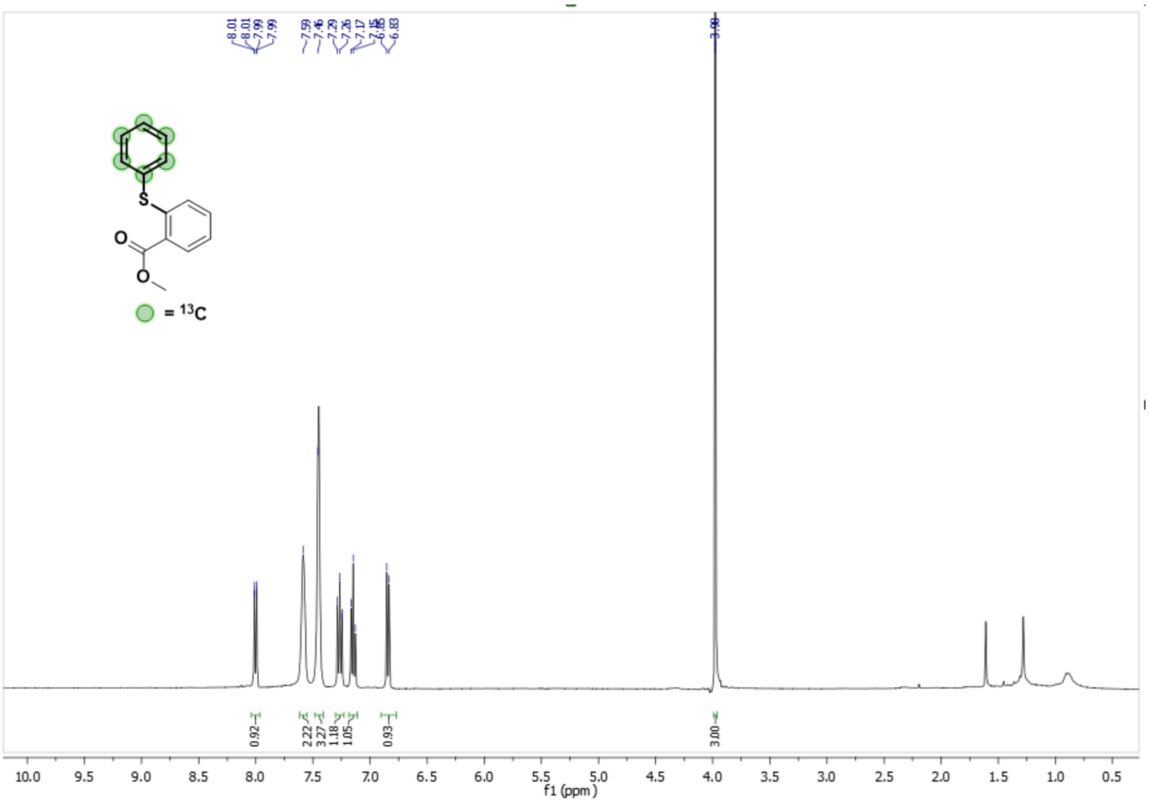

^{13}C NMR (100 MHz, $CDCl_3$), methyl 2-((phenyl-$^{13}C_6$)thio)benzoate (**[$^{13}C_6$]20**)

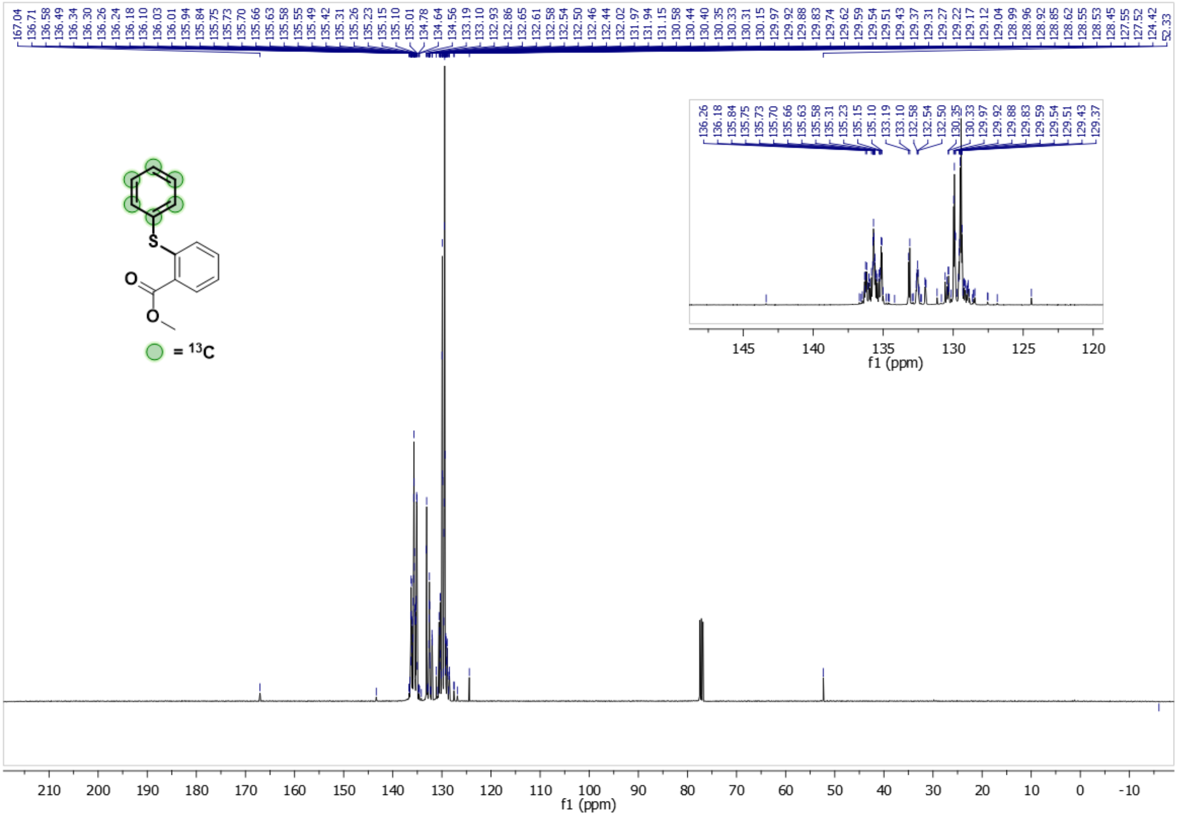

^{1}H NMR-{^{13}C}NMR (400 MHz, $CDCl_3$), 4-methyl-7-((phenyl-$^{13}C_6$)thio)-2H-chromen-2-one (**[$^{13}C_6$]21**)

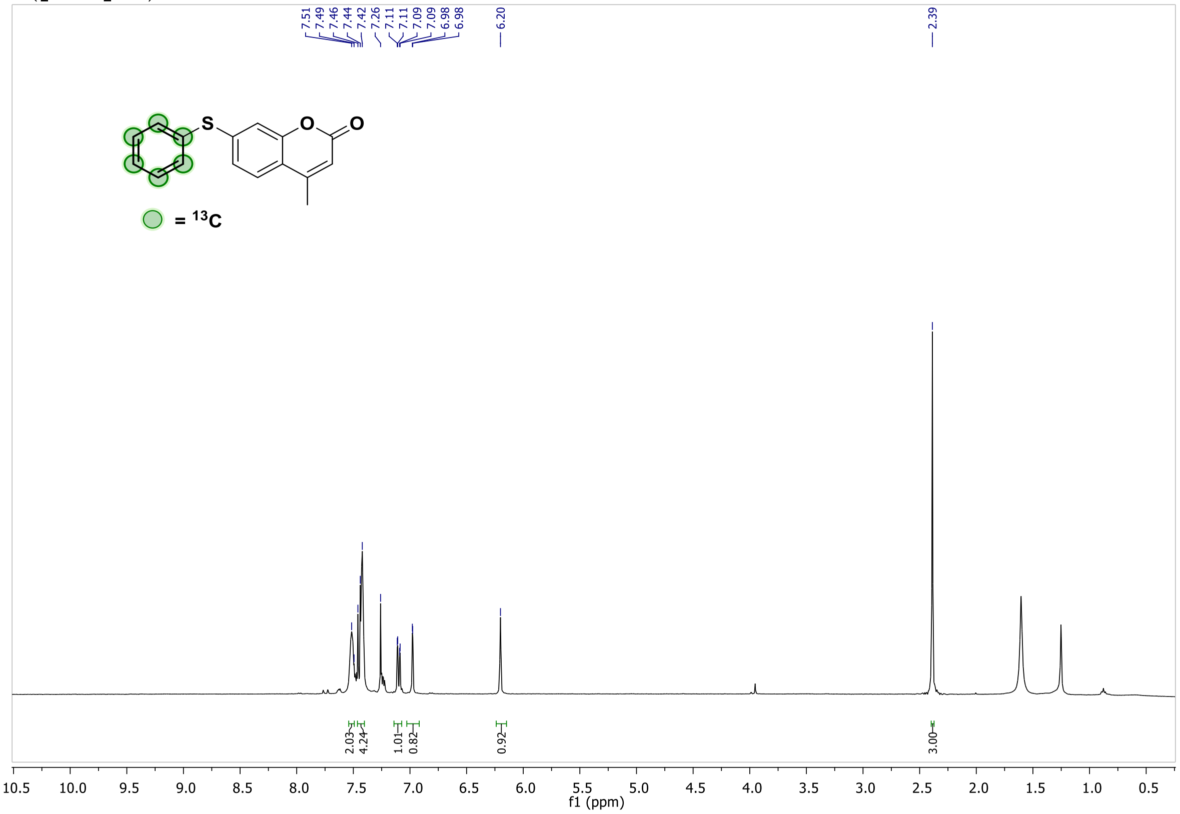

^{13}C NMR (100 MHz, $CDCl_3$), 4-methyl-7-((phenyl-$^{13}C_6$)thio)-2H-chromen-2-one (**[$^{13}C_6$]21**)

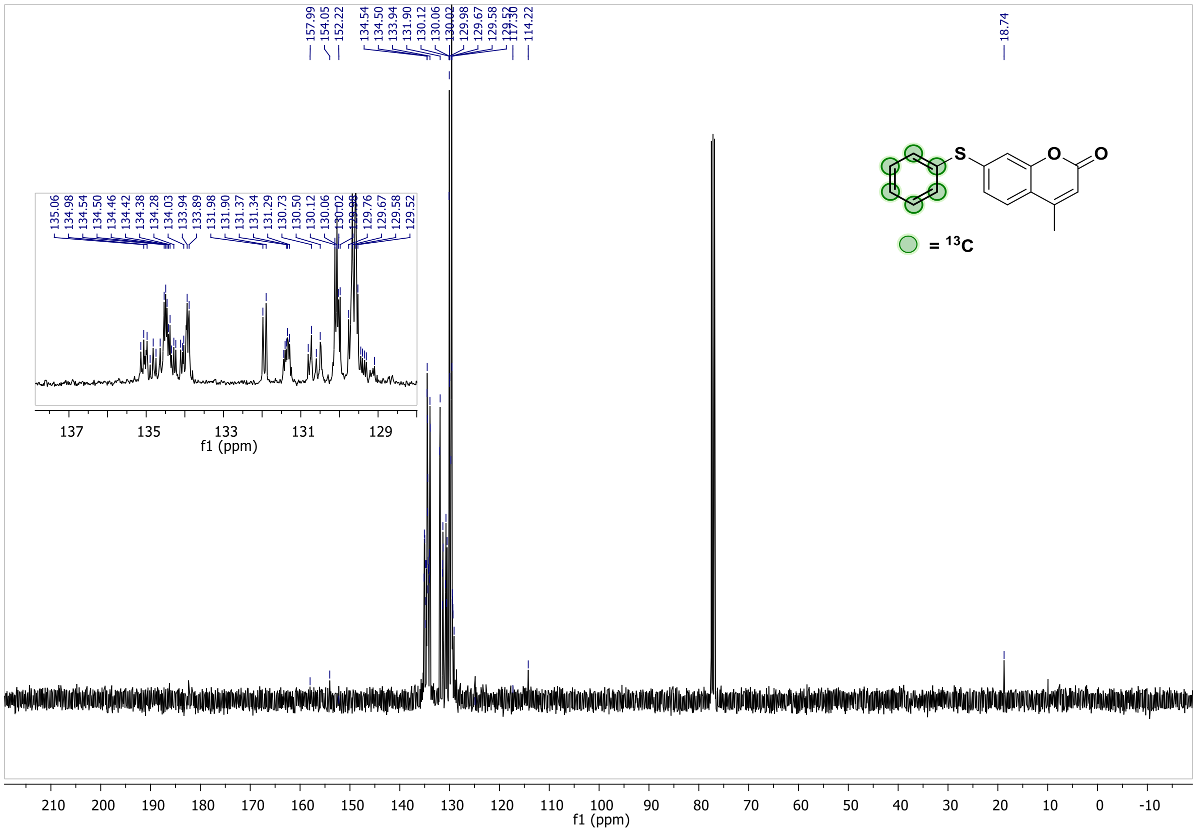

^1H NMR-{^{13}C} NMR (400 MHz, $CDCl_3$), Isopropyl 2-methyl-2-(4-(4-(phenyl-$^{13}C_6$)benzoyl)phenoxy) (**[$^{13}C_6$]22**)

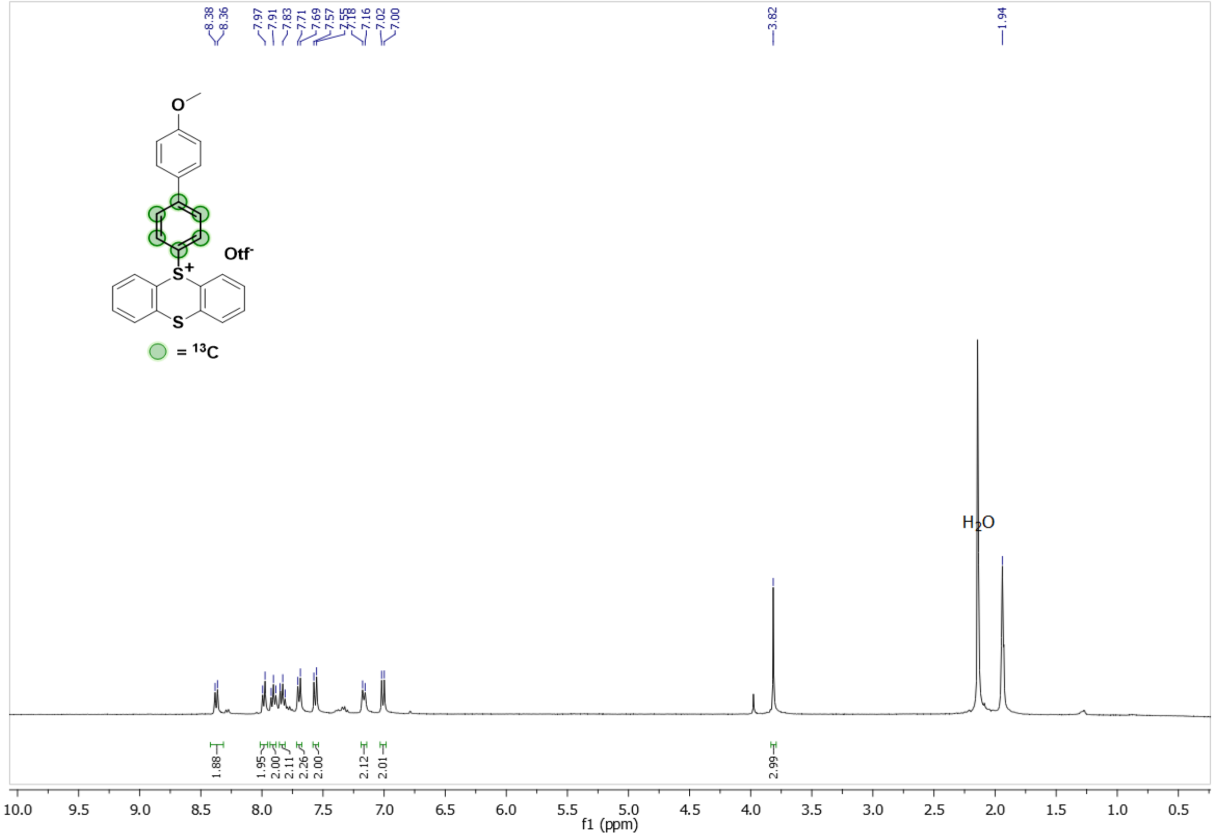

^{13}C NMR (100 MHz, $CDCl_3$), Isopropyl 2-methyl-2-(4-(4-(phenyl-$^{13}C_6$)benzoyl)phenoxy). (**[$^{13}C_6$]22**)

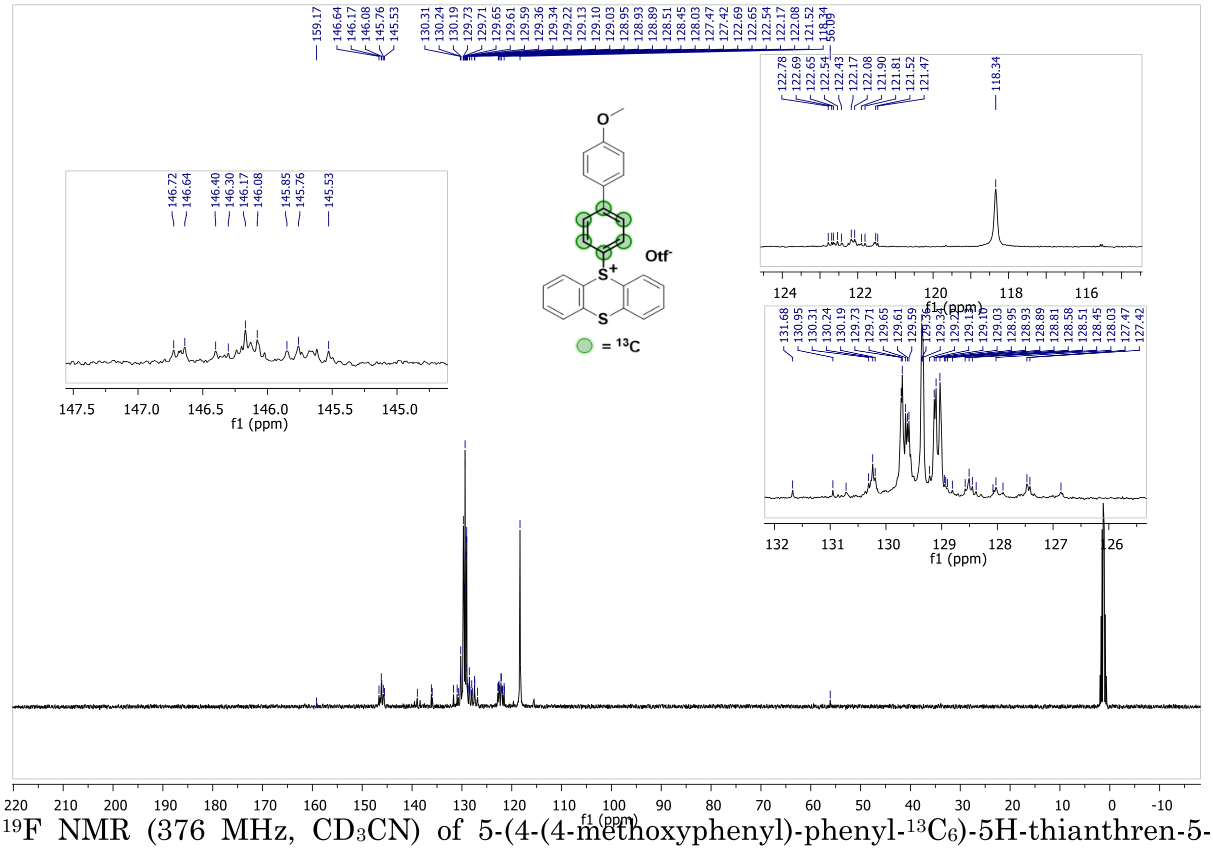

^{19}F NMR (376 MHz, CD_3CN) of 5-(4-(4-methoxyphenyl)-phenyl-$^{13}C_6$)-5H-thianthren-5-iumtrifluoromethanesulfonate (**[$^{13}C_6$]22**)

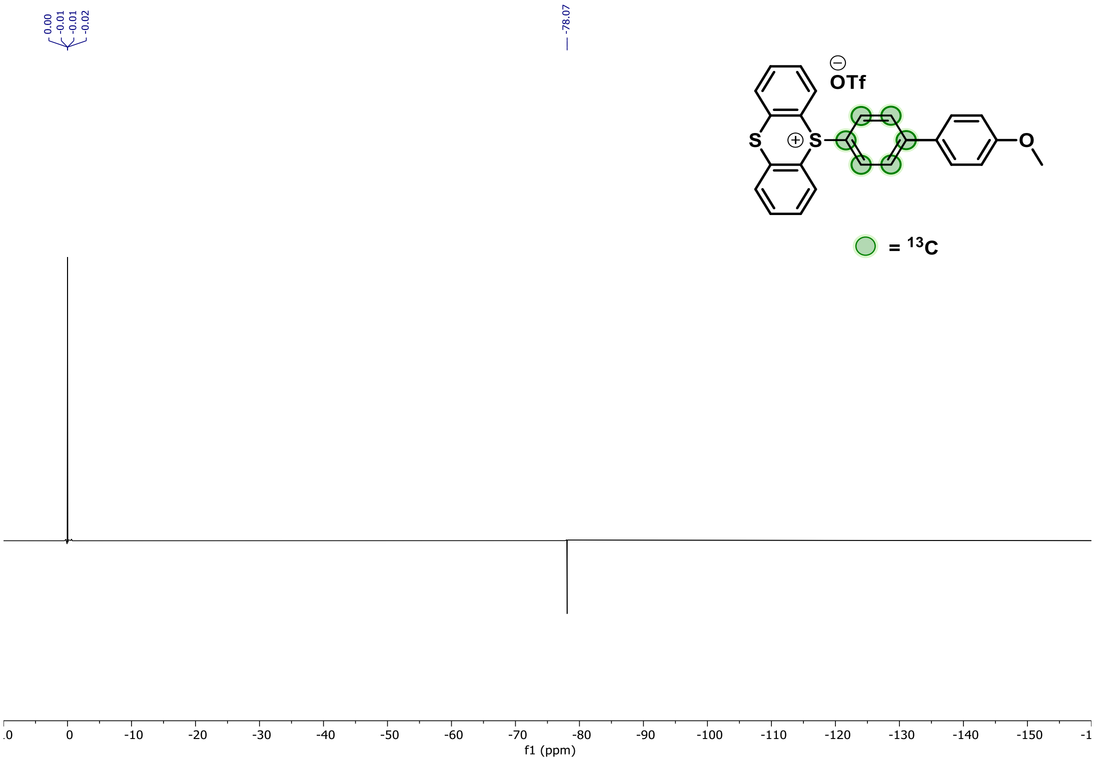

^{1}H NMR-{^{13}C} NMR (400 MHz, $CDCl_3$), Ethyl 2-((4-(4-methoxyphenyl)phenyl-1,2,3,4,5,6-$^{13}C_6$)thio)acetate(**[$^{13}C_6$]23**)

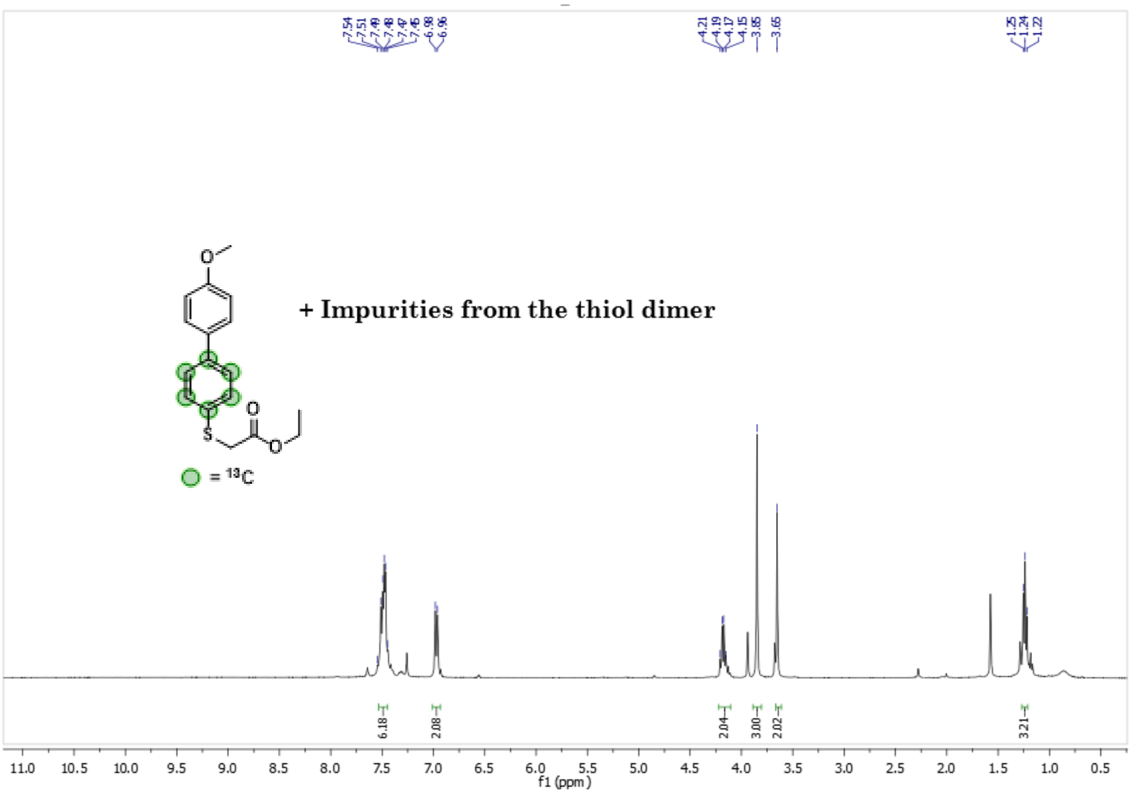

^{13}C NMR (100 MHz, $CDCl_3$), Ethyl 2-((4-(4-methoxyphenyl)phenyl-1,2,3,4,5,6-13C6)thio)acetate (**[$^{13}C_6$]23**)

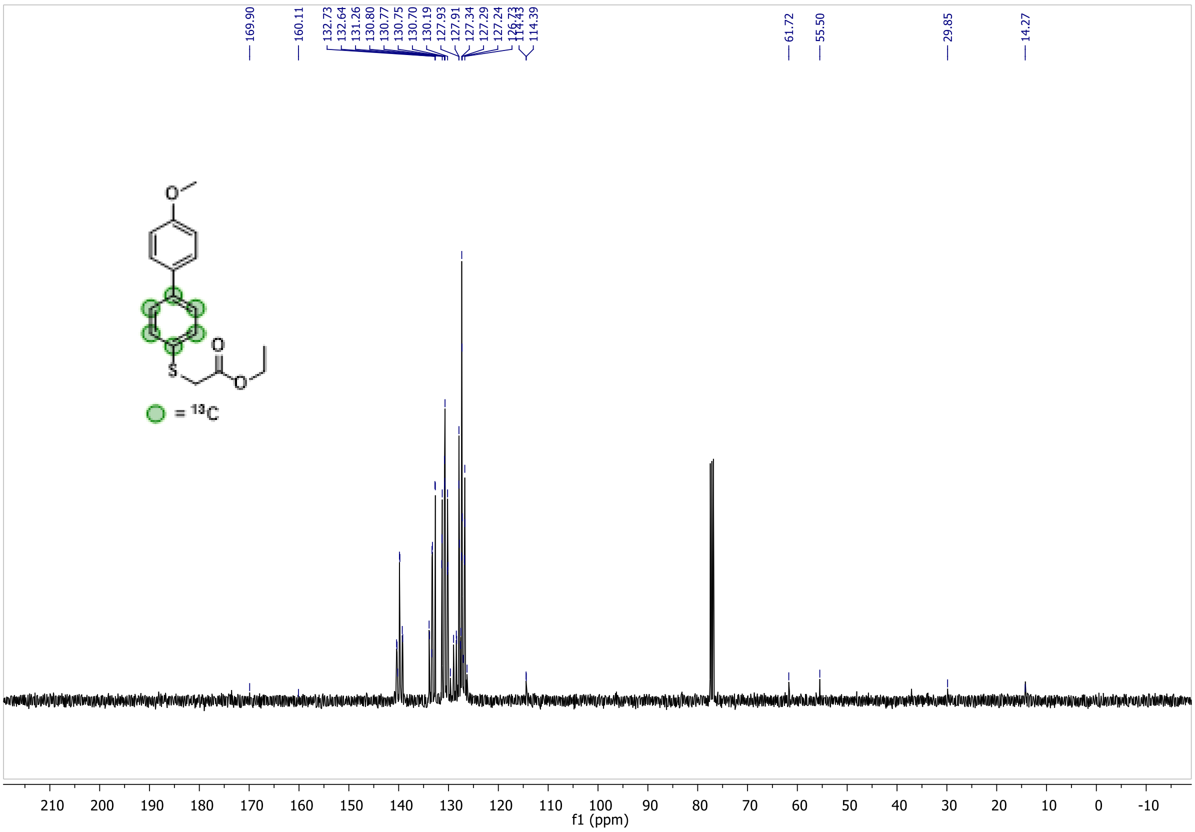

^{1}H NMR-{^{13}C} NMR (400 MHz, $CDCl_3$), 1-(4-methoxyphenyl)benzene-1,2,3,4,5,6-$^{13}C_6$-4-D (**[$^{13}C_6$, ^{2}H]24**)

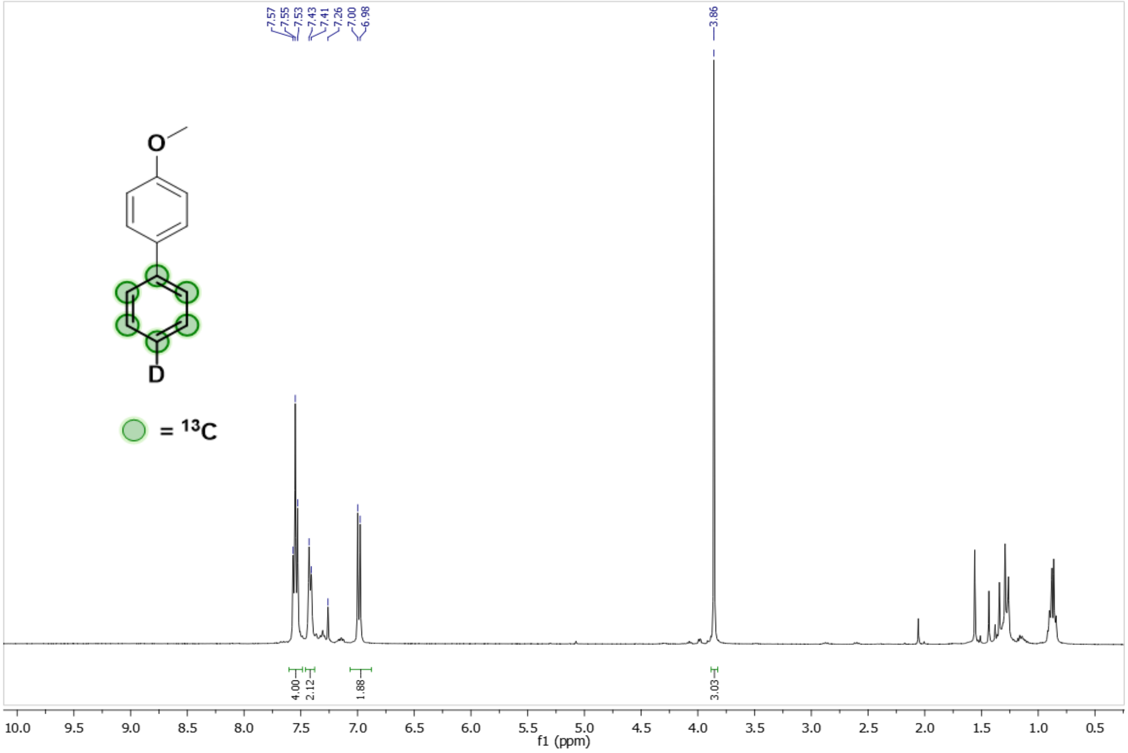

^{1}H

NMR-{^{13}C} NMR (400 MHz, $CDCl_3$), 1-(4-methoxyphenyl)benzene-1,2,3,4,5,6-$^{13}C_6$-4-D (**[$^{13}C_6$, ^{2}H]24**)

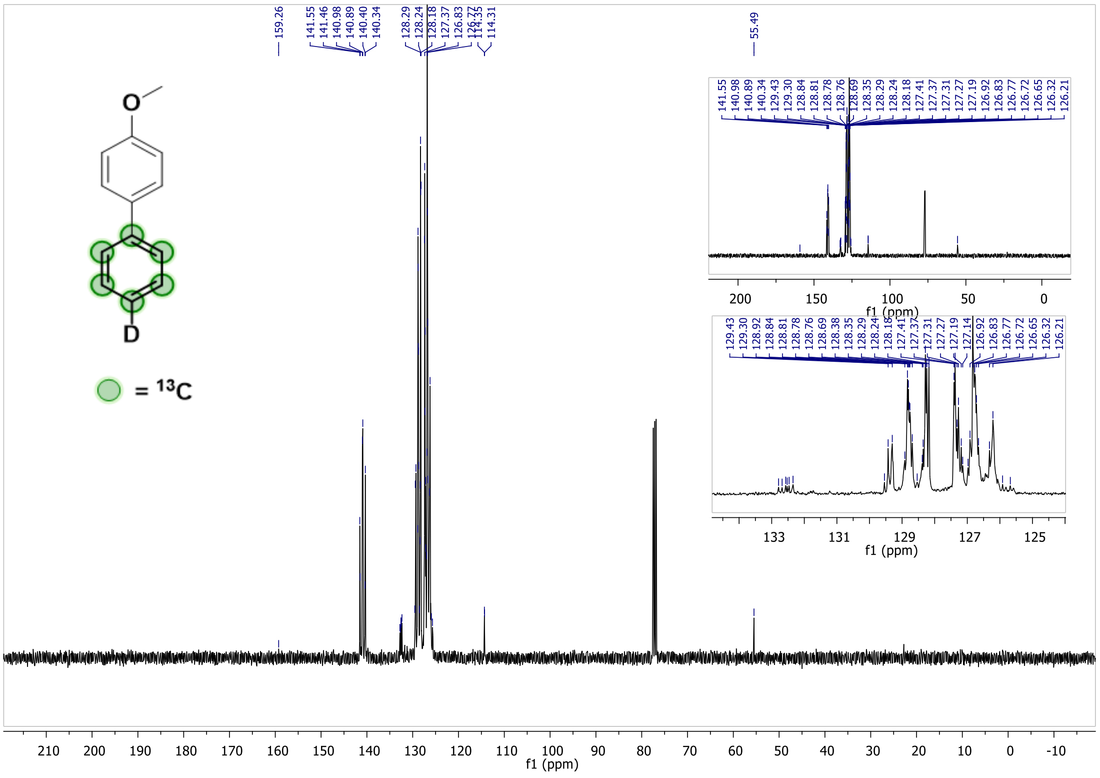

^2H NMR-{^1H}NMR (61 MHz, $CHCl_3$), 1-(4-methoxyphenyl)benzene-1,2,3,4,5,6-$^{13}C_6$-4-D (**[$^{13}C_6$, ^2H]24**)

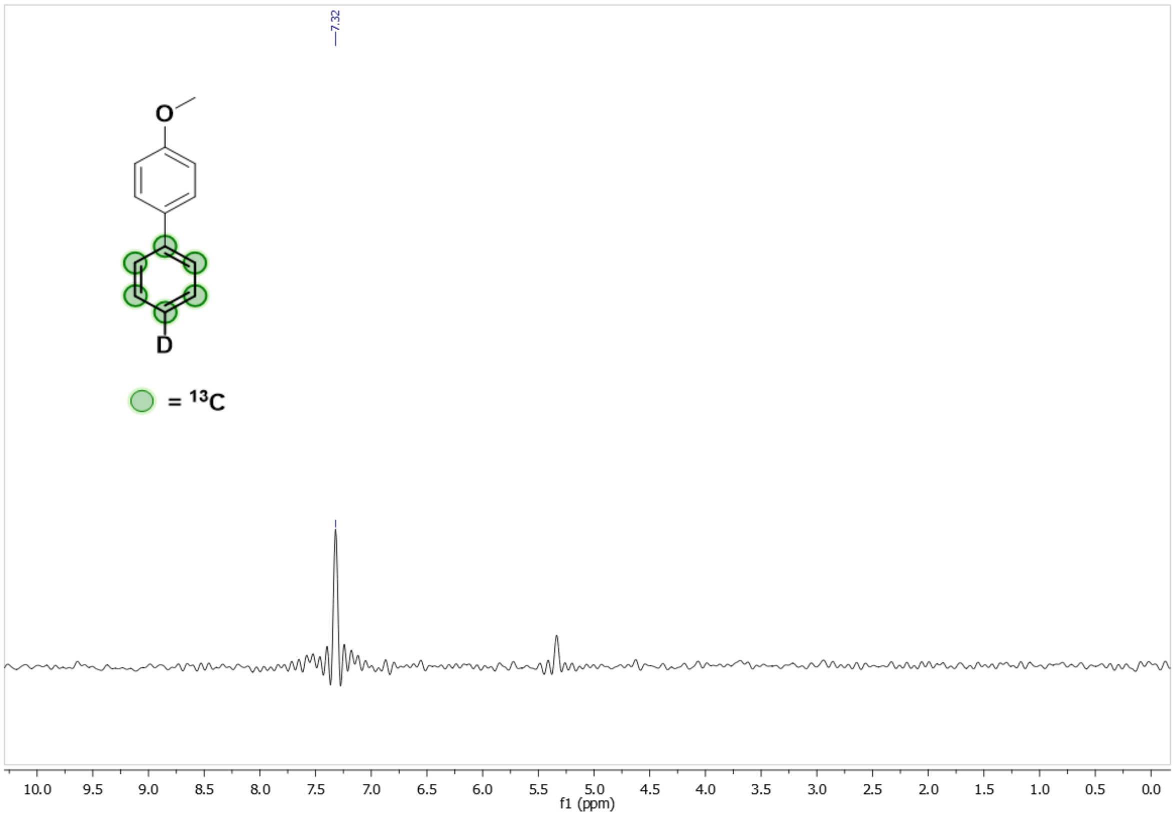

$^1H\{^{13}C\}$ NMR (400 MHz, $CDCl_3$), Ethyl (*E*)-3-(2,6-dibenzyl(phenyl-$^{13}C_6$)acrylate **([$^{13}C_6$]25)**

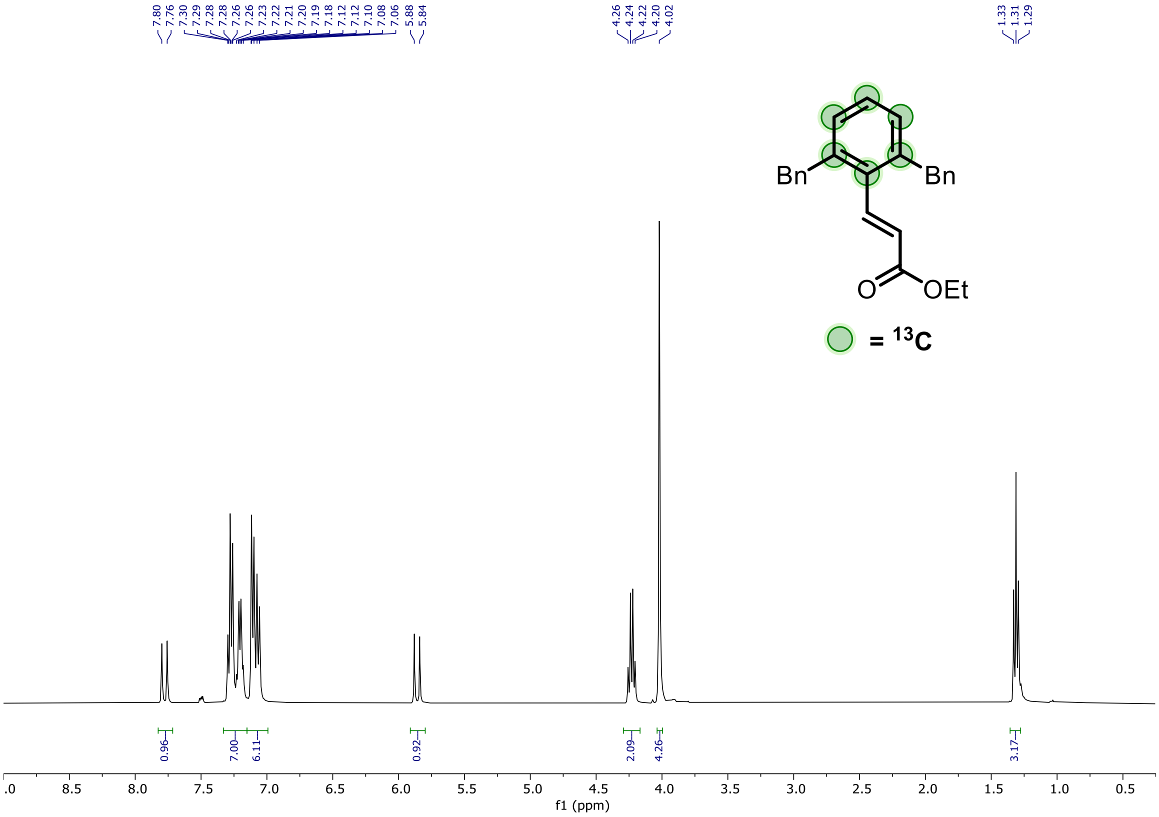

^{13}C NMR (100 MHz, $CDCl_3$), Ethyl (*E*)-3-(2,6-dibenzyl(phenyl-$^{13}C_6$)acrylate **([$^{13}C_6$]25)**

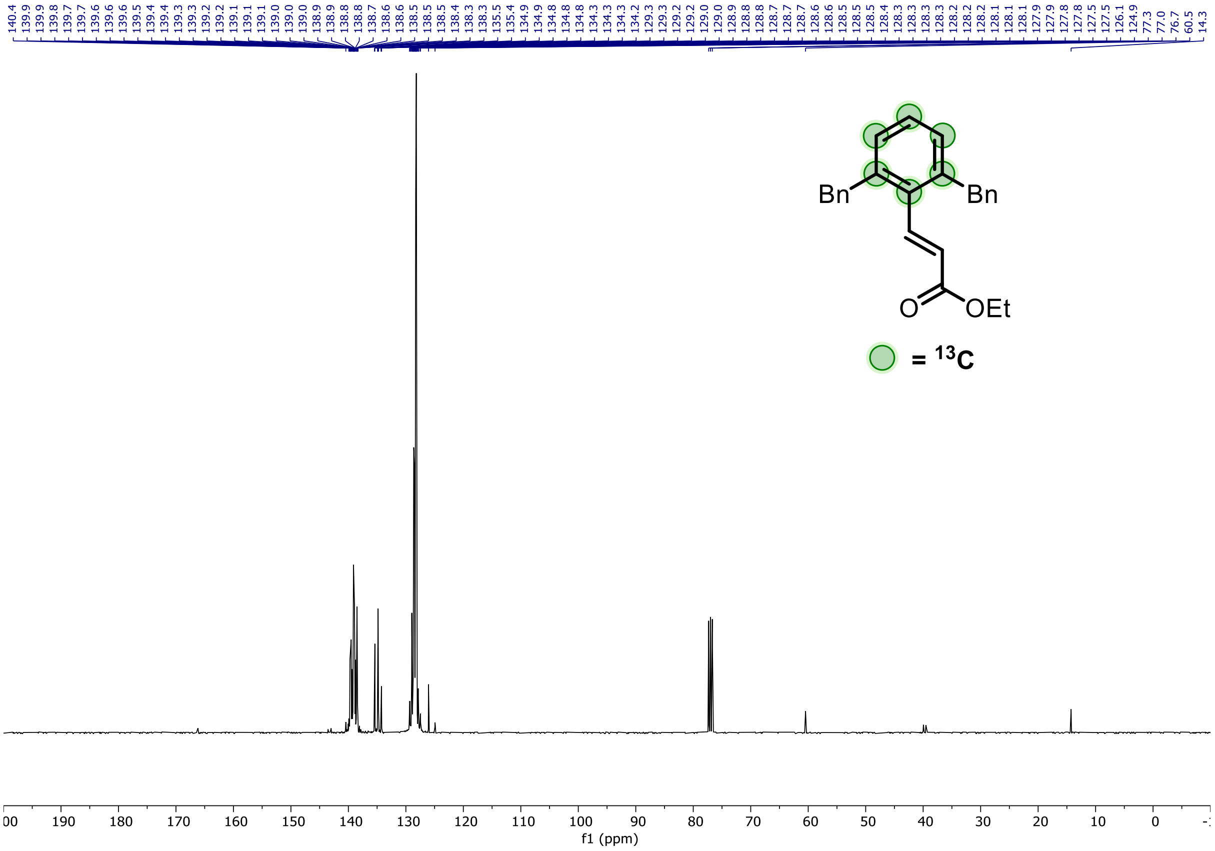

^{1}H NMR (400 MHz, $CDCl_3$), Ethyl *(E)*-3-(2,6-dibenzyl(phenyl-D_3)acrylate ([$^{2}H_3$]**25**)

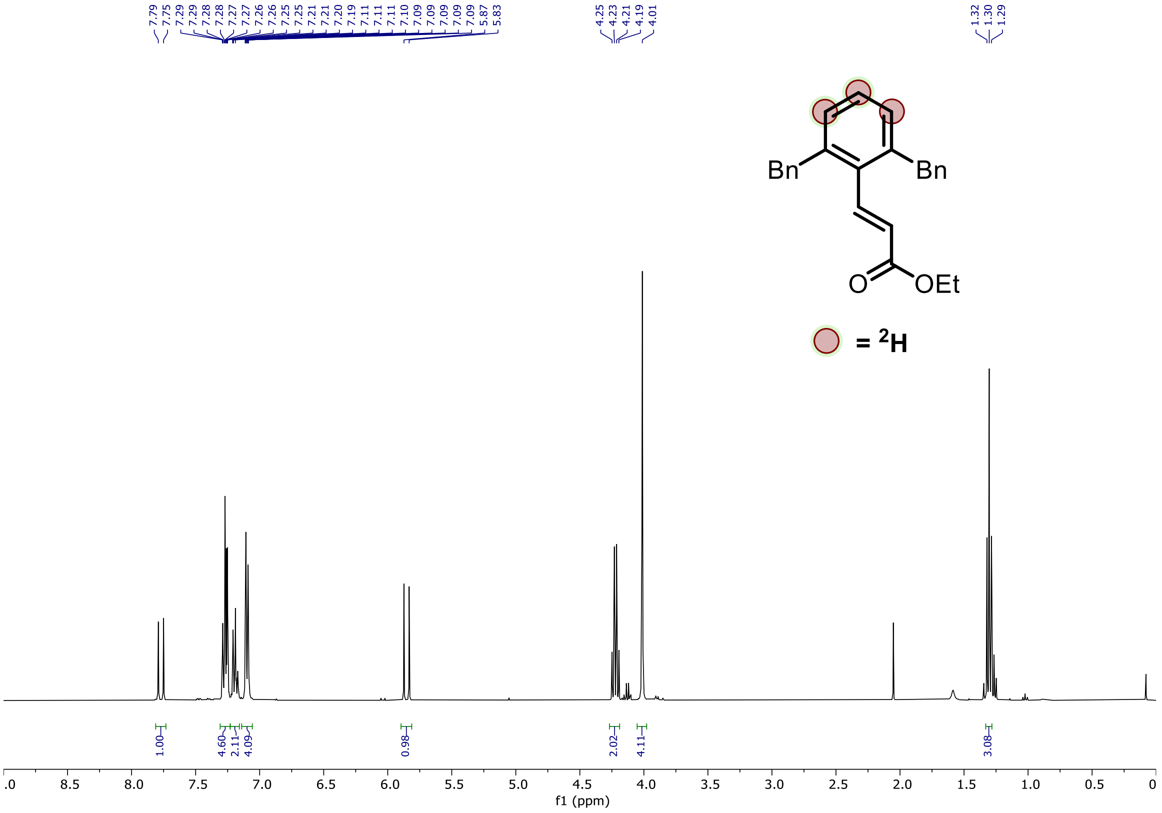

^{13}C NMR (100 MHz, $CDCl_3$), Ethyl *(E)*-3-(2,6-dibenzyl(phenyl-D_3)acrylate ([**$^2H_3$]25**)

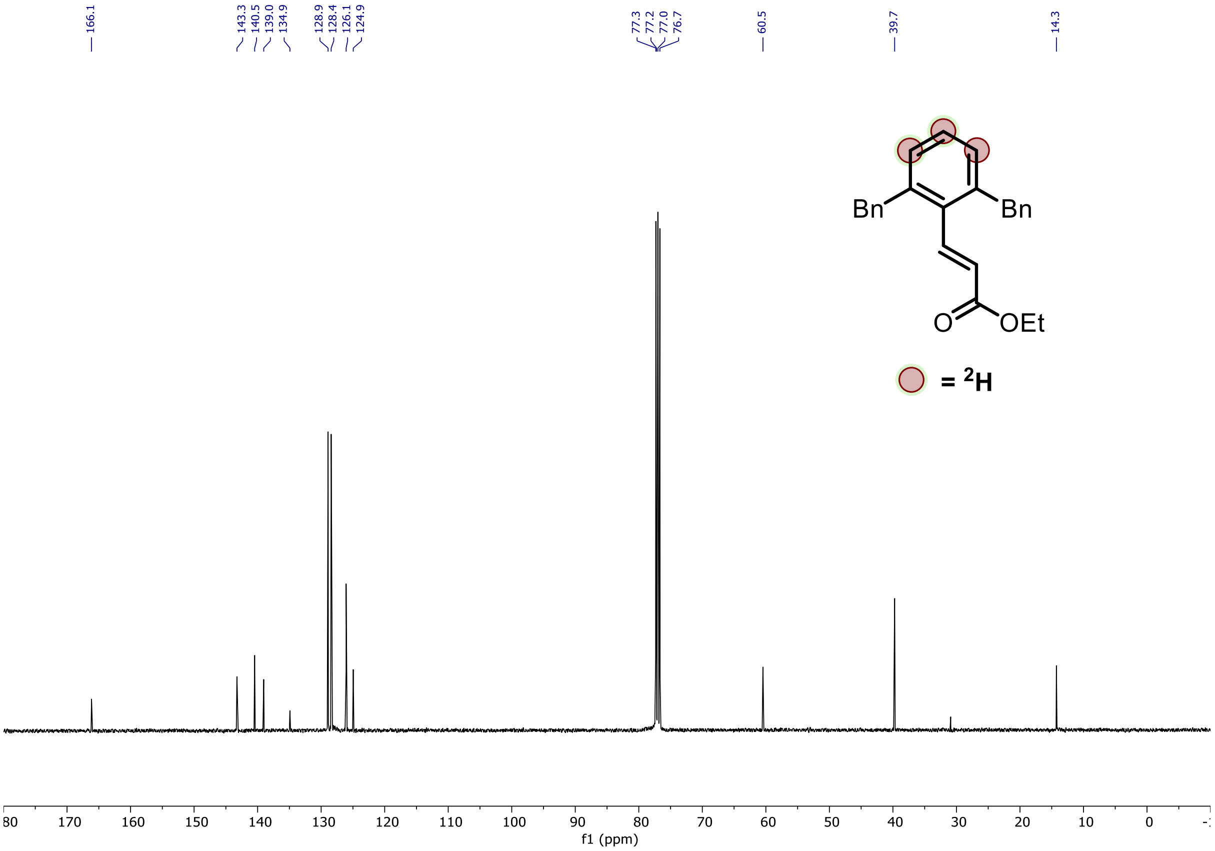

^{1}H{^{13}C} NMR (400 MHz, $CDCl_3$), 4-(2,6-Dibenzyl(phenyl-$^{13}C_6$))butan-2-one (**[$^{13}C_6$]26**)

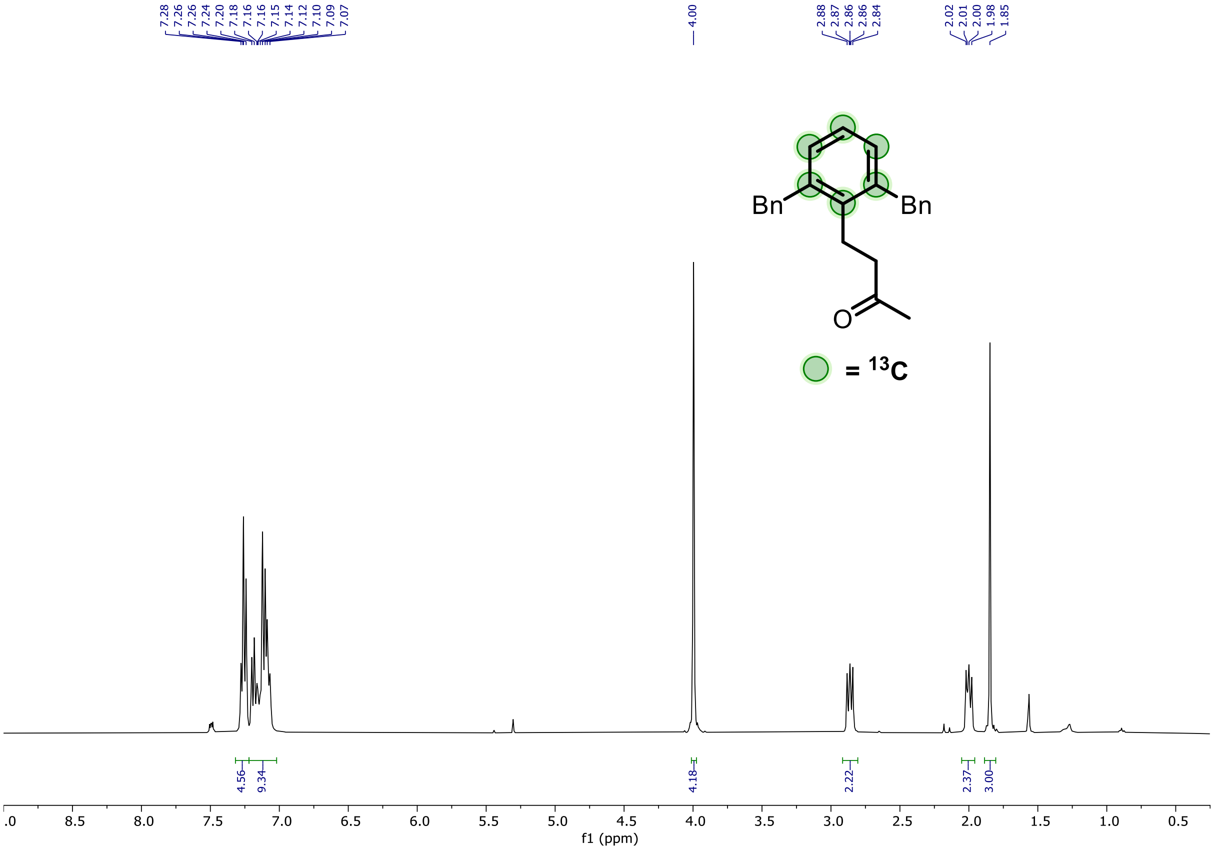

^{13}C NMR (100 MHz, $CDCl_3$), 4-(2,6-Dibenzyl(phenyl-$^{13}C_6$))butan-2-one (**[$^{13}C_6$]26**)

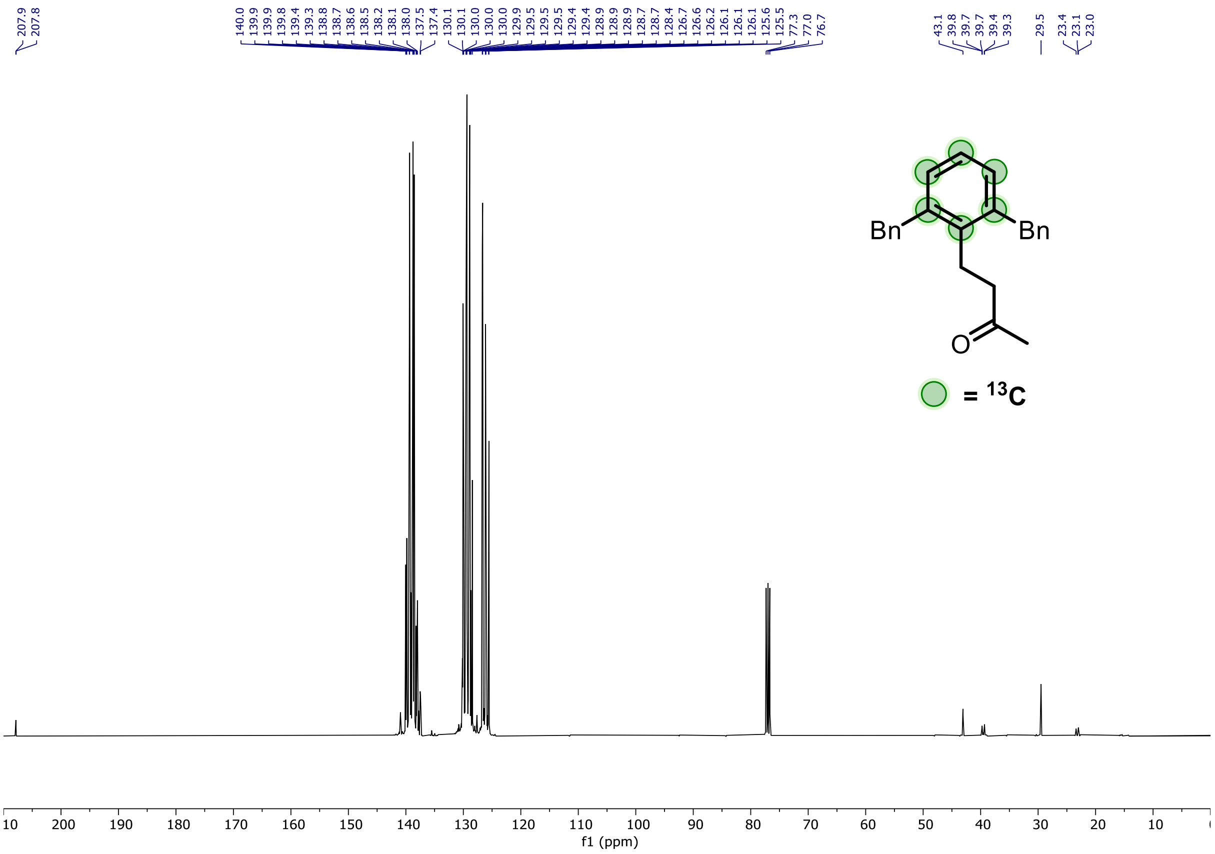

^{1}H{^{13}C} NMR (400 MHz, $CDCl_3$), Dimethyl 4,4'-(1,3-(phenyl-$^{13}C_6$)enebis(methylene)) dibenzoate (**[$^{13}C_6$]27**)

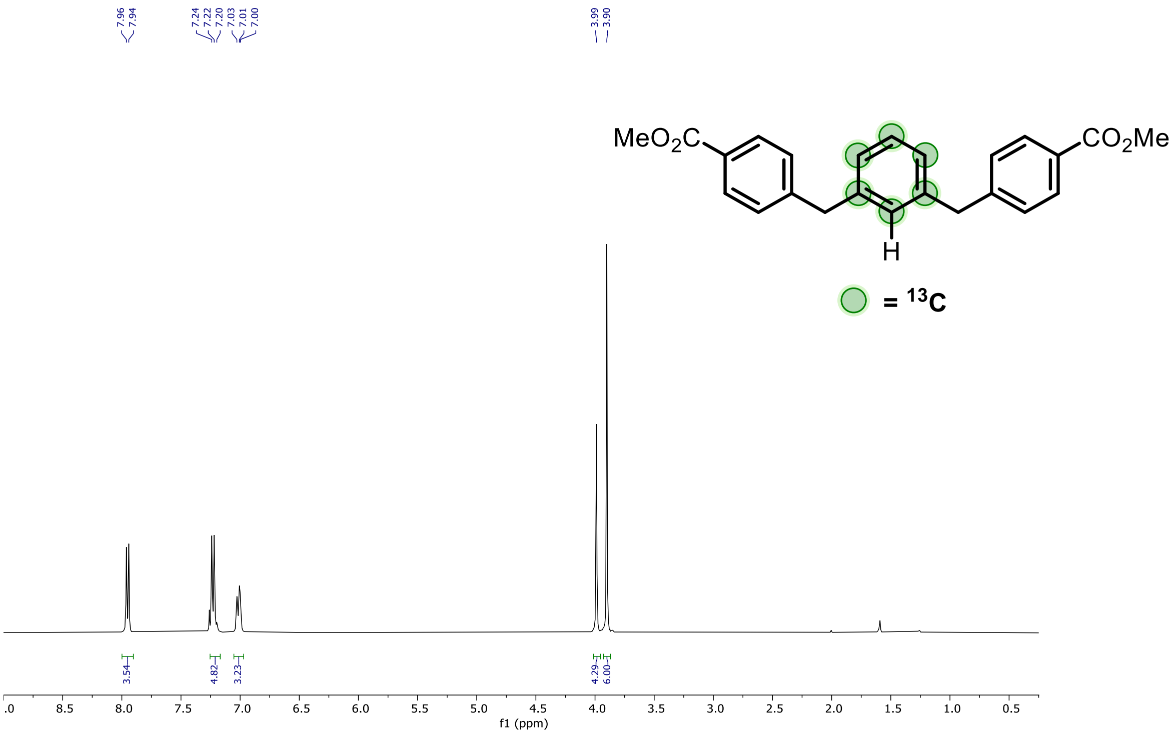

^{13}C NMR (100 MHz, $CDCl_3$), Dimethyl 4,4'-(1,3-(phenyl-$^{13}C_6$)enebis(methylene)) dibenzoate (**[$^{13}C_6$]27**)

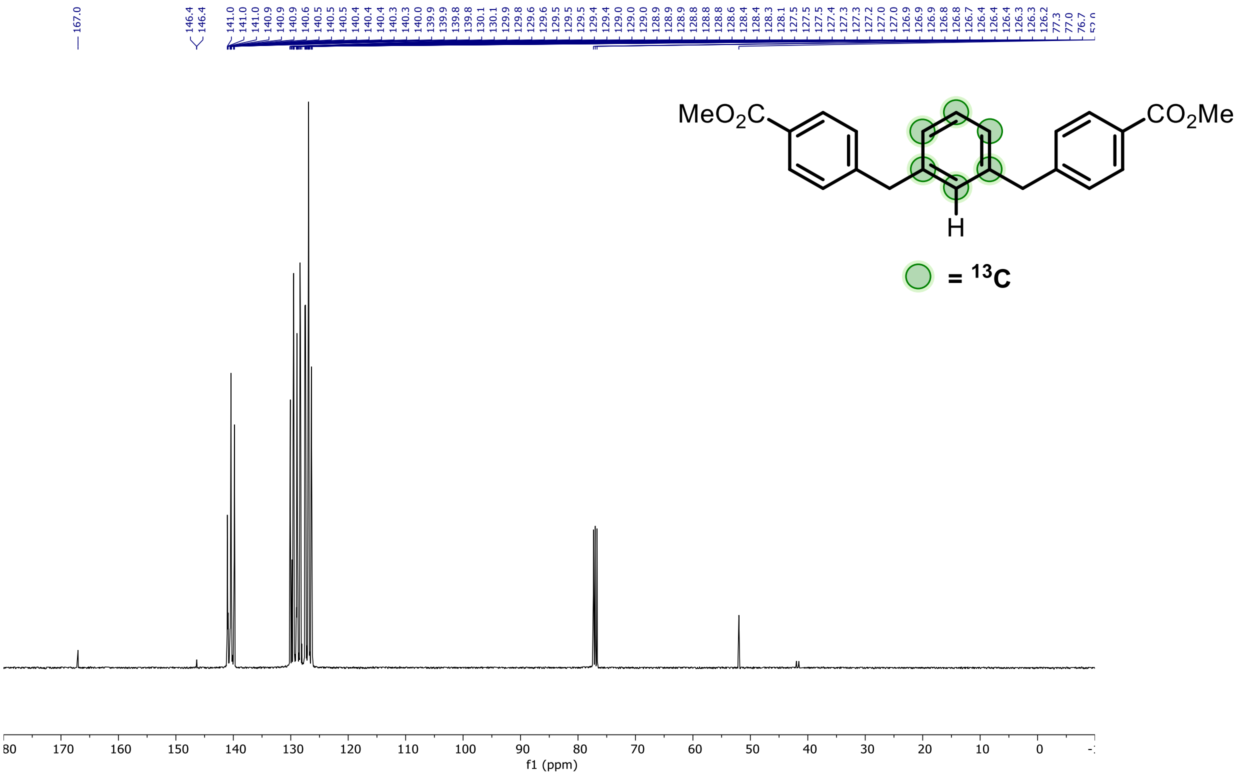

$^{1}H\{^{13}C\}$ NMR (400 MHz, $CDCl_3$), 1,3-Dimorpholino(benzene-$^{13}C_6$) (**[$^{13}C_6$]28**)

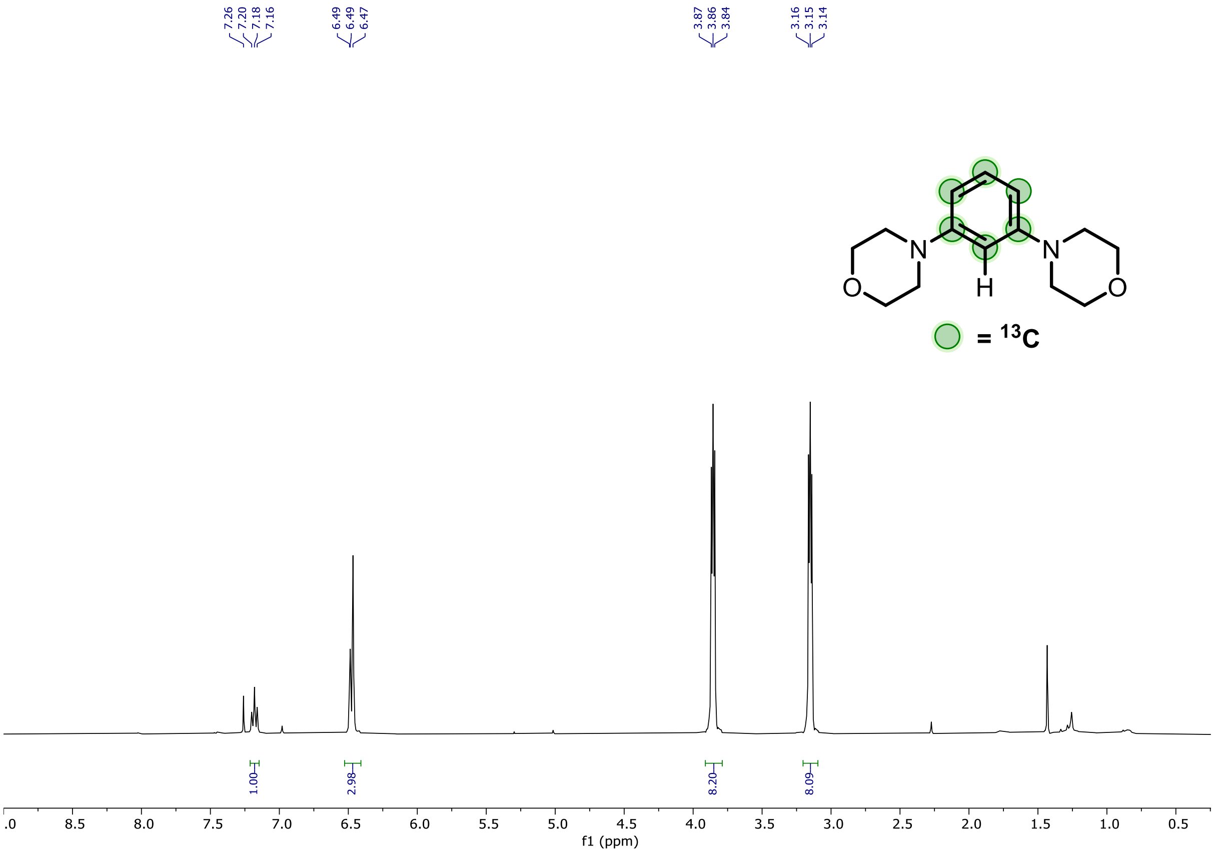

^{13}C NMR (100 MHz, $CDCl_3$), 1,3-Dimorpholino(benzene-$^{13}C_6$) (**[$^{13}C_6$]28**)

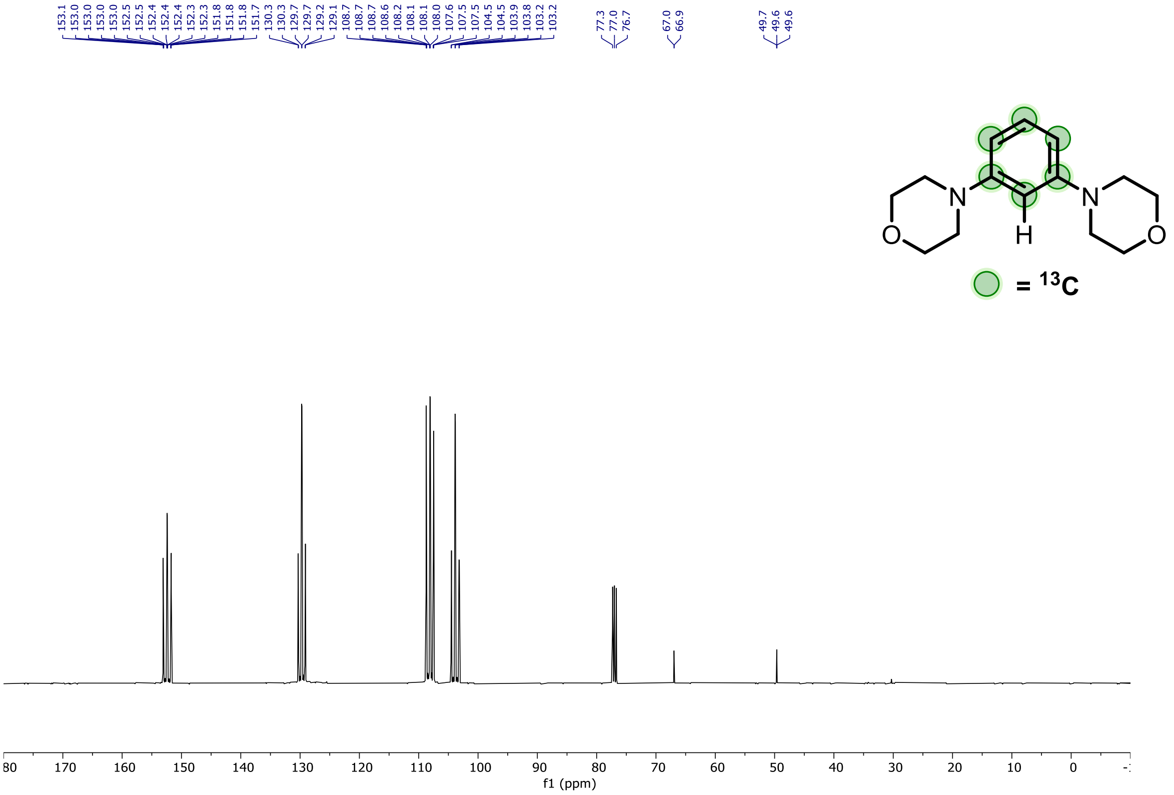

6. Reference

[1] G. Fraenkel, W. Burlant, *J. Chem. Phys.* **1965**, *42*, 3724–3725.
[2] J. R. Wesener, H. Günther, *Org. Magn. Reson.* **1983**, *21*, 433–435.
[3] G. Facey, *Finding "Lost" Deuterated ^{13}C Signals*. University of Ottawa NMR Facility Blog. https://u-of-o-nmr-facility.blogspot.com/2008/04/finding-lost-deuterated-13-c-signals.html (accessed 2025-01-02).
[4] K. L. Billingsley, T. E. Barder, S. L. Buchwald, *Angew. Chem. Int. Ed.* **2007**, *46*, 5359–5363.
[5] S. P. A. Hinkes, C. D. P. Klein, *Org. Lett.* **2019**, *21*, 3048–3052.
[6] Sheikh, M.; Iwasawa, T.; Nakajima, A.; Kitao, A.; Tsubaki, N.; Miyatake, R.; Yoshimura, T.; Morita, H. *Synthesis* **2014**, *46*, 42–48.
